# Phototoxicity of Half-Sandwich Rhodium(III) Complexes with Anthracene and Biphenyl Substituents toward Mammalian Cancer Cells and Multicellular Tumor Spheroids

**DOI:** 10.1021/acs.jmedchem.5c01406

**Published:** 2025-09-18

**Authors:** Petra Andršová, Jitka Prachařová, Vojtěch Novohradský, Slavomíra Šterbinská, Pavel Štarha, Jana Kašpárková, Viktor Brabec

**Affiliations:** † Department of Biophysics, Faculty of Science, 48207 Palacky University, Slechtitelu 241/27, CZ-77900 Olomouc, Czech Republic; ‡ Department of Inorganic Chemistry, Faculty of Science, 48207 Palacky University, 17. listopadu 1192/12, CZ-77146 Olomouc, Czech Republic; § Czech Academy of Sciences, Institute of Biophysics, Kralovopolska 135, CZ-61200 Brno, Czech Republic

## Abstract

We report the synthesis and characterization of three half-sandwich rhodium­(III) complexes incorporating anthracene and biphenyl moieties to investigate their photobiological activity. Complexes **1** and **2**, bearing anthracene-based Schiff base ligand, displayed pronounced phototoxicity upon blue-light irradiation, with complex **2** achieving a phototoxicity index of 144 in A375 melanoma cells. In contrast, complex **3**, lacking the anthracene unit, was photoinactive. Photoactivation of complexes **1** and **2** induced −N=CH– Schiff bond cleavage, releasing anthracene-9-carbaldehyde, as confirmed by UV–vis and mass spectrometry. Blue light also enabled catalytic oxidation of NADH to NAD^+^. Mechanistic studies revealed oncosis-like cell death mediated by reactive oxygen species. Both complexes retained activity in 3D tumor spheroids, demonstrating efficient tissue penetration. These results identify anthracene-functionalized rhodium­(III) complexes as promising candidates for photodynamic therapy and provide, to our knowledge, the first detailed mechanistic insight into photopotentiation in half-sandwich rhodium systems.

## Graphical abstract

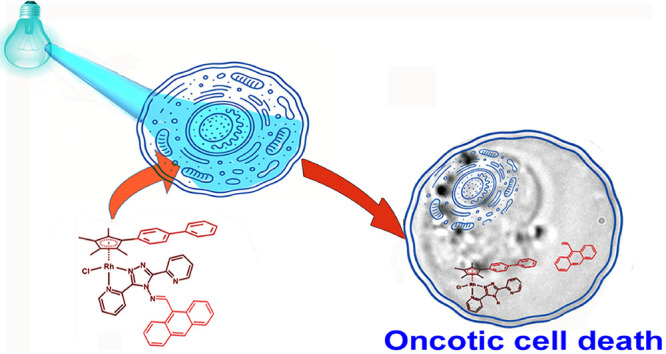

## Introduction

The study of metal-based compounds in oncology has been primarily dominated by platinum-based drugs, such as cisplatin, which have shown significant efficacy against various cancers. However, their severe side effects and the emergence of resistance have driven the search for alternative metal complexes. Initially, rhodium compounds were overlooked due to concerns about toxicity,^1^ limiting their exploration as therapeutic agents. Nevertheless, subsequent research has revealed their substantial anticancer potential.^2,3^ Among the first rhodium complexes considered as potential organometallic anticancer drugs were closely related congeners of Ru­(II)-arene-pta complexes (pta = phosphine 1,3,5-triaza-7-phosphatricyclo[3.3.1.1]­decane).^4^

Recent studies have highlighted rhodium­(III) complexes as promising cancer therapeutics. For example, rhodium­(III)-picolinamide complexes exhibit vigorous antiproliferative activity against various cancer cell lines. Mechanistic studies indicate that these complexes induce cell cycle arrest, apoptosis, and autophagy, while inhibiting metastasis by suppressing EGFR expression through the FAK-regulated integrin β1 pathway. Furthermore, experiments have confirmed their efficacy in reducing tumor growth and metastasis in bladder and breast cancer models.^5^

Another study examined rhodium­(III) complexes with isoquinoline derivatives, which demonstrated potent anticancer activity with minimal cytotoxicity toward noncancerous cells. These complexes induce apoptosis through mitochondrial dysfunction, characterized by increased reactive oxygen species (ROS) and calcium ion levels, which ultimately leads to the release of cytochrome c and caspase activation. *In vivo* assessments showed significant tumor growth inhibition in a T-24 xenograft mouse model.^6^

Additional organometallic rhodium complexes exhibit promising *in vitro* activity, particularly mixed-ligand complexes with the general formula [Rh­(η^5^-Cp*)­(N,N/O)­(N)]^2+/+^, where (N,N/O) represents bidentate N,N- or N,O-donor ligands such as 8-hydroxyquinolate (qui), ethylene-1,2-diamine, or 1,10-phenanthroline (phen), and (N) is a releasable monodentate nitrogen donor ligand; HCp* = pentamethylcyclopentadiene. These complexes were tested on drug-sensitive (Colo205) and resistant (Colo320) colon cancer cell lines, demonstrating cytotoxic effects. Notably, 8-hydroxyquinoline complexes were more potent than 1,10-phenanthroline derivatives. Some complexes also showed activation at acidic pH, as seen in [Rh­(η^5^-Cp*)­(phen)­(py/mim)]^2+^; py = pyridine, mim = 1-methylimidazole.^7^ Furthermore, [Rh­(η^5^-Cp*)­(qui)­Cl] displayed activity against melanoma and glioblastoma cells.^8^

The mechanisms underlying the antiproliferative effects of rhodium complexes are diverse. Some localize to mitochondria, disrupting function and inducing apoptosis via intrinsic pathways. Others act through mechanisms distinct from those of platinum-based drugs, offering potential solutions to resistance and toxicity issues.^9^ The potential of half-sandwich compounds in photodynamic therapy, which was recently published for Ir­(III) complexes,^10^ should also be considered for rhodium compounds and their future medicinal chemistry research.

In summary, rhodium complexes hold significant promise as novel anticancer agents due to their ability to induce apoptosis, autophagy, and inhibit metastasis. Further research into their mechanisms of action could pave the way for new, more effective cancer treatments.^5^

In this study, we synthesized and evaluated the biological activity of a new half-sandwich rhodium­(III) complex, [Rh­(η^5^-Cp^bph^)­Cl­(L1)]­PF_6_ (**1**; Figure 1), incorporating a triazole-based Schiff base abpt derivative with an anthracene substituent (L1 = N-[1-(anthracene-9-yl)-methylidene]-3,5-di­(pyridin-2-yl)-4*H*-1,2,4-triazol-4-amine; HCp^bph^ = (4-(2,3,4,5-tetramethylcyclopenta-2,4-dien-1-yl)­biphenyl); abpt = 3,5-di­(pyridin-2-yl)-4*H*-1,2,4-triazol-4-amine. To assess the effects of anthracene and biphenyl ligands, we also investigated the biological activity of [Rh­(η^5^-Cp*)­Cl­(L1)]­PF_6_ (**2**; Figure 1) and [Rh­(η^5^-Cp^bph^)­(abpt)­Cl]­PF_6_ (**3**; Figure 1).

**Figure 1:**
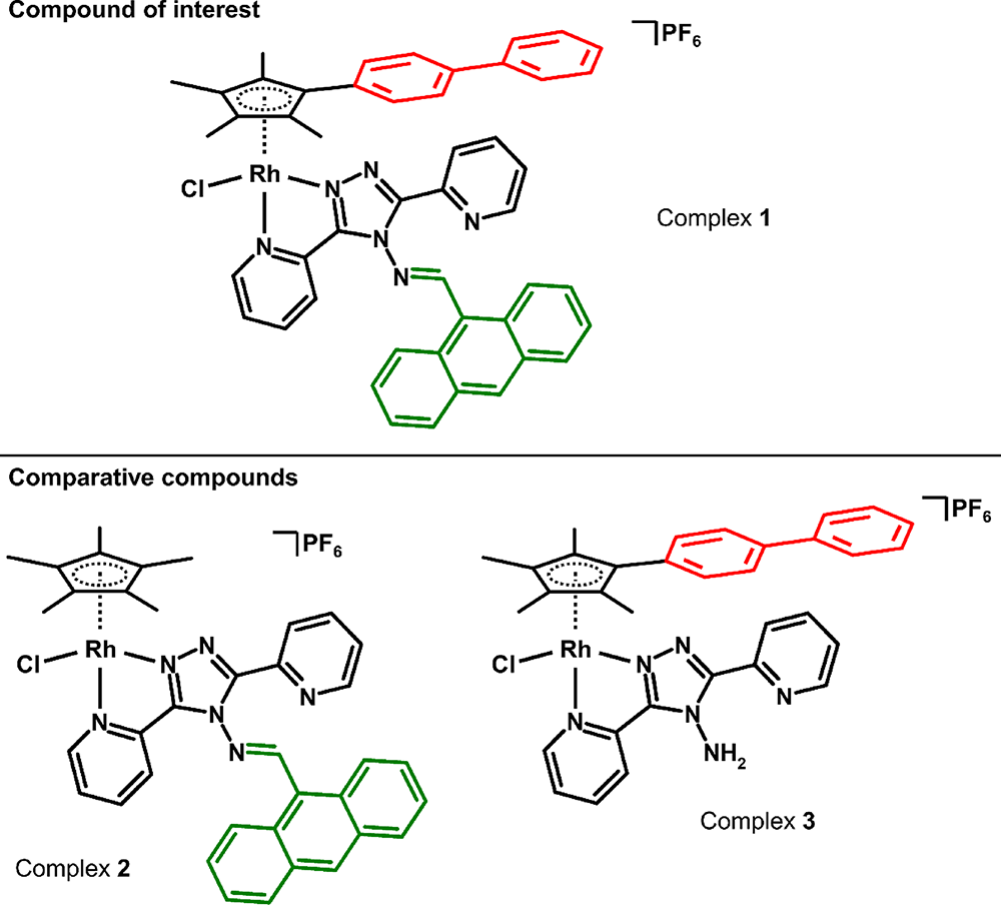
Structural formulas of Rh­(III) complexes [Rh­(η^5^-Cp^bph^)­Cl­(L1)]­PF_6_ (**1**), [Rh­(η^5^-Cp*)­Cl­(L1)]­PF_6_ (**2**), and [Rh­(η^5^-Cp^bph^)­(abpt)­Cl]­PF_6_ (**3**); HCp^bph^ = (4-(2,3,4,5-tetramethylcyclopenta-2,4-dien-1-yl)­biphenyl), L1 = N-[1-(anthracene-9-yl)-methylidene]-3,5-di­(pyridin-2-yl)-4H-1,2,4-triazol-4-amine, HCp* = pentamethylcyclopentadiene, and abpt = 3,5-di­(pyridin-2-yl)-4H-1,2,4-triazol-4-amine.

## Results and Discussion

### Synthesis, Characterization, and Stability Studies

The organic compound L1 was prepared by a Schiff condensation reaction between abpt and anthracene-9-carbaldehyde (AC) in MeOH acidified by acetic acid, representing a slight modification of its formerly reported synthesis.^11^ Coordination compounds [Rh­(η^5^-Cp^bph^)­Cl­(L1)]­PF_6_ (**1**), [Rh­(η^5^-Cp*)­Cl­(L1)]­PF_6_ (**2**) and [Rh­(η^5^-Cp^bph^)­(abpt)­Cl]­PF_6_ (**3**) were designed to represent a trio of compounds incorporating the double-substituted complex **1** involving two photosensitive substituents (i.e., anthracene on L1 and biphenyl on Cp^bph^) and its derivatives **2** and **3** bearing only one of the two photosensitive substituents (see Figure 1). Complexes **1**–**3** were prepared by a typical synthetic procedure using appropriate dimers [Rh­(μ-Cl)­(η^5^-Cp*)­Cl]_2_ (for **2**) and [Rh­(μ-Cl)­(η^5^-Cp^bph^)­Cl]_2_ (for **1** and **3**) and their interaction with a chelating ligand L1 (for **1** and **2**) or abpt (for **3**) in a microwave reactor.

^1^H NMR spectrum of L1 contained the characteristic singlet of the C13–H Schiff bond (10.13 ppm; Figure S1). Resonances of hydrogen atoms in aromatic C–H groups of pyridines and anthracene were observed at 7.53–8.88 ppm with appropriate integral intensities. Regarding L1-containing complexes **1** and **2**, the C13–H resonance was detected at lower fields (10.42 and 10.38 ppm, respectively; Figures S2 and S3) than for free L1. This resonance is missing in the spectrum of abpt-derived complex **3** (Figure S4). As two cyclopentadienyls were used for **1**–**3**, differences were also detected in the higher fields of the ^1^H NMR spectra, which contained resonances of aliphatic hydrogen atoms, i.e., methyl groups of Cp^bph^ (for **1** and **3**) and Cp* (for **2**). For Cp*, a characteristic singlet was found in the spectrum of complex **2** (δ = 1.82 ppm). In contrast, ^1^H NMR spectra of Cp^bph^-based complexes **1** and **3** showed a set of aliphatic signals at 1.80–1.99 ppm.

Electrospray Ionization Mass Spectrometry (ESI-MS) proved the structural integrity of complex cations of **1**–**3**, because the dominant signals assignable to the [RhCl­(Cp^x^)­(L)]^+^ and {[Rh­(Cp^x^)­(L)] – H}^+^ species were detected (Figure S5; Cp^x^ = Cp^bph^ or Cp*, L = L1 or abpt). In the case of **1**, the mentioned characteristic signals, [RhCl­(Cp^bph^)­(L1)]^+^ and {[Rh­(Cp^bph^)­(L1)] – H}^+^, were accompanied by less intensive peaks belonging to [Rh­(bpt)­Cl­(Cp^bph^)]^+^ (634.1 *m*/*z*) and its dechlorinated fragment {[Rh­(bpt)­(Cp^bph^)] – H}^+^ (598.2 *m*/*z*); bpt = 2,2’-(4*H*-1,2,4-triazole-3,5-diyl)­dipyridine. This observation highlighted the lability of the N–N bond of L1 between the triazole and exocyclic nitrogen under the used electrospray ionization conditions. Similar fragmentation to bpt-containing species was observed by ESI-MS for the second of the studied L1-containing complexes (**2**) but not for the abpt-based complex **3**. The purity of complexes **1**–**3** was also verified by reversed-phase high-performance liquid chromatography (RP-HPLC) and elemental analysis. HPLC showed >95% purity of the complexes in water:ACN mixtures (Figure S6). In the case of complexes **2** and **3**, one peak was detected in the obtained chromatograms for trifluoroacetic acid (tfa) containing mobile phase at *t*_R_ = 9.88 and 8.85 min, respectively, assigned by a coupled mass spectrometry to the [Rh­(CF_3_COO)­(Cp^x^)­(L)]^+^ species (777.1 *m*/*z* for **2** and 727.0 *m*/*z* for **3**). On the other hand, the HPLC chromatogram of **1** showed two peaks, which belong to [Rh­(CF_3_COO)­(Cp^bph^)­(L1)]^+^ (*t*_R_ = 12.37 min) and [RhCl­(Cp^bph^)­(L1)]^+^ (*t*_R_ = 16.13 min). This indicated that the chlorido ligand of **1** is less labile toward tfa, as compared with **2** and **3**. Similar results were obtained for **1** when 0.1% formic acid was used instead of tfa (Figure S7). Since the two peaks detected in acidified water:ACN mixtures originate from one compound, their peak areas were counted together, yielding the mentioned >95% purity. In an effort to obtain a typical HPLC result with only one peak for **1**, ammonium formate was used instead of tfa and formic acid. In this case, only one peak was detected at *t*_R_ = 17.10 min, with a peak area greater than 95%, which belongs to the [RhCl­(Cp^bph^)­(L1)]^+^ chlorido species (Figure S6B).

Moreover, elemental analysis was performed, yielding differences of less than 0.4% between the calculated and experimental percentages of the determined elements (C, H, N; see Experimental Section).

Complexes **1**–**3** (^1^H NMR, 250 μM final concentration) were stable in the mixture of 45% DMF-*d*_7_/55% PBS in D_2_O in the dark, as no changes were detected up to 6 h of standing at r.t. (Figures S8–S10). Only traces of two metabolites (less than 3% in total) were observed after 24 h of standing at room temperature, corresponding to a free (released) aldehyde (δ = 11.58 ppm for **1** and **2**) and solvolysed complex species (δ = 10.06 ppm for **1** and 10.07 ppm for **2**). For **3**, no changes were detected even after 24 h. UV–vis spectroscopy showed (Figure S11) that complexes **1**–**3** (50 μM final concentration) were stable in 1% DMF in water (without or with PBS, in the dark), i.e., under the conditions used for most of the below discussed spectral experiment. In these samples, the integrity of the studied complexes in the presence of water was further confirmed by mass spectrometry, which detected dominant peaks corresponding to the [RhCl­(Cp^*x*^)­(L)]^+^ species. These chlorido species were not detected in the samples prepared with the addition of a stoichiometric amount of silver­(I) triflate (see Figure S12).

### Antiproliferative Activity

The ability of the Rh-complexes to affect the proliferation and viability of cancer cells was tested using a panel of four cancerous cell lines: human, highly aggressive, invasive, and poorly differentiated triple-negative breast cancer cells MDA-MB-231, human colorectal carcinoma (HCT116), human ovarian adenocarcinoma (A2780), and human epithelial lung carcinoma cells (A549). As indicated in Table 1, the Rh-complexes showed moderate activity with IC_50_ values in the range of tens of micromoles per liter. Among them, **1** showed the highest potency, comparable to that of cisplatin in MDA-MB-231 and HCT116 cell lines.

**Table 1:** Antiproliferative Activity [IC_50_ Mean Values (μM)] Obtained for Rh-Complexes and Cisplatin by MTT after 72 h Treatment^a^

	MDA-MB-231	HCT116	A2780	A549
**1**	18 ± 6	9.1 ± 0.7	13.5 ± 0.5	12.4 ± 0.6
**2**	49 ± 2	26 ± 2	25 ± 3	21.1 ± 0.2
**3**	89 ± 9	50.5 ± 0.9	22 ± 5	83 ± 14
cisplatin	25 ± 2	9 ± 2	2.90 ± 0.03	4.1 ± 0.2

a The results are expressed as mean values ± SD of three independent experiments, each performed in triplicate.

### Intracellular Accumulation and Localization

Drug accumulation, distribution, and localization represent essential factors determining the effectiveness of biologically active molecules. Understanding the intracellular localization of these compounds is crucial for elucidating their mechanisms of action, as the spatial distribution within the cell determines their accessibility to specific molecular targets, through which they exert their biological effects. Therefore, intracellular localization studies represent a fundamental step in the comprehensive evaluation of biologically active molecules.

To determine the intracellular localization of the investigated Rh-complexes, we utilized their fluorescent properties. Laser confocal microscopy was employed to capture fluorescence images of A375 cells incubated with the complex for 3 h in the dark. The samples were examined using a confocal laser-scanning microscope with an excitation wavelength of 405 nm and an emission detection range of 450–750 nm. It is necessary to mention here that under these conditions, only complex **1** exhibits a sufficiently strong fluorescence signal. In contrast, complexes **2** and **3** show only weak or no fluorescence, respectively (*vide infra*). In addition, the high accumulation in the cells contributed to the fact that the fluorescence signal of complex **1** could be detected with sufficient clarity. In this chapter, we therefore present the results of intracellular accumulation and localization for complex **1**, which serves as a representative of the investigated Rh complexes.

The results indicated that the fluorescence signal from **1** was primarily localized outside the cell nucleus, predominantly in the cytoplasm (Figure 2). This suggests that nuclear DNA is unlikely to be the main pharmacological target of these complexes, as is the case with the conventional antitumor drug cisplatin. However, it does not entirely rule out the role of DNA in the mechanism of action, although it is unlikely to be the only and major molecular target.

**Figure 2:**
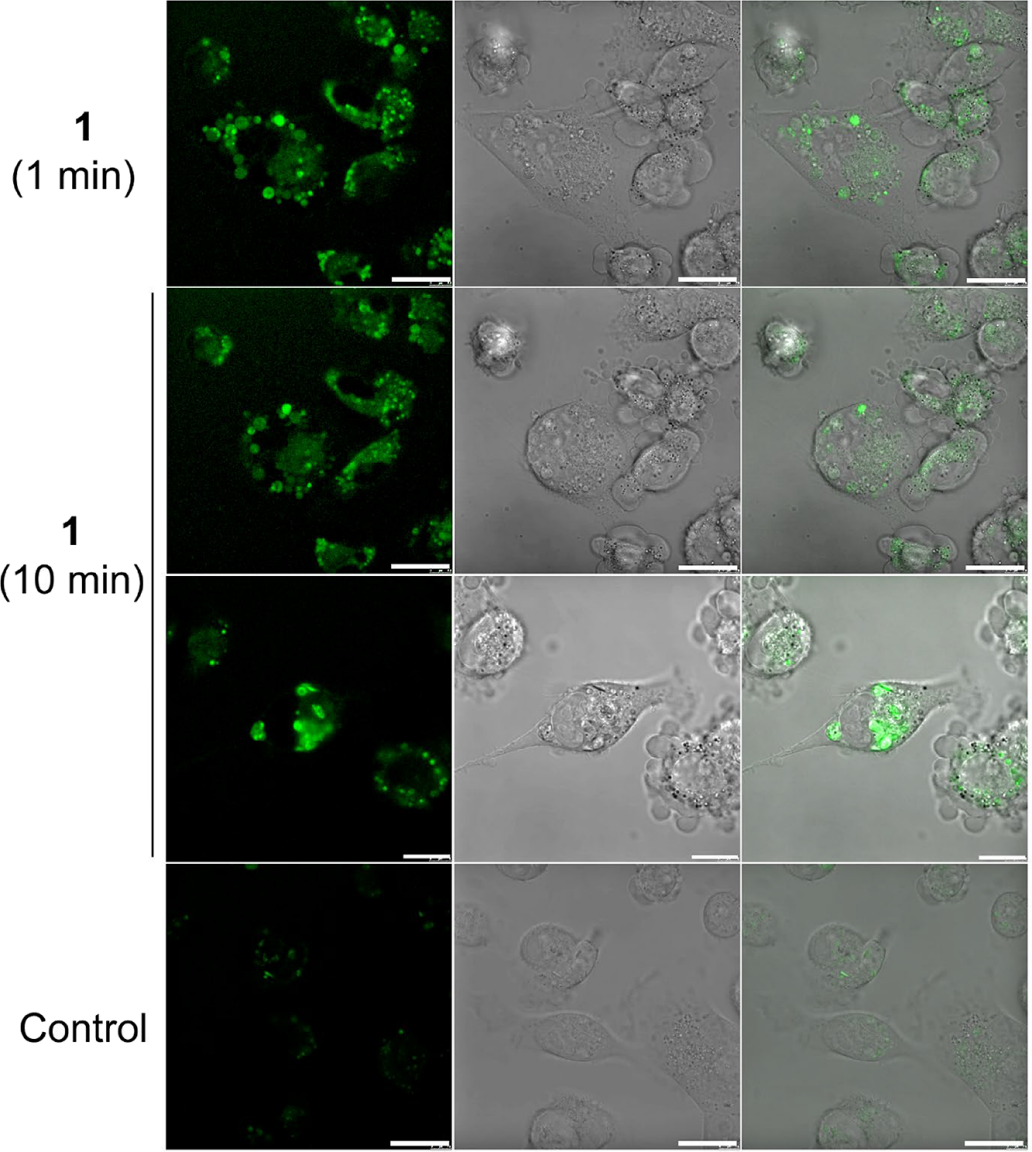
Cellular localization of **1** in HCT116 cells. Cells were treated with **1** (27 μM) for 3 h in the dark and then irradiated with a laser and captured 1 min (top row) or 10 min (second and third rows) after irradiation. Bottom row, untreated control cells. Channels: left, fluorescence signal; central, bright field; and right, overlay of the bright field and fluorescence channels. Scale bars indicate 20 μm.

In the cytoplasm, a diffuse signal is observed, along with distinct spots, indicating the presence of endosomes and suggesting that complex **1** is actively transported through the membrane. Colocalization experiments were performed to more precisely determine possible target organelles of complex **1** within the cell. The results did not reveal any significant colocalization of the signal originating from **1** with either mitochondria or the endoplasmic reticulum (Pearson’s correlation coefficients 0.528 ± 0.136 and 0.318 ± 0.087 for mitochondria and ER, respectively). Instead, the complex appears to be rather nonspecifically distributed within the cytoplasm (Figure S13).

To verify whether the observed spots could be related to potential aggregation/clustering of complex **1** due to the presence of extended aromatic systems, nanoparticle tracking analysis (NTA) was performed. The results showed that upon dilution of the stock solution prepared in DMF into an aqueous environment, particles of approximately 120 nm in size were detected, and their size did not change significantly after 24 h of incubation (Figure S14). However, the spots observed in the confocal microscope are substantially larger (approximately 1–4 μm) and therefore cannot correspond to the aggregates detected by NTA. Nevertheless, the obtained results further support the hypothesis that the cellular uptake of compound **1** is likely mediated by endocytosis, consistent with the known pathways for nanoparticle internalization.

Significant changes in cell morphology were observed during the observation of live cells using a confocal microscope. The epithelial-like morphology, initially characterized by polygonal and elongated shapes, gradually transformed, with cells adopting a more rounded shape and exhibiting membrane protrusions (blebbing). This process was relatively rapid, occurring within approximately 10–15 min (Figures 2 and S15). No such changes were observed for the control, untreated cells. Since fluorescence signal acquisition involves exposing the samples to excitation light, the observed phenomenon suggests a possible effect of irradiation on the biological activity of **1.**

### Phototoxic Activity

The previously described observation prompted us to investigate the potential effect of light on biological activity. Moreover, half-sandwich Rh­(III) complexes of similar structures have already been shown to act as photosensitizers.^12^ Furthermore, both anthracene and biphenyl substituents have been associated with induced photocytotoxicity.^13,14^ Therefore, the impact of light on activities of the Rh-complexes **1**–**3** was determined using light with a wavelength of 420 nm, i.e., in the region where the complexes exhibit absorption. For this experiment, the model cell line A375, derived from human skin melanoma, has been selected, as skin tumors are predisposed to phototherapy due to their accessibility to irradiation.

The experiment followed a previously described protocols.^15,16^ Briefly, A375 cells were exposed to the tested Rh complexes in Earle’s Balanced Salt Solution (EBSS) for 1 h in the dark to enable cellular uptake. Subsequently, the cells underwent either 1 h of irradiation with blue light (LZC-4 photoreactor equipped with 16 LZC-420 lamps, λ_max_ = 420 nm, 50 W m^–2^) or sham irradiation. After the treatment, the EBSS containing the Rh complex was removed, and the cells were allowed to recover in a complete, drug-free medium for 70 h. Cellular metabolic activity (proportional to the number of viable cells) was determined by a commonly used MTT assay.

As indicated in Table 2, two of the studied complexes containing an anthracene moiety, i.e., **1** and **2**, exhibited a significant phototoxic effect with IC_50_ values in the submicromolar range. Notably, they demonstrated significantly lower toxicity in the absence of irradiation, resulting in high phototoxicity indexes (PTI). Remarkably, **2**, due to its lower activity in the dark, attained a superior value of PTI = 144. In contrast, the only report on photocytotoxic half-sandwich Rh­(III) complexes mentioned only a marginal difference (phototoxic index, PTI < 5) in activity in the dark and irradiated cells (λ = 400 nm; 4 h incubation +30 min irradiation (30 mW cm^–2^) + 24 h incubation).^12^

**Table 2:** (Photo)­cytotoxicity (IC_50_ Values, [μM]) Obtained for A375 Cells Treated with the Rh-Complexes for 1 h and Irradiated by Blue Light (1 h, λ_max_ = 420 nm, and 56 ± 2 W m^–2^) or Sham Irradiated as Determined by the MTT Assay 70 h after Irradiation^a^

	A375			MRC-5 pd30 (72 h incubation)
	irrad.	sham irrad.	PTI^b^	
**1**	0.20 ± 0.03	7.1 ± 0.5	36	15 ± 1
**2**	0.18 ± 0.02	26 ± 1	144	30 ± 5
**3**	33 ± 3	31 ± 2	0.9	57.1 ± 0.9

a The data are expressed as mean values ± SD from at least three independent experiments, each performed in duplicate.

b PTI (Phototoxicity index) was calculated using the following formula: PTI = IC_50_ (sham irrad)/IC_50_ (irrad).

This indicates a substantial enhancement in the biological activity of complexes **1** and **2** against cancer cells, with complex **2** showing particular promise. These findings suggest that both complexes, especially complex **2**, could be promising candidates for further investigation in photoactivated cancer therapy. Conversely, **3** does not demonstrate any photopotentiation when irradiated under the same conditions. This suggests that the derivatization of L1, specifically the attachment of the anthracene substituent via Schiff base formation, is crucial for photoactivation. In contrast, the second photosensitive substituent (the biphenyl group of the Cp^bph^ ligand) appears to offer no additional benefit regarding photoactivation and photocytotoxicity.

The effect of nonirradiated Rh complexes on human noncancerous cells was also determined, specifically against human lung fibroblast MRC-5 cells. Notably, complex **1** showed promising antiproliferative selectivity for nonirradiated cancerous melanoma cells, as confirmed by the fact that its effect was significantly lower even after long-term exposure, when the MRC-5 cells were continuously exposed to the complex for 72 h.

### Photophysical Properties

The absorption and emission spectra of **1–3** in PBS buffer (1% DMF) are presented in Figure 3. Complexes **1** and **2** exhibit absorption in the UV region, and an absorption band in the visible region (λ_max_ = 425–430 nm) is also apparent for both. In contrast, no such absorption band is observed for **3**. This band can be attributed to the anthracene moiety, a conclusion supported by DFT calculations (*vide infra*) and a control UV–vis experiment that displayed the same absorption maximum for free AC (Figure S16). Regarding DFT, this broad band centered at ca. λ = 425 nm for **1** and **2** results from multiple HOMO → LUMO (donor → acceptor) electronic transitions that involve the anthracene substituent (note: calculations were performed only for **1**). Specifically, the results involved n → π* transitions localized within the anthracene substituent, while other transitions are directly connected with the presence of the anthracene substituent in L1. For example, intraligand charge transfer (ILCT) from the anthracene substituent to bpt, ligand-to-ligand charge transfer (LLCT) from biphenyl to anthracene, metal-to-ligand charge transfer (MLCT) to the anthracene moiety, or LLCT and LMCT transitions from anthracene to the cyclopentadienyl ring and metal center, respectively, contributed the most to the resulting band centered at ca. λ = 425 nm (Table S1).

**Figure 3:**
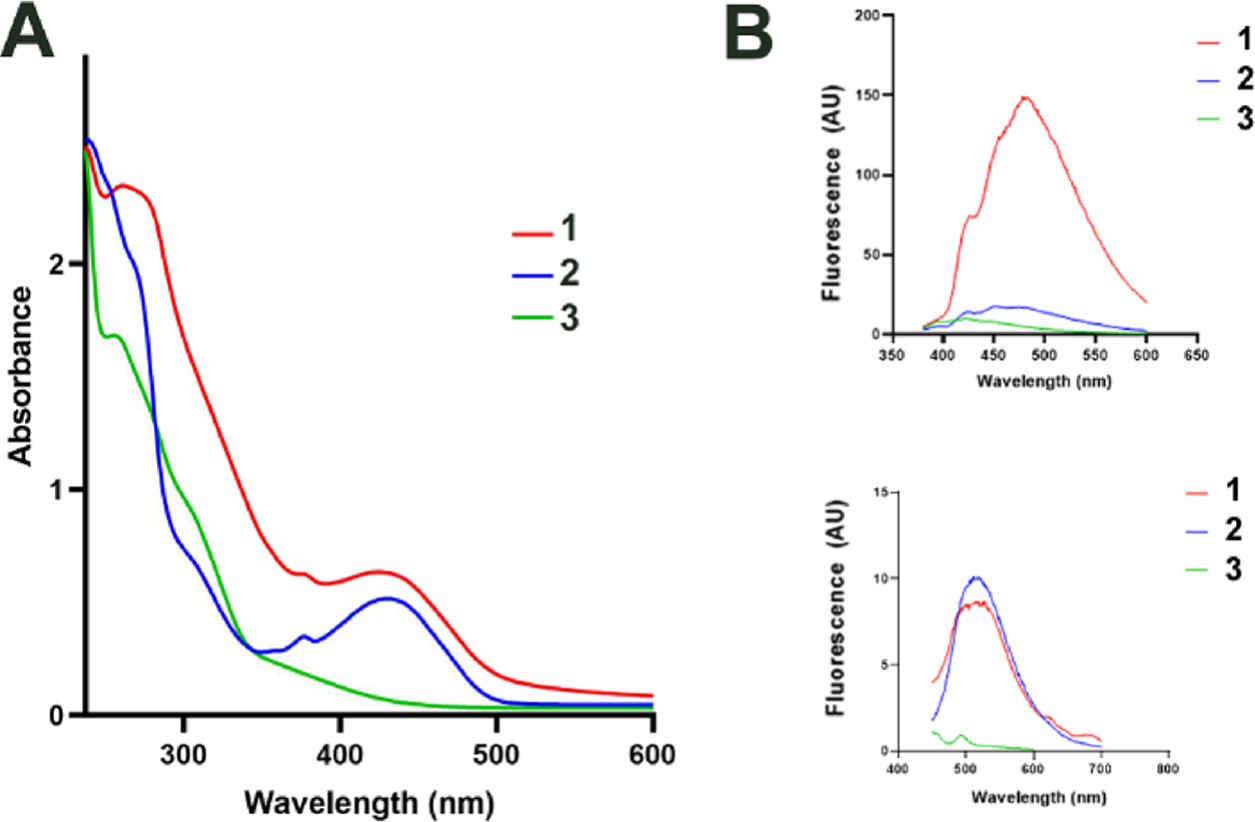
(A) UV/vis absorption spectra of **1**–**3** (50 μM in water (1% DMF)). (B) Emission spectra of **1**–**3** (30 μM) in DMF. Top panel, λ_exc_ = 350 nm; bottom panel, λ_exc_ = 430 nm).

When excited at 350 nm, the complex **1** in 100% DMF exhibits a fluorescence signal with a maximal emission around 480 nm (Figure 3B, top panel). The absolute emission quantum yields (ϕ_em_) equaled 0.049 for **1**. The other two complexes, **2** and **3**, show only weak or no fluorescence, with the emission quantum yields of 0.022 and <0.005, respectively. The fluorescence signal was significantly quenched in the presence of water, rendering no signal detectable for any of the studied complexes in H_2_O/1% DMF. Moreover, a negligible but measurable signal was observed for complexes **1** and **2** when excited at 430 nm (ϕ_em_ < 0.005 for both compounds), a region where these two complexes exhibit absorption (Figure 3B, bottom panel).

The effect of irradiation on the stability of the complexes was assessed by monitoring their UV–vis spectra. As shown in Figure 4A,B, an intense decrease in absorption was observed for **1** and **2** after irradiation with blue light (λ_max_ = 420 nm), indicating that irradiation facilitates structural changes in the complexes. In contrast, no spectral changes were observed for the nonirradiated solution of the complexes (Figure 4, panels D and E). Notably, no irradiation effects were observed for **3** (Figure 4, panels C and F), consistent with the absence of photopotentiation in cells. This likely implies that changes in structures of **1** and **2** induced by irradiation may be related to the enhancement of their biological activity. Notably, the spectral changes associated with a change in the composition of the studied compounds are different for complexes **1** and **2** in various mixtures of DMF and water. Specifically, the extent of the changes is greater for both complexes as the proportion of water increases in the given mixture with DMF. No relevant changes of UV–vis spectra were observed for the photostable complex **3** in different DMF:water mixtures (see Figure S17).

**Figure 4:**
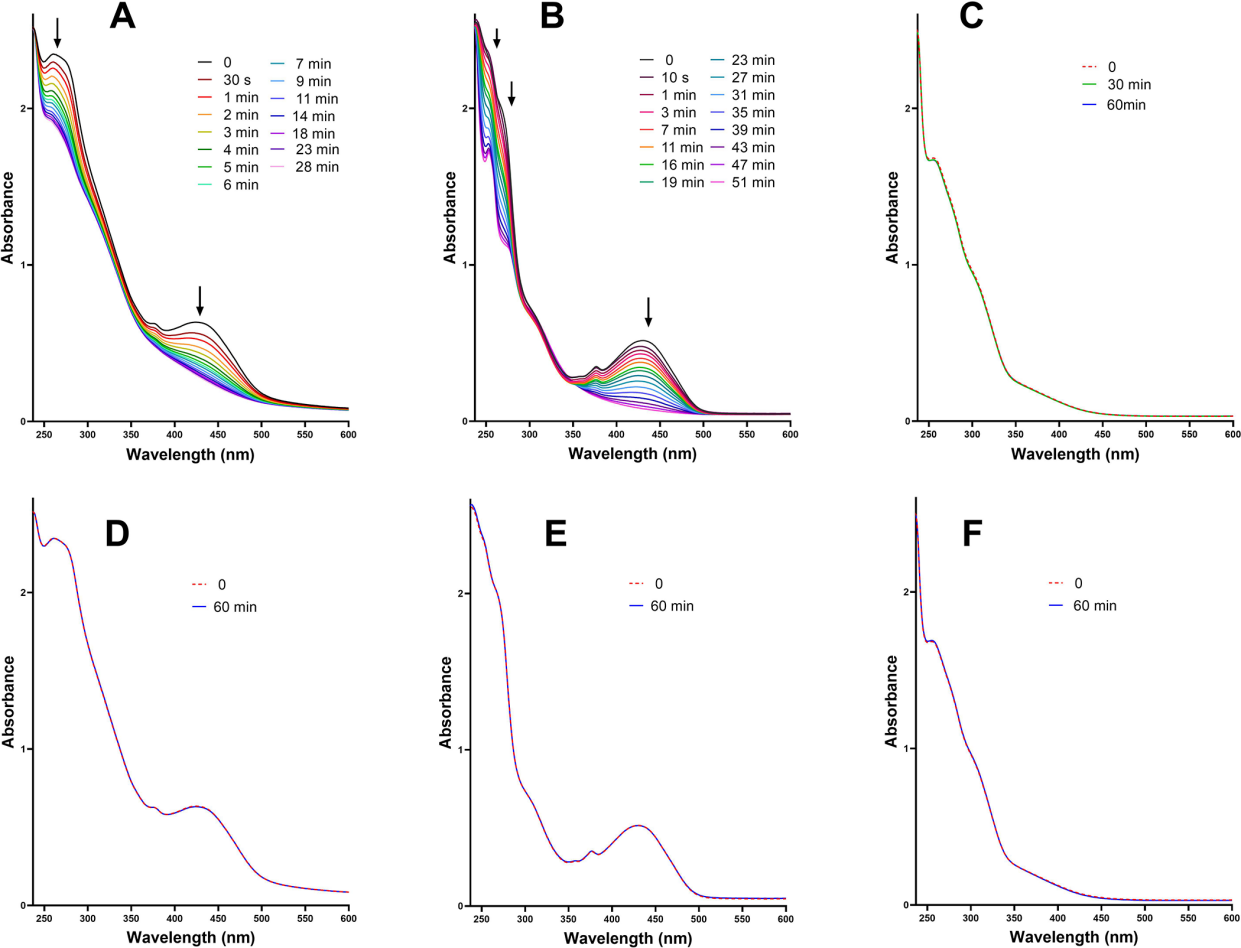
UV/vis absorption spectra of complex **1** (A, D), **2** (B, E), and **3** (C, F), 50 μM in H_2_O (1% DMF), upon blue light irradiation (5 mW cm^–2^) (panels A, B, and C) or dark incubation (panels D, E, and F) for the indicated time.

To better understand the effect of blue light on the structure of the complexes, mass spectra of the irradiated complexes were recorded for **1**–**3** (50 μM final concentration, 1% DMF in H_2_O with PBS) (Figures S18–S20). The changes detected by UV–vis for irradiated **1** (Figure 4A) are connected with the cleavage of the −N=CH– Schiff bond, as indicated by ESI-MS (Figure S18). Specifically, the mass spectrum of **1** (without irradiation) contains two dominant peaks at 837.0 *m*/*z* (for [RhCl­(Cp^bph^)­(L1)]^+^ and 598.2 *m*/*z* {[Rh­(bpt)­(Cp^bph^)] – H}^+^, showing that the N–N bond between triazole and aliphatic nitrogen cleaves under the electrospray ionization conditions used (Figure S18). After irradiation with blue light, new peaks were detected in the mass spectra of **1**, assignable to [Rh­(abpt)­Cl­(Cp^bph^)]^+^ (649.0 *m*/*z*) and its dechlorinated form {[Rh­(abpt)­(Cp^bph^)] – H}^+^ (613.2 *m*/*z*). This observation indicated that complex **1** is photocleaved to **3**, and free AC is achieved by a photocleavage of the – N=CH–Schiff bond of L1.

HPLC experiments were also performed on **1** under the same experimental conditions (50 μM final concentration of complexes, 1% DMF/99% PBS in water), confirming the mentioned cleavage of **1** to **3** + AC. Specifically, **1** showed at *t*_R_ = 17.55 min (MS: 837.0 *m*/*z* for [RhCl­(Cp^bph^)­(L1)]^+^), and the intensity of this peak decreased after irradiation (Figure S21). In connection, the second peak, mentioned above as detected by ESI-MS experiments (i.e., [Rh­(abpt)­Cl­(C^bph^)]^+^, 649.0 *m*/*z*), was observed at *t*_R_ = 9.85 min. These two peaks were supplemented by the signals detected at *t*_R_ = 8.13 and 14.22 min. The latter one can be assigned to the {[RhCl­(Cp^bph^)­(L1)] + O_2_}^+^ species (MS: 869.2 *m*/*z*), suggesting an oxidation of **1**. This could be connected with a known process of photooxygenation of the anthracene substituent of **1** to its endoperoxide form, as reported in the literature for free and derived anthracene.^17,18^ In connection with photooxygenation of the anthracene substituent, the signal detected at *t*_R_ = 8.13 min likely belongs to the released oxidized anthracene-9-carbaldehyde (endoperoxide), as proved by the control experiment with AC under the same experimental conditions, including irradiation by the blue light. Specifically, AC itself showed at 9.92 min, and a new peak was detected (*t*_R_ = 8.10 min) after AC was irradiated (Figure S22). Thus, the peak assigned above to the released complex **3** (*t*_R_ = 9.85 min) seems to involve both AC and **3**. The formation of the oxidized {[RhCl­(Cp^bph^)­(L1)] + O_2_}^+^ species was confirmed by analogous experiments with different mobile phases containing 0.1% tfa instead of ammonium formate.

Similarly to **1**, the second L1-containing complex, **2**, which is also unstable under irradiation (Figure 4B), undergoes a similar fragmentation achieved by a Schiff bond photocleavage, as confirmed by ESI-MS (Figure S19). Specifically, the signals assignable to the [Rh­(abpt)­Cl­(Cp*)]^+^ (511.0 *m*/*z*) and {[Rh­(abpt)­(Cp*)] – H}^+^ (475.1 *m*/*z*) entities were detected by ESI-MS in irradiated samples. According to the UV–vis and ESI-MS results, complex **2** is photocleaved in the same way (i.e., Schiff bond cleavage) as **1**, leading to the release of the [Rh­(abpt)­Cl­(Cp*)]^+^ species and free AC. On the other hand, no evidence of analogical photooxidation (photooxygenation), as discussed above for **1**, was obtained by similar HPLC experiments performed on complex **2.**

In contrast, complex **3** remains stable after the same irradiation, as indicated by the results of mass spectrometry (Figure S20), which is consistent with the UV–vis results (Figure 4C).

### Photophysical Properties in the Presence of NAD­(H) Coenzymes

Rh­(III) half-sandwich complexes have been extensively explored for their efficiency as catalysts in various organic transformations in cell-free environments and living cells. Rh-based catalysts hold potential as therapeutic agents because they can convert multiple substrate molecules while requiring low concentrations to achieve the desired activity. Numerous studies have reported an association of the catalytic activity of Rh half-sandwich complexes with their antitumor properties. The relationship between the catalytic properties of specific Rh­(III) half-sandwich complexes and their biological activity has been demonstrated through transfer hydrogenation catalysis of naturally occurring substrates.^19–24^ Various complexes of the type [Rh­(η^5^-Cp^*x*^)­Cl­(L)]^*n*+^ have been shown to catalyze the reduction of NAD^+^ to NADH and the conversion of pyruvate to lactate, both utilizing formate as a hydride donor and thus inducing reductive stress in cells. Structurally related complexes were also found to catalyze NADH oxidation and promote ROS formation in treated cancer cells.^25^ Based on these findings, we focused on studying the ability of complexes **1**–**3** to facilitate redox processes on NAD­(H) coenzymes through transfer hydrogenation.

Complexes **1**–**3** (50 μM final concentration) were studied by UV–vis spectroscopy for their ability to oxidize NADH (5 molar equiv) or to reduce NAD^+^ (5 molar equiv. mixed with 25 molar equiv of sodium formate) in 1% DMF/99% PBS in water mixture of solvents. Additionally, as different stabilities of L1-containing photocytotoxic complexes **1** and **2** were observed in the dark and upon irradiation, it was interesting for us to investigate the effect of the light used on the redox properties of these Rh­(III) complexes in relation to the coenzymes NAD^+^ and NADH. For these experiments, UV–vis spectra were recorded on solutions of complexes mixed with NADH (5 mol equiv) or NAD^+^ and sodium formate (5 and 25 mol equiv, respectively) after 10 min irradiation (blue light). Interestingly, NADH was rapidly oxidized to NAD^+^ in the presence of irradiated complexes **1** and **2** (Figure S23). In particular, the photopotentiation effect of the blue light on L1-containing complexes **1** and **2** led to the oxidation of ca. 22 and 28% NADH, respectively (after 10 min irradiation). Similar photopotentiation on NADH oxidation to NAD^+^ was recently reported for half-sandwich Ir­(III) complexes derived from the photosensitive 1-azaanthracene derivatives.^10^

The obtained results indicated a photocatalytic effect, as more than 1.0 mol equiv of NADH was oxidized to NAD^+^ by the photosensitive Rh­(III) complexes **1** and **2**. Control experiments with nonirradiated samples showed that the extent of NADH-to-NAD^+^ oxidation was even higher after 10 min of irradiation than after 24 h of standing in the dark. A lower effect (ca. 4% after 10 min irradiation) was observed for irradiated complex **3** (Figure S23).

The quantification mentioned above, derived from the UV–vis results, is only approximate because it is affected by the interference of the absorbance of NADH and the studied complexes at 340 nm. For this reason, a ^1^H NMR study was also performed for the complexes under identical conditions (i.e., 50 μM final concentration of complexes; 5 mol equiv of NADH; 1% DMF-*d*_7_). The results proved that NADH was oxidized to NAD^+^ in the presence of **1**–**3**. The formation of NAD^+^ is evidenced by its characteristic peaks at ca. 9.31, 9.13, and 8.83 ppm (Figures S24–S26). On the other hand, the distinctive resonance of unconverted NADH (δ = 6.92 ppm) remained present in the ^1^H NMR spectrum of the irradiated sample, which confirmed its integrity under the experimental conditions used. Complexes **1** and **2** exhibit a significant ability to oxidize NADH after irradiation (note: this is in line with other photochemical experiments, where we verified the ability of the complexes to oxidize NADH after 10 min of irradiation). Complex **2** was more active (TON = 4.2; TOF = 25.2 h^–1^) than **1** (TON = 1.6; TOF = 9.6 h^–1^) at *t* = 10 min. Complex **3** oxidized NADH to a lesser extent (TON = 0.5; TOF = 2.7 h^–1^).

Interestingly, **2** oxidized all the inserted NADH to NAD^+^ after 20 min of irradiation. This observation led us to perform similar experiments with higher NADH concentrations (250 molar equiv) and longer irradiation times of up to 60 min (reflecting the irradiation times within the phototoxicity experiments). Even under these experimental conditions, it was **2** that showed higher potency to oxidize NADH at *t* = 10 min (TON = 4.5; TOF = 27.2 h^–1^) than **1** (TON = 3.3; TOF = 20.0 h^–1^) (Figures S27 and S28). However, this difference was not observed at *t* = 60 min, as the obtained TON and TOF values were almost identical for **1** (TON = 7.3; TOF = 7.3 h^–1^) and **2** (TON = 7.4; TOF = 7.4 h^–1^).

It should be noted that the ability to oxidize NADH (or to reduce NAD^+^) is directly related to the induction of ROS and is thus generally accepted as the cause of the redox mechanism of the biological effect of half-sandwich complexes.^20,26^ On the other hand, many studies have discussed structurally similar compounds where the ability to oxidize NADH was not proven for biologically active substances, or where biological activity and NADH oxidation did not correlate for various compounds.^21,25,27^

Although the UV–vis spectra differ for irradiated and nonirradiated mixtures of **1** or **2** with excess NAD^+^ (with formate as a hydride source), these spectral changes are connected only with the decomposition of the L1-containing complexes (**1**, **2**), as the spectral changes (Figure S23) correspond to those observed for complexes in the absence of NAD^+^ (see above). In other words, no changes assignable to the NAD^+^ reduction to NADH were observed in the UV–vis spectra recorded on the mixtures of the complexes with NAD^+^ (and excess formate as a hydride source). We also qualitatively demonstrated the lack of NAD^+^ reduction using ^1^H NMR experiments, which did not show the characteristic NADH signal at approximately 7.22 ppm after 10 min of irradiation with blue light (Figures S29–S31). These results suggest that **1**–**3** are not able to transfer hydrogen to the NAD^+^ coenzyme. In this context, it has to be noted that the ability to reduce NAD^+^ by half-sandwich Rh­(III) complexes did not correlate with their antiproliferative activity.^21^

### Mechanism of Phototoxic Action

The results of the mass spectrometric analysis described above demonstrated that irradiation of **1** with blue light leads to the formation of complex **3** and the release of free anthracene-9-carbaldehyde (AC). Anthracene and its derivatives are recognized as bioactive compounds due to their diverse biological activities, which exhibit effects in various cell types, including cancer cells.^28^ Anthracene bearing an aldehyde group or its metabolic product, anthracene carboxylic acid, can act through multiple mechanisms, such as intercalation into DNA^29^ or binding to proteins.^30^ Additionally, its aldehyde group can form Schiff bases with amines, potentially disrupting various biochemical processes.^31^ Moreover, anthracene has been identified as a photodynamically active compound capable of generating singlet oxygen and participating in both Type II and Type I photoreactions.^32^ It is therefore plausible that upon irradiation and the release of bioactive anthracene-9-aldehyde, this compound could contribute to the observed photopotentiation of **1** (and also **2**) through its own biological activity. To test this hypothesis, we examined the activity of free AC as well as its 1:1 mixture with complex **3**, which simulates the intracellular situation following irradiation after treatment with complex **1**. Data are summarized in Table 3.

**Table 3:** IC_50_ Values (μM) Obtained for A375 Cells Treated with Anthracene-9-carbaldehyde (AC), 3, or a Mixture of the Two for 1 h and Irradiated by Blue Light (1 h, Λ_max_ = 420 nm, and 50 ± 2 W m^–2^) or Sham-Irradiated, as Determined by the MTT Assay 70 h after Irradiation

	IC_50_ ^a^, [μM]	
	420 nm	dark
AC	0.9 ± 0.1	>100
MIX (**3** + AC, 1:1)	1 ± 0.2	36 ± 5
**3**	33 ± 2	34 ± 3

a Data represent mean ± SD from two independent experiments, each performed in duplicate.

In the dark, AC exhibited very low activity against melanoma cells. Due to this low activity, its presence in a mixture with complex **3** does not influence the overall activity, which appears to be primarily determined by complex **3.** In contrast, upon irradiation with blue light, AC exhibits a high degree of activation, rendering its effect decisive when combined with complex **3**. These results indicate that the release and action of AC significantly contribute to the phototoxicity of complexes **1** and **2.**

It should be noted that although the photoactivity of AC, either alone or in combination with complex **3**, is high, it is approximately five times lower than that of complexes **1** and **2** (Tables 2 and 3). This suggests that the attachment of the anthracene group to the Rh moiety provides added value that cannot be achieved by administering a simple mixture of the two components. One possible explanation is that when both components are administered separately, they may cross the membrane with different kinetics, leading to a different intracellular ratio compared to when both components pass through the membrane together as part of a single molecule and are only released upon irradiation. Moreover, the molar absorption coefficient of AC at 420 nm is significantly lower than that of **1** and **2**, which may also contribute to the weaker photoactivity of AC.

When irradiated, the A375 cells pretreated with **1** adopted a distinct morphology, markedly different from that of untreated irradiated cells. The most prominent characteristic was the extensive cytoplasmic vacuolization, where vacuoles occupied nearly the entire cytoplasm, accompanied by the formation of organelle-free bulges or blisters. The cells appeared swollen and rounded (Figure 5A). In the early stages of this process, pronounced cytoplasmic blebbing was evident (Figure S15). Notably, the bubbles surrounding the cells were inside completely clear, distinguishing them from the budding typically associated with apoptosis. These morphological features, widely recognized as typical of oncosis,^33,34^ suggest that **1** and **2** induce an oncosis-like form of cell death when irradiated.

**Figure 5:**
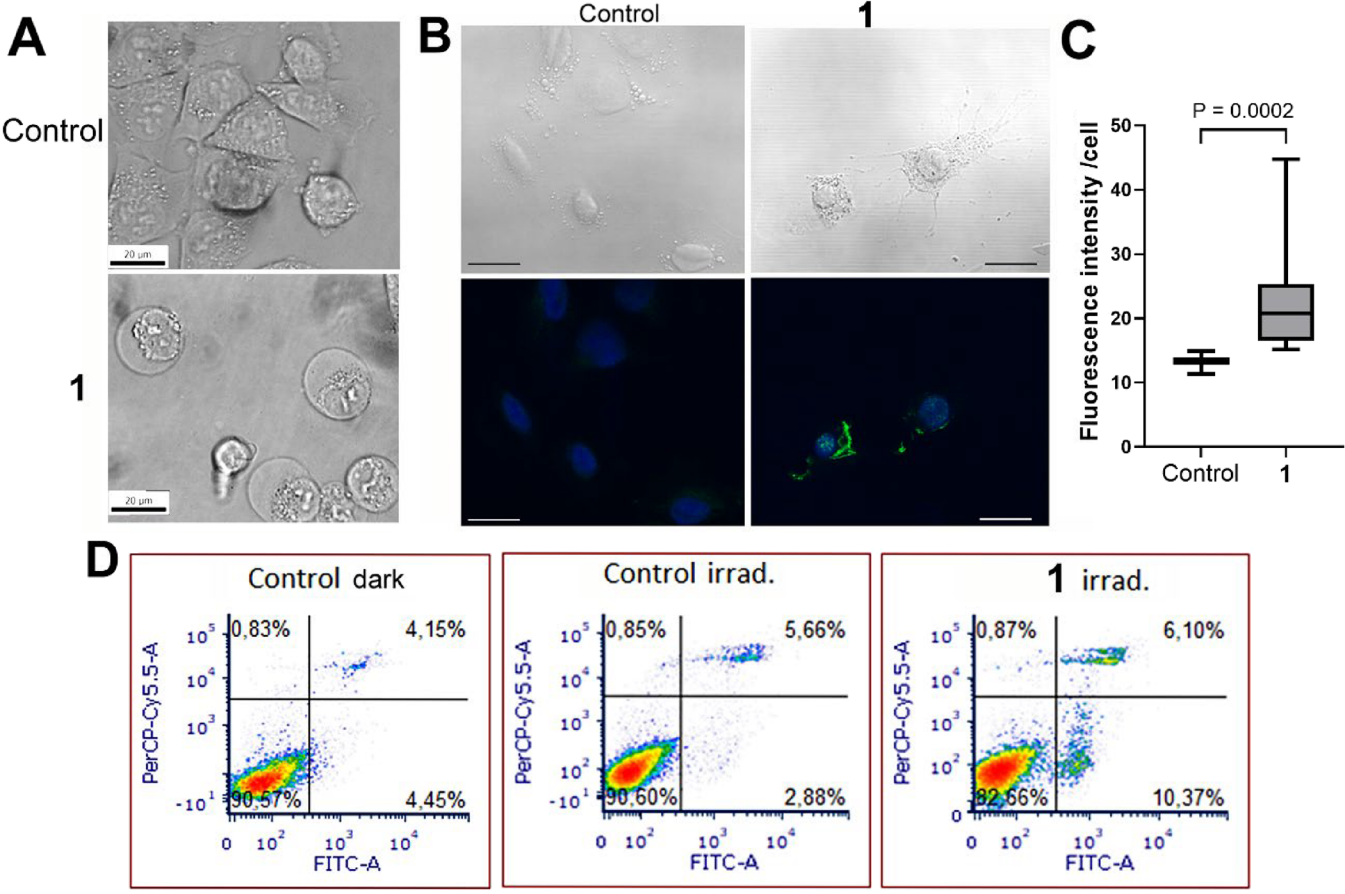
(A) Microscopic images of A375 cell morphology. Vacuolization of cytoplasm and cell swelling, as appears in an inverted optical microscope. Cells were treated with **1** (0.5 μM) or left untreated for 1 h, irradiated for 1 h with blue light, and then further incubated in complex-free media for 30 min in the dark. Scale bars = 20 μm. (B) Immunofluorescence staining of porimin in A375 cells, untreated or treated with **1** (0.5 μM), irradiated with blue light, and further incubated with Rh-free media for 4 h in the dark. Top panels, bright field; bottom panels, merged signals from blue and green fluorescence channels. Porimin appears as a green fluorescence signal; the nuclei of the cells are stained with DAPI (blue). Scale bars = 15 μm. (C) Quantitative evaluation of fluorescence intensity in cells immunolabeled for porimin. At least 25 cells in four different fields were included in the evaluation. Statistical significance was determined using the Mann–Whitney test. (D) Representative density plots of cells after their PI/annexin V FITC staining. Cells were incubated with **1** (0.2 μM) for 1 h, irradiated with blue light for 1 h, and immediately stained with PI/annexin. For detailed experimental procedures, see the Experimental Section.

To further support the conclusion based on cell morphology, surface receptor porimin was examined using a specific antibody. This protein plays a crucial role in increasing membrane permeability, leading to the characteristic whole-cell swelling observed during oncosis. The activation of porimin is widely recognized as an indicator of oncotic cell death.^35^ As shown in Figure 5B, elevated expression of porimin was demonstrated by immunostaining upon incubation with **1** and subsequent irradiation. Moreover, it has been shown that in oncosis, annexin V is also externalized during the early phase of oncosis.^15,35^ This phenomenon was also confirmed for cells treated with **1** under irradiation (Figure 5C). Similar indications of oncotic-like cell death mechanism were also obtained for complex **2** (Figure S32).

Thus, an increase in fluorescence associated with elevated porimin expression, together with annexin positivity and propidium iodide negativity in cells at an early stage of cellular response, supports oncosis-like death as the prevailing mechanism of cellular response induced by **1** (and **2**) under irradiation.

### ROS Production and Oxidative Stress

Previous studies have reported that oncosis is associated with reactive oxygen species (ROS) and oxidative stress.^35–37^ Further experiments were therefore aimed at determining whether oxidative stress occurs in treated cells due to irradiation. To detect intracellular ROS in live cells, the cell-permeant fluorogenic probe CellROX Green Reagent was used. The dye is weakly fluorescent in its reduced state and exhibits bright, photostable green fluorescence upon oxidation by ROS. After irradiation, the intracellular ROS level was significantly elevated in cells treated with **1** (Figure 6) and also with **2** (Figure S33); however, no significant ROS production was detected in cells treated with **3** or in the untreated control, despite irradiation of the cells (Figures 6 and S33). Importantly, if the cells were kept in the dark (sham-irradiated), no significant fluorescence signal corresponding to oxidized CellRox Green probe was detected after treatment with any of the three investigated Rh-complexes, indicating that the production of ROS is photoinducible (Figures 6 and S33). The results of this experiment correlate with the data on phototoxicity (Table 2), suggesting that the photoactivity of the **1** (and **2**) likely results, at least partially, from the intracellular ROS generation.

**Figure 6:**
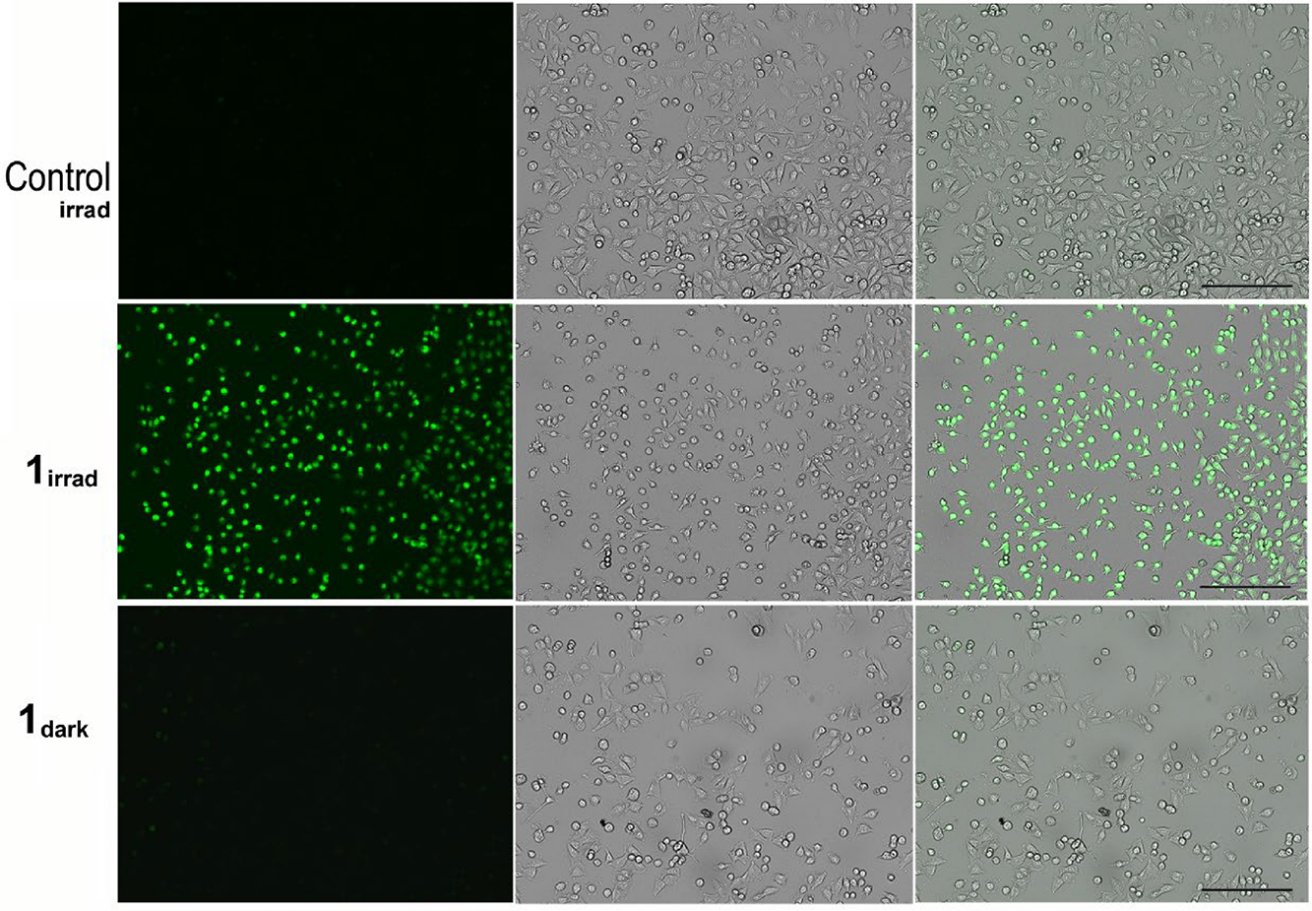
Detection of photoinduced ROS generation in A375 cells. Cells were untreated (control) or treated with **1** (0.5 μM) for 1 h in the dark and then irradiated with blue light for 1 h. For the detailed experimental procedure, see the Experimental Section. Left panels: green fluorescence channel; central panels: bright field; and right panels: overlay of the bright field and fluorescence channels. Scale bars = 200 μm.

To distinguish whether ROS originate from the complexes themselves or are generated in cells as a biological response to treatment and irradiation (e.g., due to damage and depolarization of mitochondrial membranes, etc.), the formation of photoinduced ROS was assessed in a cell-free environment. For this purpose, dihydrorhodamine 123 (DHR 123), a commonly used ROS tracking agent,^38^ was employed. When oxidized by ROS, the nonfluorescent DHR 123 converts into the fluorescent rhodamine 123, which emits at ∼530 nm. Under irradiation conditions, DHR 123 was oxidized by emerging ROS, as demonstrated by the increase in fluorescence emission (Figure 7A). It was verified that the intrinsic fluorescence of **1** does not affect the resulting signal (Figure 7A, dashed lines). Previously, it has been shown^39^ that irradiation (particularly UV) increases the green fluorescence of DHR 123. To assess the effect of blue light on the DHR 123 probe and avoid false-positive results, we also irradiated DHR 123 alone under the same conditions, but in the absence of **1**. The results also showed an increase in fluorescence intensity (Figure 7B), but to a significantly lesser extent (ca. 3-fold) than in the presence of **1** (see also Figure S34). This suggests that although radiation itself may contribute to the oxidation of DHR 123 in solution, a substantial portion of the fluorescence increase can be attributed to oxidation by ROS generated by complex **1.**

**Figure 7:**
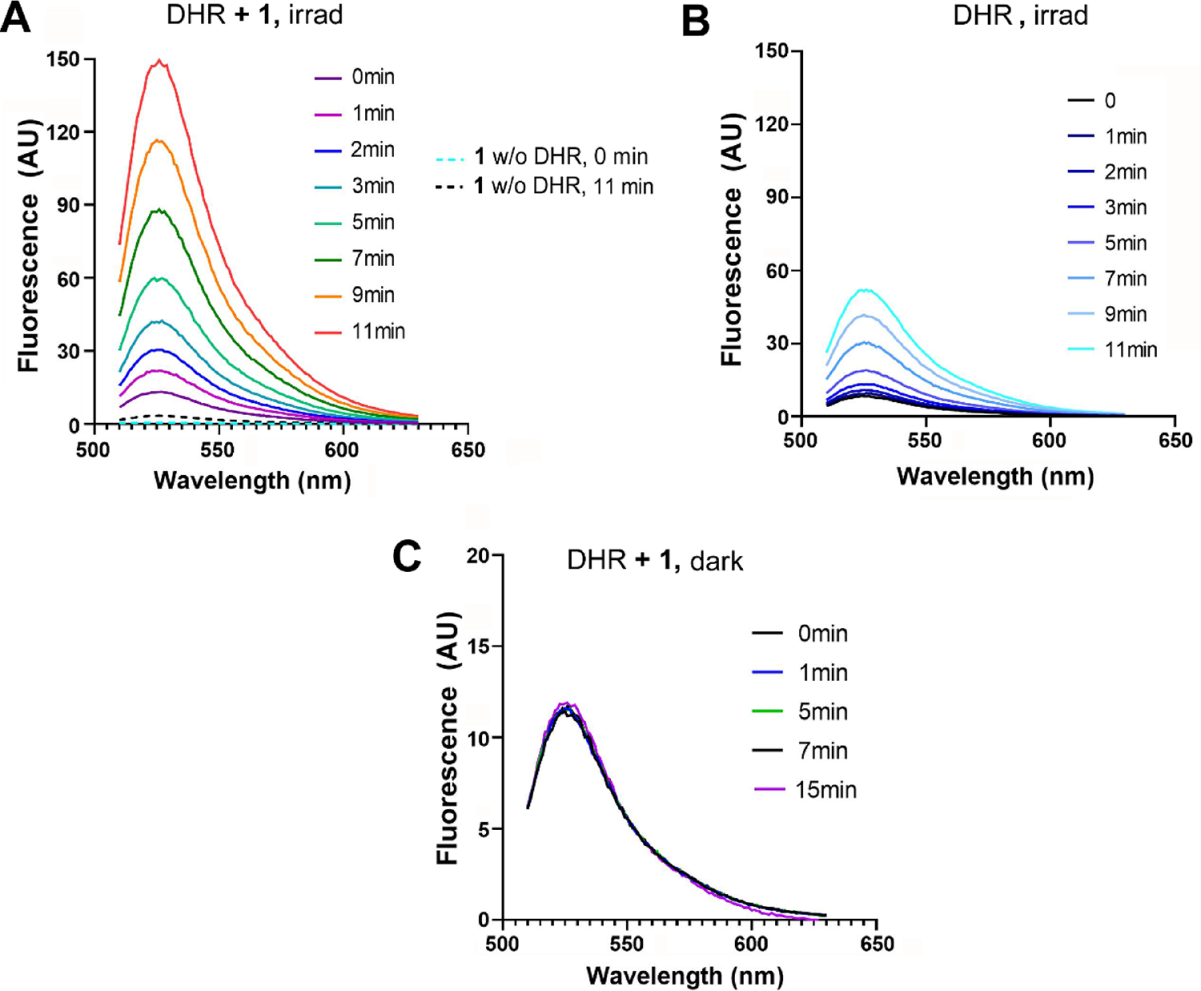
Emission spectra of DHR 123. (A) 12.5 μM DHR 123 + **1** (10 μM) in PBS, irradiated with blue light (λ = 420 nm) for the indicated intervals. (B) 12.5 μM DHR 123 in PBS, irradiated with blue light (λ = 420 mn and 5 mW cm^–2^) for indicated intervals. (C) 12.5 μM DHR 123 + **1** (10 μM), incubated nonirradiated for indicated intervals.

Notably, there was no change in fluorescence when DHR 123 was incubated with **1** under the same conditions without irradiation (Figure 7C). Thus, the results are in agreement with experiments on cell lines, where an increase in oxidative stress was also observed after irradiation but not in the dark. Additionally, the results demonstrate that ROS can be generated due to the interaction of light radiation with **1**, even in a cell-free environment.

The above-described results demonstrate that photopotentiation of **1** (and **2**) is related to ROS formation, and that the resulting oxidative stress significantly contributes to their photocytotoxicity. Further experiments aimed to determine the type of reactive species generated by irradiation of **1**. Although half-sandwich Rh-complexes have been shown to generate ^1^O_2_,^12,40^ other cytotoxic ROS can also be generated through type I photochemical reactions.

To determine the nature of ROS generated after treatment with **1**, its phototoxicity was assessed in A375 cells in the presence of selective ROS scavengers as described in the Experimental Section. It was verified that the individual scavengers at the concentrations used did not affect the cell viability compared to the control incubated in the complex-free medium.^16^

The effects of various ROS inhibitors and scavengers on the phototoxic effect of **1** after the irradiation are summarized in Figure 8. The effect of irradiated **1** was not inhibited in the presence of hydroxyl radical (OH·) and hydrogen peroxide (H_2_O_2_) scavengers (mannitol, and sodium pyruvate, respectively), even at high concentrations (50 and 10 mM, respectively). This suggests that neither hydroxyl radicals nor hydrogen peroxide significantly contributes to the phototoxicity of **1**. In contrast, the presence of sodium azide, a potent singlet oxygen quencher, weakened the phototoxic effect of **1**, suggesting that singlet oxygen is likely a candidate responsible for intracellular oxidative stress induced by **1.** Moreover, the presence of the cell-permeable superoxide scavenger tiron also resulted in a decrease in the phototoxicity of **1**, suggesting that superoxide anions (·O_2_^–^) may also contribute to the phototoxic activity of **1.** Similar data have also been obtained for complex **2** (Figure S35).

**Figure 8:**
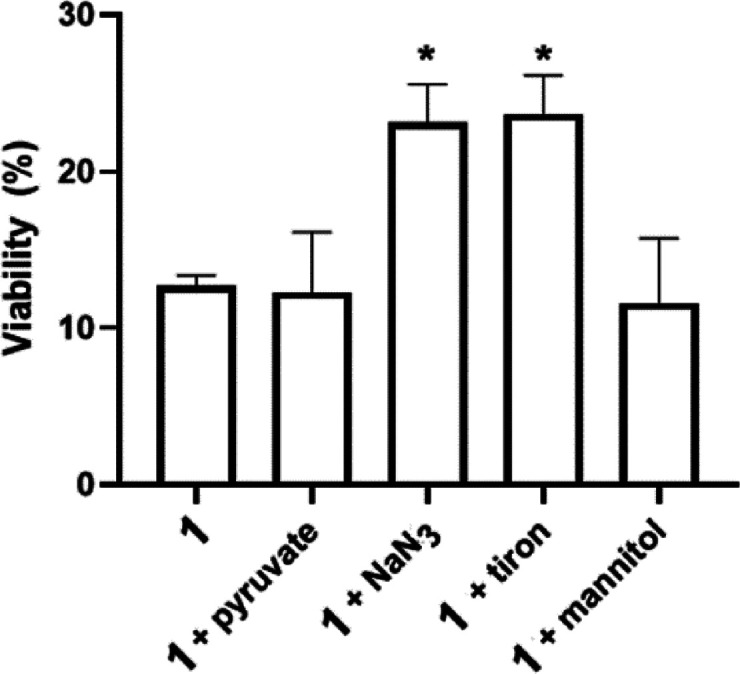
Viability of A375 cells treated and irradiated with **1** (0.5 μM) in the presence of ROS scavengers, such as sodium pyruvate (10 mM), sodium azide (5 mM), tiron (5 mM), and D-mannitol (50 mM). Viabilities of cells irradiated in the medium with the respective scavenger but without complex **1** were taken as 100%. * = significantly different (*p* < 0.05) from viability of cells treated and irradiated in the absence of any scavenger as determined by the Mann–Whitney nonparametric test, *n* = 4.

### Effect on 3D Cell Cultures

Given that three-dimensional (3D) cell culture systems provide a more physiologically relevant model for *in vitro* evaluation of anticancer agents,^41–43^ the effects of **1** and **2** were also examined in a 3D culture. Unfortunately, the A375 cell line forms 3D spheroids with difficulty, and those that do form are irregular in shape, fragile, and challenging to handle.^44^ Therefore, the human colon cancer cell line HCT116 was selected for this experiment.

First, the ability of **1** and **2** to penetrate the cell mass of the spheroid was determined using confocal microscopy. The tumor spheres from HCT116 (diameter: 220–230 μm) were treated with **1** or **2** for 5 h, and the resulting fluorescence signal was visualized by confocal microscopy in defined z-stack steps (Figure S36). The intensity of the fluorescence signal was plotted (Figure S36E). As indicated, the fluorescence signal was distributed throughout the entire cell mass, including deep-seated sections. This observation suggests that **1** and **2** can target not only cells in the periphery but also quiescent cells in the intermediate zone, as well as the necrotic core of the spheroid.

To assess the phototoxic effect of Rh-complexes, HCT116 cells were seeded into 96-well, U-bottom, ultralow attachment plates and incubated in 3D culture-supporting medium for 72 h to allow spheroid formation. Subsequently, the spheroids were exposed to either **1** or **2** for 5 h, rinsed, transferred into confocal imaging dishes, and subjected to 405 nm laser irradiation for 5 min (final output power 1 mW) or kept in the dark. Following this treatment, the spheroids were cultured for an additional 24 h and stained with calcein AM (a live-cell-permeable dye) and propidium iodide (PI, which labels cells with compromised membrane integrity). Confocal microscopy was performed using z-stack scanning (10 layers per sample), and PI and calcein fluorescence were quantified to assess the proportion of dead and live cells, respectively, within each spheroid. Moreover, Hoechst staining was applied to all samples to delineate spheroid contours for accurate analysis.^45,46^

Representative images are shown in Figure 9. As indicated, irradiation of the spheroid pretreated with **1** or **2** increased PI fluorescence, indicating a higher extent of cell death within the spheroid mass. The increase in the PI/calcein fluorescence ratio represents 28 or 42% for spheroids treated with **1** or **2**, respectively, compared to the untreated irradiated control (Figure S37). In contrast, nonirradiated samples exhibited minimal changes in PI fluorescence.

**Figure 9:**
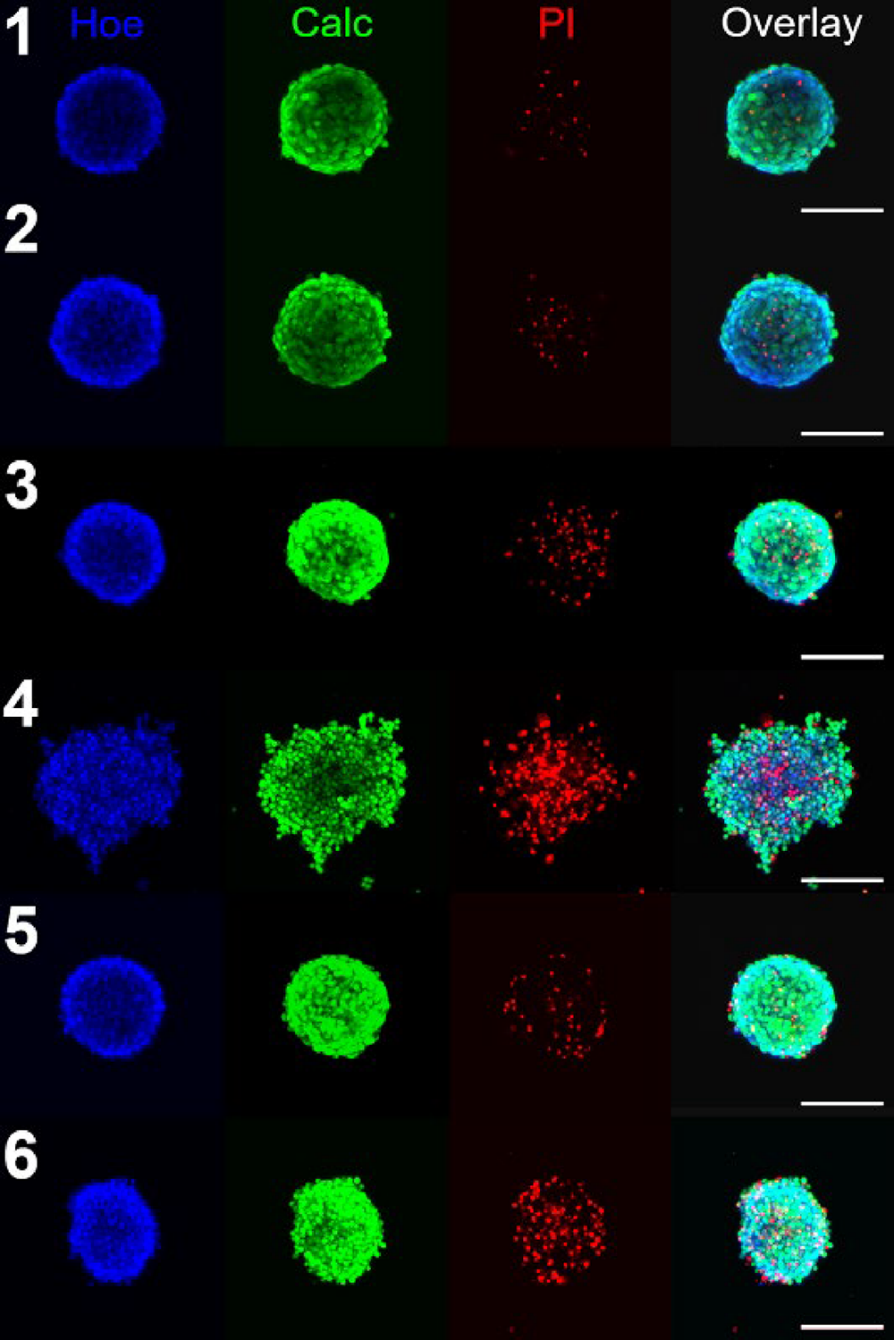
Analysis of the HCT116 spheroids on confocal microscopy. Shown in sequence from left to right, spheroids were stained with Hoechst 33258 dye, calcein AM, and propidium iodide. Merged channels are shown in the rightmost panels. Samples: (1 and 2) untreated control, (3 and 4) cells treated with **2** (6 μM), and (5 and 6) cells treated with **1** (6 μM). Samples in panels 1, 3, and 5 were kept in the dark; samples 2, 4, and 6 were irradiated for 5 min with blue (405 nm) laser light 24 h before analysis. Pictures show a representative spheroid from two independent experiments. The scale bar represents 200 μm.

The morphology of the spheroid was also significantly affected by irradiation following treatment with either **1** or **2**. When irradiated, both **1** and **2** caused damage to the spheroids, as evidenced by the disintegration of the spheroids and the accumulation of cell debris, particularly in the case of **2** (Figures 9 and S38). Complex **1** caused a size reduction of the spheroid compared to the control, untreated colonosphere.

These findings demonstrate that the investigated Rh complexes can effectively induce cytotoxic effects in 3D tumor spheroids. However, the activity is observed at higher concentrations compared to that seen in two-dimensional (2D) monolayer cultures. This likely reflects the specific features of 3D systems, such as limited light penetration into the spheroid core or the hypoxic environment in the inner layers of the spheroid, which limits the formation of ROS. Nevertheless, these results clearly show that the complexes exhibit plausible phototoxicity even in a 3D cell model when applied at micromolar concentrations.

## Conclusions

In this study, we successfully designed and synthesized a half-sandwich Rh­(III) complex [Rh­(η^5^-Cp^bph^)­Cl­(L1)]­PF_6_ incorporating biphenyl and anthracene-9-carbaldehyde (AC) photosensitive substituents (complex **1**). To assess the role of both substituents (biphenyl of Cp^bph^ and AC of L1) in the biological activity of this complex, its less substituted analogs (complexes **2** and **3**) were also prepared and examined. UV–vis, ESI-MS, and ^1^H NMR spectroscopy confirmed the structural stability of these complexes in DMF/PBS solutions. All three complexes exhibited moderate cytotoxicity in the dark against human breast, colorectal, and ovarian cancer cell lines, with IC_5__0_ values in the micromolar range. Notably, complexes **1** (bearing both anthracene and biphenyl substituents) and, especially, complex **2** (bearing only an anthracene substituent) demonstrated pronounced phototoxicity upon light activation, particularly under blue light, with complex **2** achieving a phototoxicity index (PTI) of 144 in the A375 melanoma cell line.

In contrast, complex **3**, which lacks the anthracene moiety, showed no enhancement in cytotoxicity upon irradiation, consistent with its photostability as verified by UV–vis spectral analysis. Irradiation of complexes **1** and **2** with blue light resulted in photocleavage, releasing anthracene-9-carbaldehyde and either complex **3** (for **1**) or the [Rh­(abpt)­Cl­(Cp*)]^+^ (for **2**) fragment, respectively. UV–vis and mass spectrometry analyses confirmed that this photoreactivity occurs via cleavage of the −N=CH– bond, highlighting the essential role of the anthracene-derived Schiff base in light-induced activation. The presence of the biphenyl substituent did not contribute to photoactivation or enhance photocytotoxic effects.

To elucidate the underlying mechanism of phototoxicity, we investigated the photocatalytic redox activity of complexes **1** and **2**. Upon blue light irradiation, both complexes effectively catalyzed the oxidation of NADH to NAD^+^, as demonstrated by ^1^H NMR and UV–vis spectroscopy. However, these complexes did not facilitate the reverse hydrogenation of NAD^+^ to NADH in the presence of sodium formate, suggesting a unidirectional catalytic function. Mechanistic studies further revealed that light-induced cytotoxicity of complexes **1** and **2** predominantly involves oncosis-like cell death, mediated by elevated levels of reactive oxygen species (ROS) and oxidative stress.

These findings demonstrate that rational ligand design, specifically incorporating an anthracene moiety via a Schiff base linkage, is critical for achieving photoresponsive behavior in rhodium­(III) complexes. The high phototoxicity indices observed, particularly for complex **2**, represent some of the most potent reported for Rh­(III)-based photosensitizers to date. Furthermore, both complexes retained potent photocytotoxicity in 3D tumor spheroids, with complex **2** exhibiting superior tissue penetration. These properties underscore the potential of such rhodium complexes as promising candidates for further development in photodynamic cancer therapy, offering tumor-selective activation and improved therapeutic precision.

## Experimental Section

### Materials and General Methods

4-Bromo-biphenyl, 1.6 M *n*-BuLi/hexane solution, 2,3,4,5-tetramethyl-2-cyclopentenone, RhCl_3_·*n*H_2_O, pentamethylcyclopentadiene (HCp*), 3,5-di­(pyridin-2-yl)-4*H*-1,2,4-triazol-4-amine (abpt), HCl (conc.), acetic acid (conc.), anthracene-9-carbaldehyde (AC), NaCl, MgSO_4_, NH_4_PF_6_, silver­(I) triflate, phosphate-buffered saline (PBS; powder), β-nicotinamide adenine dinucleotide-reduced disodium salt hydrate (NADH), β-nicotinamide adenine dinucleotide hydrate (NAD^+^), sodium formate and solvents of a laboratory (methanol (MeOH), acetonitrile (ACN), *n*-hexane, diethyl ether (DEE), ethyl acetate) and HPLC (water, ACN) grades were purchased from Merck Life Science (Prague, Czech Republic), VWR International (Stříbrná Skalice, Czech Republic) or Lach-Ner (Neratovice, Czech Republic). The chemicals and solvents were used as received without further purification. Solvents for NMR experiments (DMSO-*d*_*6*_, DMF-*d*_7_ *,* and D_2_O) were supplied by Chemstar (Plzeň, Czech Republic).

Compound 4-(2,3,4,5-tetramethylcyclopenta-2,4-dien-1-yl)­biphenyl (HCp^bph^) was synthesized as previously reported.^47^ The starting dimeric Rh­(III) complexes [Rh­(μ-Cl)­(η^5^-Cp*)­Cl]_2_ and [Rh­(μ-Cl)­(η^5^-Cp^bph^)­Cl]_2_ were synthesized in a Monowave 300 microwave reaction system (Anton Paar GmbH) using an updated procedure^48^ of the original synthesis.^21,49,50^

All compounds, submitted for biological testing, are >95% pure by HPLC analysis (Figure S6).

Elemental analysis (C, H, N) was performed using a Flash 2000 CHNS (Thermo Scientific) analyzer. Electrospray ionization mass spectrometry (ESI-MS) was conducted using an LCQ Fleet ion trap mass spectrometer (Thermo Scientific) on solutions of the studied complexes in MeOH or AC. The data were obtained in both the positive (ESI+) and negative (ESI−) ionization modes, and they were analyzed by QualBrowser (version 2.0.7). Nuclear magnetic resonance (^1^H NMR) was performed using a Varian-400 spectrometer. The samples were prepared in DMSO-*d*_6_ *,* and the spectra were referenced to a signal of the solvent used (2.50 ppm). A Jasco FT/IR-4700 spectrometer was used to record the Fourier-transform infrared (FT-IR) spectra at 400–4000 cm^–1^ (attenuated total reflection (ATR) technique on a diamond plate). The FT-IR spectral intensity is defined as w = weak, m = middle, s = strong, and vs = very strong peak.

### Syntheses

#### N-[1-(Anthracene-9-yl)-methylidene]-3,5-di­(pyridin-2-yl)-4H-1,2,4-triazol-4-amine (L1)

AC (1 mmol) was mixed with abpt (1 mmol) and acetic acid (0.2 mL) in MeOH (10 mL), and the mixture was stirred at r.t. After 3 h, the yellow precipitate was removed, and compound L1 was obtained by slow evaporation of the yellow filtrate. The yellow solid of L1 was collected by filtration, washed with MeOH (3 × 5 mL) and DEE (2 × 2 mL). Yield: 64%. C_27_H_18_N_6_ (*M*_w_ = 426.47). Anal. Calc.: C, 76.0, H, 4.3, N, 19.7; found: C, 75.9, H, 4.2, N, 20.0%. ^1^H NMR (400 MHz, DMSO-*d*_6_, 298 K, ppm): δ 10.13 (s, 1H), 8.88 (s, 1H), 8.71 (d, *J* = 8.6 Hz, 2H), 8.61–8.60 (m, 2H), 8.25–8.17 (m, 4H), 8.08 (t, *J* = 7.8 Hz, 2H), 7.62–7.53 (m, 6H). IR (ATR, ν, cm^–1^): 3249w, 3173w, 3088m, 6041vs, 3023w, 3000w, 2938w, 2889w, 2859w, 2853w, 2764w, 2701w, 2634w, 2511w, 2451w, 2418w, 2398w, 2352w, 2326w, 2296w, 2187w, 2011m, 1950m, 1901m, 1855w 1830w, 1799m, 1765w, 1731w, 1714w, 1691w, 1624s, 1584vs, 1566w, 1517vs, 1490w, 1445vs, 1423vs, 1370m, 1338m, 1267s, 1253s, 1184s, 1159s, 1146vs, 1114w, 1092s, 1072s, 1034m, 1021m, 994vs, 967s, 949s, 880vs, 839vs, 807w, 788vs, 775s, 726vs, 688vs, 637m, 612vs, 596m, 558m, 536s, 491m.

### Coordination Compounds **1–3**

#### [Rh­(η^5^-Cp^bph^)­Cl­(L1)]­PF_6_ (**1**)

Complex **1** was prepared by reacting [Rh­(μ-Cl)­(η^5^-Cp^bph^)­Cl]_2_ (0.05 mmol, 0.045 g) with L1 (0.1 mmol, 0.042 g). The reactants were suspended in 3 mL of MeOH in a microwave vial, and the suspension was heated to 100 °C in the microwave reactor for 3 min. The obtained solution was cooled to the ambient temperature and filtered. To this solution, NH_4_PF_6_ (0.25 mmol, 0.040 g) was added under continuous stirring. The yellow precipitate of complex **1** was collected by centrifugation, washed with MeOH (3 × 2 mL) and DEE (1 × 2 mL), and dried at 45 °C.

For **1**: Yield: 73%. C_48_H_39_N_6_ClF_6_PRh (*M*_w_ = 983.2). Anal. Calc.: C, 58.6, H, 4.0, N, 8.5; found: C, 58.4, H, 3.8, N, 8.1%. ^1^H NMR (400 MHz, DMSO-*d*_6_, 298 K, ppm): δ 10.42 (s, 1H), 9.03 (s, 1H), 8.95 (d, *J* = 8.8 Hz, 2H), 8.73 (d, *J* = 5.4 Hz, 1H), 8.40–8.38 (m, 1H), 8.33–8.31 (m, 1H), 8.28–8.26 (m, 2H), 8.24–8.21 (m, 1H), 8.19–8.16 (dd, *J* = 7.8 Hz, *J* = 1.5 Hz, 1H), 8.15–8.10 (m, 1H), 7.89 (s, 3H), 7.82–7.78 (m, 3H), 7.72–7.64 (m, 4H), 7.55–7.49 (m, 3H), 7.46–7.42 (m, 2H), 1.99 (s, 3H), 1.91 (d, *J* = 3.4 Hz, 6H), 1.85 (s, 3H). ESI-MS (ACN, *m*/*z*): 801.1 (calc. 801.2; 10%; {[Rh­(Cp^bph^)­(L1)] – H}^+^), 837.2 (calc. 837.2; 100%; [RhCl­(Cp^bph^)­(L1)]^+^). IR (ATR, ν, cm^–1^): 3642w, 3052w, 3027w, 2915w, 2371w, 2348w, 2319w, 2015w, 1993w, 1747w, 1613m, 1586m, 1553m, 1519m, 1487m, 1448s, 1426w, 1378w, 1341m, 1285w, 1255m, 1233w, 1188w, 1159m, 1108w, 1095w, 1070w, 1043w, 1021m, 994w, 967w, 916w, 835vs, 788w, 766w, 739s, 695s, 652m, 612m, 592w, 556s.

#### [Rh­(η^5^-Cp*)­Cl­(L1)]­PF6 (**2**) and [Rh­(η^5^-Cp^bph^)­(abpt)­Cl]­PF_6_ (**3**)

Complexes [Rh­(η^5^-Cp*)­Cl­(L1)]­PF_6_ (**2**) and [Rh­(η^5^-Cp^bph^)­(abpt)­Cl]­PF_6_ (**3**) were prepared as described for **1**, using different reactants: [Rh­(μ-Cl)­(η^5^-Cp*)­Cl]_2_ and L1 for **2**, and [Rh­(μ-Cl)­(η^5^-Cp^bph^)­Cl]_2_ and abpt for **3.**

For **2**: Yield: 77%. C_37_H_33_N_6_ClF_6_PRh (*M*_w_ = 845.0). Anal. Calc.: C, 52.6, H, 3.9, N, 9.9; found: C, 52.3, H, 3.6, N, 9.6%. ^1^H NMR (400 MHz, DMSO-*d*_6_, 298 K, ppm): δ 10.38 (s, 1H), 9.09 (d, *J* = 5.4 Hz, 1H), 9.02 (s, 1H), 8.92 (d, *J* = 8.8 Hz, 2H), 8.44 (d, *J* = 7.8 Hz), 8.32–8.25 (m, 3H), 8.18–8.12 (m, 3H), 7.85 (m, 1H), 7.71–7.60 (m, 4H), 7.51–7.49 (m, 1H), 1.82 (s, 15H). ESI-MS (ACN, *m*/*z*): 663.0 (calc. 663.2; 30%; {[Rh­(Cp*)­(L1)] – H}^+^), 699.1 (calc. 699.2; 100%; [RhCl­(Cp*)­(L1)]^+^). IR (ATR, ν, cm^–1^): 3633w, 3115w, 3049w, 2965w, 2913w, 2344w, 1984w, 1611w, 1584m, 1553m, 1521w, 1485m, 1448s, 1426w, 1380w, 1341w, 1295w, 1282w, 1260m, 1995w, 1161m, 1148w, 1068w, 1021m, 994w, 973w, 912w, 833vs, 788w, 778w, 739s, 688m, 649m, 614m, 556s, 536w, 491w, 459w.

For **3**: Yield: 81%. C_33_H_31_N_6_ClF_6_PRh (*M*_w_ = 795.0). Anal. Calc.: C, 49.9, H, 3.9, N, 10.6; found: C, 49.6, H, 3.8, N, 10.3%. ^1^H NMR (400 MHz, DMSO-*d*_6_, 298 K, ppm): δ 8.88–8.86 (m, 2H), 8.72 (d, *J* = 5.4 Hz, 1H), 8.41–8.37 (td, *J* = 8.3 Hz, *J* = 1.5 Hz 1H), 8.31 (d, *J* = 7.8 Hz, 1H), 8.19–8.15 (td, *J* = 7.8 Hz, *J* = 2.0 Hz, 1H), 7.87–7.83 (m, 3H), 7.78 (d, *J* = 8.3 Hz, 4H), 7.74–7.71 (m, 1H), 7.52 (t, *J* = 7.34 Hz, 2H), 7.46–7.41 (m, 3H), 1.94 (s, 3H), 1.85 (s, 3H), 1.80 (d, *J* = 4.4 Hz, 6H). ESI-MS (ACN, *m*/*z*): 613.2 (calc. 613.2; 35%; {[Rh­(abpt)­(Cp^bph^)] – H}^+^), 649.1 (calc. 649.1; 100%; [Rh­(abpt)­Cl­(Cp^bph^)]^+^). IR (ATR, ν, cm^–1^): 3644w, 3563w, 3302s, 3193w, 3166w, 3099w, 3074w, 3061w, 3030w, 2971w, 2915w, 2381w, 2348w, 2310w, 2252w, 2228w, 2203w, 2167w, 2120w, 1993w, 1982w, 1926w, 1745w, 1640w, 1606w, 1590m, 1568w, 1553w, 1521w, 1487m, 1452s, 1428m, 1396w, 1380w, 1338w, 1289m, 1255m, 1193w, 1157m, 1108m, 1075w, 1034s, 1021w, 1005w, 909m, 875w, 835vs, 793s, 759w, 751m, 730m, 692s, 646w, 632w, 610w, 590w, 556s, 518w, 483w, 456w.

### UV–Vis Absorption and Emission Spectra

The experiments were performed using UV–vis spectrophotometers Varian Cary 4000 (Agilent) or Cintra 3030 (GBC Scientific Instruments) and Cary Eclipse Fluorescence Spectrometer (Agilent). Emission quantum yields (ϕ_em_) were determined using a Cary Eclipse Fluorescence Spectrometer (Agilent).

### Reversed-Phase High-Performance Liquid Chromatography (RP-HPLC)

RP-HPLC was performed on the UHPLC-MS device (Dionex/Thermo Fisher Scientific) equipped with a Phenomenex Luna (C18 stationary phase; 3 μm particle size, 100 Å pore size, 125 × 4 mm). The mixture of 0.1% trifluoroacetic acid (tfa) in H_2_O (A) and MeCN (B) was used as the mobile phase at the gradients of 20% B (*t* = 0 min), 80% B (*t* = 15 min) and 80% B (*t* = 20 min) with 0.6 mL/min flow rate. Alternatively, 0.1% formic acid or 0.1% ammonium formate was used instead of TFA.

### Stability Studies

The complexes **1**–**3** (250 μM final concentration) were dissolved in 270 μL DMF-*d*_7_, and 330 μL PBS in D_2_O was added. These solutions were analyzed by ^1^H NMR at *t* = 0–24 h (spectra were referenced to the residual DMF-*d*_7_ signal at 8.03 ppm). Similarly, **1**–**3** (50 μM final concentration) were dissolved in 1% DMF in H_2_O with or without PBS, and the mixtures were analyzed by UV–vis spectroscopy, ESI+ mass spectrometry, and HPLC. These ^1^H NMR, UV–vis, and MS experiments were performed at different time points (*t* = 0–24 h) with the mixtures kept in the dark and after 10 min of irradiation by blue light. Further, UV–vis photochemical experiments (10 min irradiation) were also performed in various mixtures of DMF and water containing 1–45% of DMF.

For the control experiments, the dehalogenated complexes **1**–**3** (50 μM final concentration) were synthetically prepared by their reaction with a stoichiometric amount of silver­(I) triflate in 1% DMF in H_2_O. After stirring for 2 h in the dark, the formed precipitate of AgCl was removed, and the obtained solutions were analyzed.

### Interactions with Small Biomolecules

Mixtures of **1**–**3** (50 μM final concentration) were prepared involving 5 mol equiv of NADH or 5 mol equiv of NAD^+^ with 25 mol equiv of sodium formate in 1% DMF in H_2_O with PBS. These mixtures were either irradiated (blue light, 10 min) or nonirradiated, and analyzed by UV–vis spectroscopy and ESI+ mass spectrometry at *t* = 0–24 h.

Analogical experiments were performed by ^1^H NMR in 1% DMF-*d*_7_ in D_2_O with PBS. These ^1^H NMR experiments were performed with the same irradiation as for the UV–vis experiments (single irradiation for 10 min) or with the mixtures kept in the dark.

^1^H NMR experiments were also performed on the mixtures of complexes (50 μM final concentration) with 5 (gradual irradiation up to 20 min) or 250 (gradual irradiation up to 60 min) molar equivalents of NADH in 1% DMF-*d*_7_ in D_2_O with PBS.

All the obtained ^1^H NMR spectra were referenced to the residual D_2_O signal (4.75 ppm). Turnover number (TON) and turnover frequency (TOF) values were calculated from integrated characteristic NADH and NAD^+^ resonance.

### DFT Calculations

Theoretical calculations were performed in ORCA 5.0 quantum chemistry software.^51^ For optimization of molecular geometry of **1**, BP86 (for preliminary run) and B3lYP functionals (for final optimization and TDDFT) were used.^52–57^ Basis sets were chosen from Ahlrich’s def2 set, QZVP base for Rh, SVp for H, and TZVP base for the rest of the atoms.^58,59^ Auxiliary base set def2/J and RIJCOSX approximation, along with the largest integration grid (DefGrid3) and tightSCF convergence criteria, were used in all calculations.^60–62^ Avogadro software was used for visualization.^63^

### Cell Lines and Culture Conditions

Human breast adenocarcinoma cells MDA-MB-231, human ovarian carcinoma A2780, and human skin melanoma cells A375 were purchased from the European collection of authenticated cell cultures ECACC (Salisbury, UK). Cells A549, derived from human lung adenocarcinoma tissue, were kindly provided by Professor B. Keppler, University of Vienna (Austria). Human colon cancer cell line HCT116 and MRC5pd30 cells derived from normal lung tissue were obtained from ATCC, Manassas, VA, USA. All the cell lines except A2780 were cultured in DMEM growth medium (high glucose, 4.5 g L^–1^, Biosera); A2780 cells were grown in RPMI-1640 medium (Biosera, Boussens, France). Both media were supplemented with gentamycin (50 mg mL^–1^) and 10% heat-inactivated FBS (Biosera); media for the MRC5 cells were further fortified by 1% nonessential amino acids (Sigma-Aldrich, Prague, Czech Republic). Cells were cultivated in a humidified atmosphere at 37 °C, 5% CO_2_, and subcultured twice a week to achieve the desired plating density. Stock solutions of Rh complexes intended for biological experiments were prepared in dimethylformamide (DMF) and subsequently diluted into the experimental medium. The final concentration of DMF in all biological assays did not exceed 0.5% (v/v). For the negative controls, a matched DMF concentration was used.

### Antiproliferative Activity

The antiproliferative activity of the investigated complexes was determined using the commonly employed MTT assay. The cells were seeded on 96-well plates at a density of 4 × 10^3^ cells/well for HCT116 and A375 or 5 × 10^3^ cells/well for MDA-MB-231 and A2780 in 100 μL of the respective culture medium (DMEM or RPMI-1640). After overnight incubation, the cells were treated with a series of concentrations of the tested complexes diluted in culture medium. After 72 h, MTT was added to a final concentration of 0.125 mg mL^–1^ and incubated for 3–4 h. Then, the medium was removed, and the resulting formazan product was dissolved in DMSO. Absorbance was read at 570 nm (620 nm reference) using an Absorbance Reader (SPARK TECAN, SCHOELLER). IC_50_ values were calculated from curves constructed by plotting the relative absorbance (relative to the untreated control) versus drug concentration (μM).

### Intracellular Localization by Confocal Microscopy

HCT 116 cells were seeded on 35 mm glass-bottom confocal culture dishes (Mattek Co., MA, USA) at a density of 1.5 × 10^5^ cells/dish. After overnight incubation, the cells were treated with **1** (27 μM) in a phenol red-free medium and incubated for 5 h. The cells were then washed with PBS, and a drug-free culture medium was added. Samples were analyzed on a confocal laser-scanning microscope Leica TCS SP5 (Leica Microsystems GmbH, Wetzlar, Germany), λ_ex_ = 405 nm, and the emission was detected in the 450–650 nm range.

For the colocalization experiments, the treatment of cells with **1** was followed by staining of the cells using the mitochondria-specific dye MitoTracker Red (Thermo Fisher Scientific) or the endoplasmic reticulum-specific probe ER Tracker Red (Thermo Fisher Scientific) according to the manufacturer’s protocol. Samples were analyzed using a confocal laser-scanning microscope with the following parameters: Complex **1-** ex. 405 nm; detection window 430–600 nm; MitoTracker Red - ex 581 nm, detection window 650–720 nm; ER Tracker Red - ex 587 nm; detection window 610–720 nm. Samples were excited sequentially in the frameswitching mode to eliminate a possible fluorescence overlap. Image processing and calculations of Pearson’s colocalization coefficients were performed using ImageJ software, and the Costes regression method for estimation of the threshold was used. Values of PCC are expressed as the mean ± SDs above the calculated threshold.

### Nanoparticle Tracking Analysis (NTA)

The solution/suspension of complex **1** was characterized using Nanoparticle Tracking Analysis (NTA). A stock solution of complex **1** was prepared at a concentration of 5 mM in DMF. This stock solution was then diluted to a final concentration of 27 μM in high-purity MQ water. The particle suspension was analyzed under two conditions: immediately after dilution and again after 24 h. NTA was performed using a NanoSight NS300 instrument (Malvern Panalytical, UK). The NTA software employed a mathematical model of finite track length adjustments to compensate for limited trajectories in Brownian motion, thereby mitigating broadening effects.^64^ Each analysis consisted of three replicate samples. For each sample, a 60-s video was captured (1498 frames), with a minimum of 100 particles per frame to ensure a statistically robust measurement.

### Phototoxicity

The effect of irradiation with visible (blue) light on the biological action of **1**–**3** in cancer cells was determined as described earlier^15,65^ against human melanoma A375 cells. The cells were seeded in 96-well tissue culture plates (5 × 10^3^ cells/well in 100 μL of complete DMEM medium) and cultured overnight in a humidified incubator. Subsequently, the medium was replaced by the tested complexes diluted in EBSS, and cells were incubated for 60 min in the dark, followed by 1 h of blue light (or sham) irradiation. The cells were irradiated using an LZC-4 photoreactor (Luzchem Research, Gloucester, Canada) equipped with 16 LZC-420 lamps with a maximum centered at 420 nm (50 W m^2–^). Control cells were incubated and irradiated with complex-free EBSS containing the same concentration of DMF (always less than 0.5%) as in the treated cells. It was verified that the concentration of DMF in vehicle controls and irradiation in complex-free media did not affect cell viability. After irradiation, the EBSS medium containing the complexes was removed, and the cells were cultured in a drug-free, complete DMEM medium for 70 h. The number of living cells in the samples was determined using a standard MTT assay (*vide supra*), and the IC_50_ values were obtained from the dose–response curves. The PTI was calculated as the ratio of IC_50_ (dark) to IC_50_ (irradiated).

To estimate the long-term effect on normal, noncancerous cells, human lung fibroblasts (MRC5pd30) cells were seeded in 96-well plates at a density of 4 × 10^3^ cells/well and incubated for 48 h in complete DMEM medium containing **1**–**3**. After the incubation period, an MTT assay was performed and the results were evaluated.

### Morphology Studies

A375 cells (1 × 10^5^ cells per 35 mm confocal dish were treated with Rh complexes (1 μM) or the respective vehicle (DMF) control in EBSS. Samples were kept in the dark for 1 h and then irradiated with blue light (420 nm). The Rh-containing EBSS was then removed, replaced with Rh-free DMEM medium, and the cells were further incubated at 37 °C. Samples were observed at the indicated intervals and imaged under an inverted microscope (Olympus CKX41).

### Intracellular ROS Determination

A375 cells were seeded on 12-well plates and treated with the tested complexes in EBSS at the indicated concentrations in the dark for 1 h. After 30 min, CellROX Green reagent (Life Technologies) was added to the cells. Then, the solution was replaced by fresh, compound-free EBSS, and cells were irradiated as described above. Afterward, the cells were analyzed by a real-time live cell imaging system, JuLI Stage (NanoEntek). Bright-field and fluorescence (excitation = 466 nm, emission = 525–550 nm) channels were recorded.

Solution of 12.5 μM DHR 123 was prepared in PBS (1% DMF). To this solution, Rh-complex was added at a final concentration of 10 μM. Then, samples were irradiated by blue light (420 nm, 5 mW cm^–2^) for the indicated time intervals. Fluorescence spectra were recorded using a Varian Cary Eclipse spectrofluorophotometer with the following parameters: a right-angled configuration, a 0.5 cm quartz cuvette, an excitation wavelength of 488 nm, an emission wavelength of 510–630 nm, and excitation and emission slit widths of 5 nm.

The nature of the ROS generated after treatment with **1** was evaluated by determining its phototoxicity in the presence of selective ROS scavengers, such as mannitol, sodium pyruvate, sodium azide, and tiron. A375 cells were pretreated with the particular ROS scavenger for 1 h at 37 °C, then treated with **1** and irradiated. After a 72-h recovery period, cell viability was assessed using the MTT test.

### Propidium/Annexin Staining

Cells were seeded in 40 mm culture dishes at a density of 2.5 × 10^5^ cells per dish and treated with the specified concentrations of Rh complexes, followed by irradiation as previously described. After treatment, the Rh-containing EBSS was removed, and the cells were incubated for an additional 22 h in drug-free medium. Subsequently, cells were harvested via trypsinization, washed with cold PBS (4 °C), and stained with propidium iodide (10 μg mL^–1^) and Annexin V–FITC (1.5 μL per 150 μL of cell suspension; Thermo Fisher Scientific) for 15 min at room temperature. The stained cells were immediately analyzed using a BD FACSVerse flow cytometer, and the data were processed with FCS Express 6 software (DeNovo Software, Glendale, CA). Representative dot plots from two independent experiments are presented.

### Porimin Immunostaining

A375 cells were either left untreated or treated with the Rh-complex at a concentration equivalent to 3 × IC_50_ (refer to Table 2), followed by irradiation as already described. After a 4-h recovery period in compound-free medium, cells were fixed with 4% paraformaldehyde on microscope slides and permeabilized using 0.1% Triton X-100. Subsequently, cells were incubated overnight with the primary antibody Porimin G-2 (sc-377295, Santa Cruz). After washing, a fluorescently labeled secondary antibody (ab150113, Abcam) was applied for 1 h. Excess dye was removed by washing, and cell nuclei were counterstained with DAPI. Samples were examined using a Leica TCS SP5 confocal microscope.

### 3D Culture Experiments

HCT116 cells (550 cells/well) were seeded on 96w ultralow attachment U-shape plates (Corning) in the spheroid forming medium: DMEM-F12 ham medium supplemented with 2% B27 (Thermo Fisher Scientific Inc., MA, USA), epidermal growth factor (EGF; Sigma-Aldrich, Germany, 20 ngmL^–1^), fibroblast growth factor (FGF2; Sigma-Aldrich, Germany, 10 ng mL^–1^), and bovine serum albumin (BSA) (Sigma-Aldrich, Germany, 0.15%). After 48 h of incubation, preformed spheroids were transferred as single spheres to 12-well chamber μ-Slides (Ibidi) and treated with the tested complexes at a concentration of 6 μM for 5 h, and following that, the spheroids were washed and irradiated with 405 nm laser light for 5 min at the final power of 1 mW. Spheroids were cultured for a further 24 h postirradiation and, after this period, were stained with Hoechst 33258 (20 μg mL^–1^), calcein AM (2 μM), and PI (8 μg mL^–1^) for 2 h. Samples were imaged on a confocal microscope, Leica CM SP5 (Leica, Germany), in 10 z-stack scans. Images were processed using ImageJ software.

For the detection of Rh-complex penetration into 3D spheroids derived from HCT-116 cells, the spheroids were formed as previously mentioned. The spheroids were treated with the tested complexes at a concentration of 2 μM for 5 h, and subsequently, they were washed and transferred to 12-well chamber μ-Slides (Ibidi). Rh complexes were excited with a 405 nm blue laser (1 mW), and the detection window was set from 450 to 650 nm. Samples were imaged on a confocal microscope, Leica CM SP5 (Leica, Germany). Images were processed using ImageJ software. Bright-field images shown in the Supporting Information (Figure S38) were obtained separately on an Olympus CKX41 phase contrast microscope.

## Supplementary Material





## Notes

Structures of complexes 1, 2, and 3; ligand L1; and anthracene-9-carbaldehyde (CSV)
Figures S1–S10, S12, S18–S22, S24–S31, ^1^H NMR, ESI-MS spectra, and RP HPLC traces of the investigated compounds; Figures S11, S16, S17, and S23, UV–vis absorption spectra of the investigated compounds; Figure S13, determination of the localization of **1** within cellular organelles; Figure S14, Nanoparticle tracking analysis (NTA) of **1**; Figures S15 and S32, microscopic study of the morphology of A375 cells treated with **1** or **2**; Figure S33, detection of ROS generation in A375 cells; Figure S34, fluorescence emission of DHR 123 + **1** irradiated with blue light; Figure S35, viability of A375 cells treated with **2** and irradiated in the presence of ROS scavengers; Figures S36–S38, effect of the investigated complexes on 3D tumor spheroids; Table S1, DFT calculations; and molecular formula strings­(PDF)

## Abbreviations Used

AC: anthracene-9-carbaldehyde
ACN: acetonitrile
Cp^bph^: 1-biphenyl-2,3,4,5-tetra-methylcyclopentadienyl
DEE: diethyl ether
DFT: density functional theory
DEE: diethyl ether
DMEM: Dulbecco’s modified Eagle’s medium
DMF: dimethylformamide
DMSO: dimethyl sulfoxide
EBSS: earle’s balanced salt solution
EGFR: epidermal growth factor receptor
ESI-MS: electrospray ionization mass spectrometry
FAK: focal adhesion kinase
HCp*: pentamethylcyclopentadiene
IC_50_: concentration of the compound reducing cell growth by 50%
ILTC: intraligand charge transfer
LLCT: ligand-to-ligand charge transfer
Mim: 1-methylimidazoleabpt = 3,5-di­(pyridin-2-yl)-4H-1,2,4-triazol-4-amine
MLCT: metal-to-ligand charge transfer
MTT: 3-[4,5-dimethylthiazol-2-yl]-2,5 diphenyl tetrazolium bromide
NADH: nicotinamide adenine dinucleotide
PBS: phosphate-buffered saline
phen: 1,10-phenanthroline
PI: propidium iodide
PTI: phototoxic index
Pta: phosphine 1,3,5-triaza-7-phosphatricyclo[3.3.1.1]­decane
Py: pyridine
ROS: reactive oxygen species
SD: standard deviation
tfa: trifluoroacetic acid
