# Supplementary material for: Phototoxicity of Half-Sandwich Rhodium(III) Complexes with Anthracene and Biphenyl Substituents toward Mammalian Cancer Cells and Multicellular Tumor Spheroids
Source: J Med Chem. 2025 Sep 18;68(19):20360–76. doi: 10.1021/acs.jmedchem.5c01406 (PMC12516717; doi:10.1021/acs.jmedchem.5c01406)
Supplement: Supplementary file 2 [file jm5c01406_si_002.pdf]

Supporting Information

for

Phototoxicity of Half-sandwich Rhodium(III) Complexes with Anthracene and Biphenyl Substituents toward Mammalian Cancer Cells and Multicellular Tumor Spheroids

Petra Andršová[a], Jitka Prachařová[a], Vojtěch Novohradský[c], Slavomíra Šterbinská,[b] Pavel Štarha[b], Jana Kašpárková[a], Viktor Brabec[a*]

[a]*Department of Biophysics, Faculty of Science, Palacky University, Slechtitelu 241/27, CZ-77900 Olomouc, Czech Republic*
[b]*Department of Inorganic Chemistry, Faculty of Science, Palacky University, 17. listopadu 1192/12, CZ-77146 Olomouc, Czech Republic*
[c]*Czech Academy of Sciences, Institute of Biophysics, Kralovopolska 135, CZ-61200 Brno, Czech Republic*

*Corresponding author's e-mail address:
e-mail: vbrabec44@gmail.com (V. Brabec)

Table of Contents

Figures S1-S4 1H NMR spectra of compound L1 and complexes 1-3 (dissolved in DMSO-d6) ……………………………………..…………………………………… S3

Figure S5 ESI-MS spectra of complexes **1**–**3** …..…………………………………………… S5

Figures S6, S7 RP-HPLC traces of complexes **1**–**3** ……………………………………….. S6

Figures S8-S10 ^{1}H NMR studies of complexes **1-3** in 45% DMF-d_7/55% PBS in D_2O (pH 7.4) in the dark, as observed at different time points ……………………………..…… S8

Figure S11 UV-Vis absorption spectra of complexes **1**–**3** ……………………………….. S11

Figure S12 ESI+ mass spectra given for complex **1** ……………………….…………. S12

Figure S13 Determination of the localization of **1** within cellular organelles …………… S12

Figure S14 Nanoparticle tracking analysis (NTA) of **1** …..……………………………… S13

Figure S15 Morphology of A375 cells treated with **1** for 3 h in the dark and subsequently irradiated, as observed on confocal microscopy ………………………………………... S13

Figure S16 UV-Vis absorption spectra of complexes **1**–**3**, compounds L1, abpt, and anthracene-9-carbaldehyde ……….……………………………………………………..… S14

Table S1 Examples of DFT-calculated transitions involved in the UV-Vis peak centered at approximately 425 nm ……………………………………………………………………………… S15

Figure S17 UV-Vis absorption spectra of complexes **1**–**3** dissolved in various mixtures of water (with PBS) and DMF ……………………………………………………………………………...S16

Figures S18-S20 ESI-MS spectra of complexes **1-3** without and with irradiation (λ = 425 nm) ………………………………………………………………………………… S17

Figure S21 RP-HPLC traces of non-irradiated and irradiated (blue light) complex **1** …… S20

Figure S22 RP-HPLC traces of complexes **1** and **3**, and anthracene-9-carbaldehyde (AC) irradiated for 10 min (blue light) in 1% DMF in 99% PBS in water ……………………… S21

Figure S23 UV-Vis absorption spectra of the mixtures of complexes **1–3** with NADH or NAD^+ and sodium formate ….……………………………………………...………... S22

Figures S24-S28 ^1H NMR studies of **1-3** mixed with NADH after irradiation with blue light ……………………………………………………………………...….S23

Figures S29-S31 ^1H NMR studies of **1-3** mixed with NAD^+ and sodium formate after irradiation with blue light ……………………………………………………….… S28

Figure S32 A. Effect of irradiated **2** on cell morphology. B. Immunofluorescence staining of porimin in A375 cells treated with **2**. C. Quantitative evaluation of fluorescence intensity in cells immunolabeled for porimin. D. PI/annexin V staining of A375 cells treated with **2** and irradiated.. S31

Figure S33 Detection of ROS generation in A375 cells, untreated (control) or treated with **2**, **3** or AC …………………………………………………….……………… S32

Figure S34 Fluorescence emission of DHR 123 + **1** irradiated with blue light …........….. S32

Figure S35 Viability of A375 cells treated with **2** and irradiated in the presence of ROS scavengers ……………………………………………………………………….… S33

Figure S36 Distribution of complexes **1** and **2** in HCT116 spheroids visualized by confocal microscopy ……………………………………………………………………….... S33

Figure S37 Analysis of the ratio of mean fluorescence intensity of PI and calcein ……..... S34

Figure S38 Phase contrast microphotographs of HCT116 spheroids, untreated or treated with complexes **1** or **2** and blue light irradiation ……………………………………………….. S34

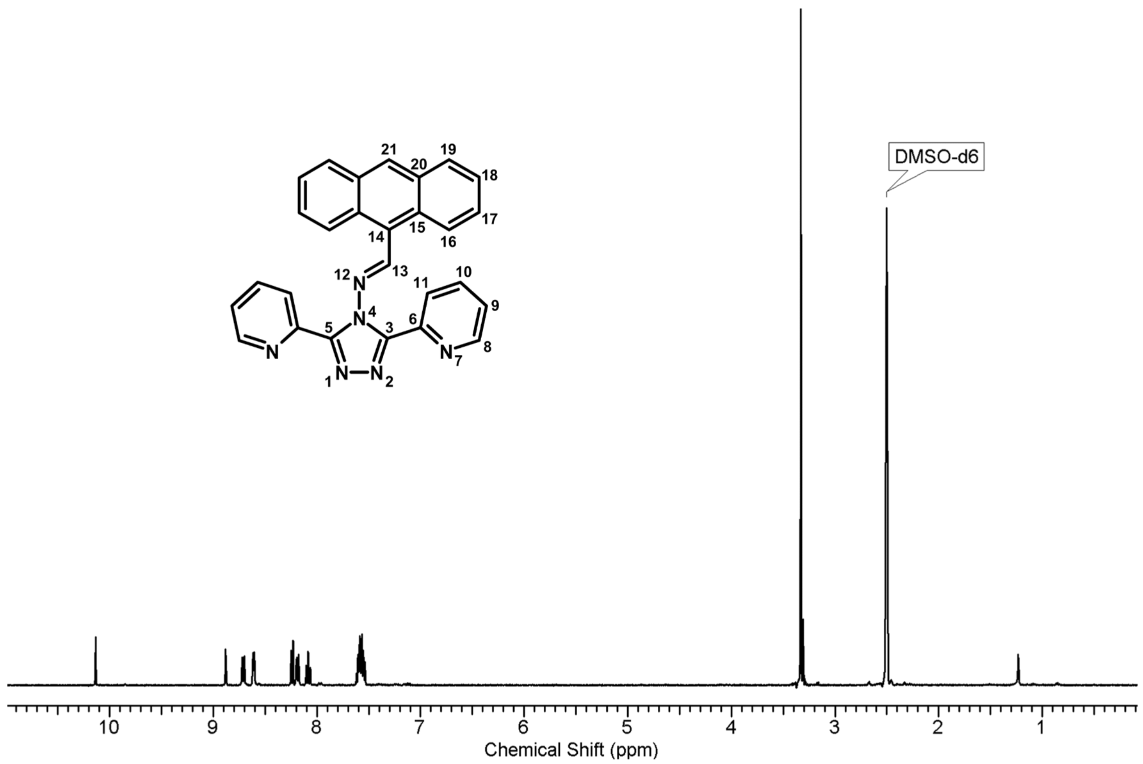

Figure S1. ^{1}H NMR spectrum of compound L1 (dissolved in DMSO-d_6).

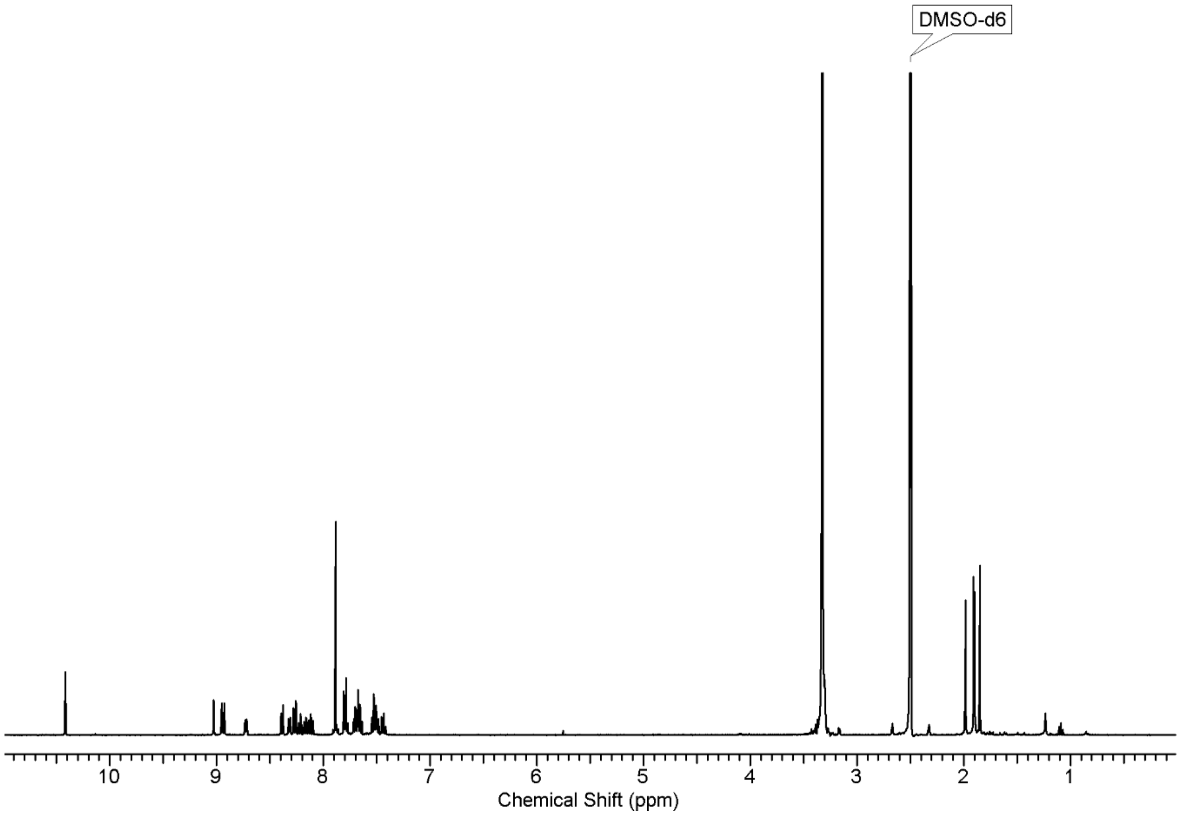

Figure S2. ^{1}H NMR spectra of complex **1** (dissolved in DMSO-d_6).

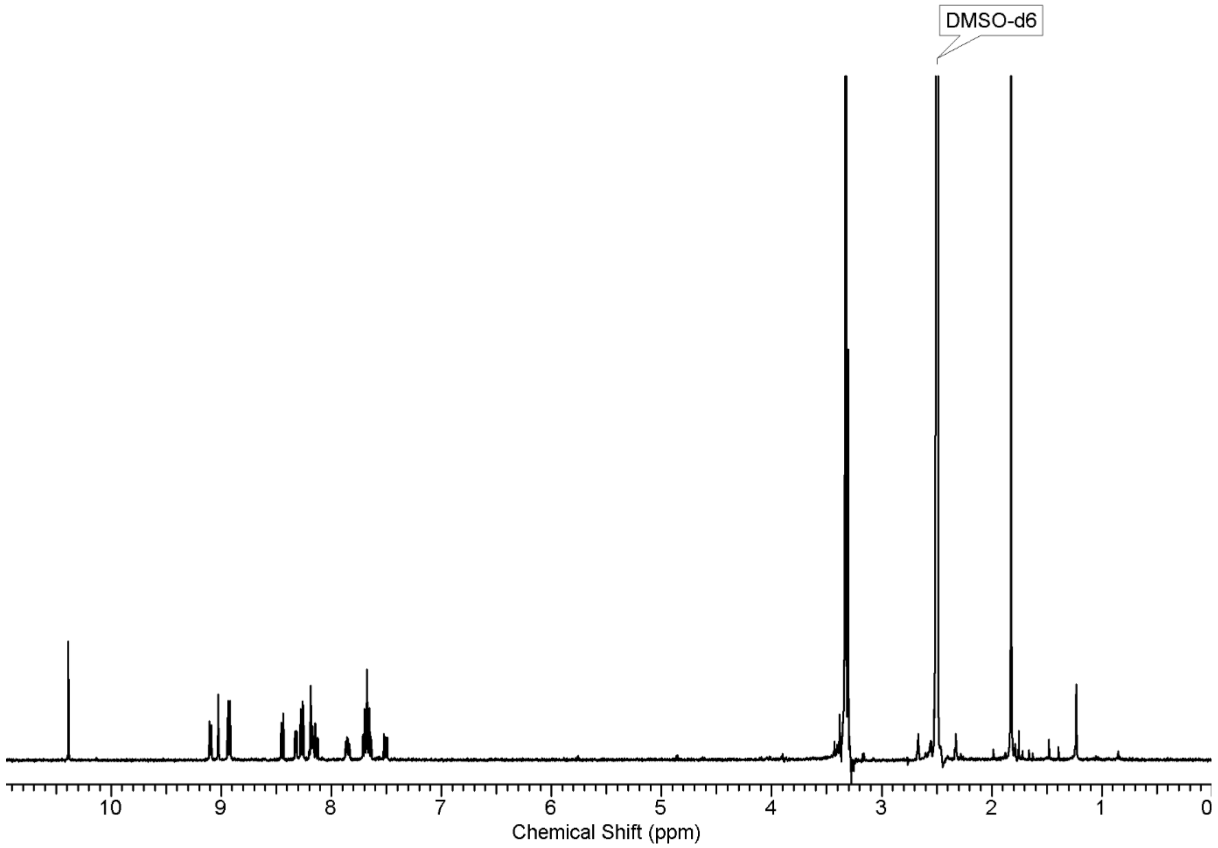

Figure S3. ^{1}H NMR spectra of complex **2** (dissolved in DMSO-d_6).

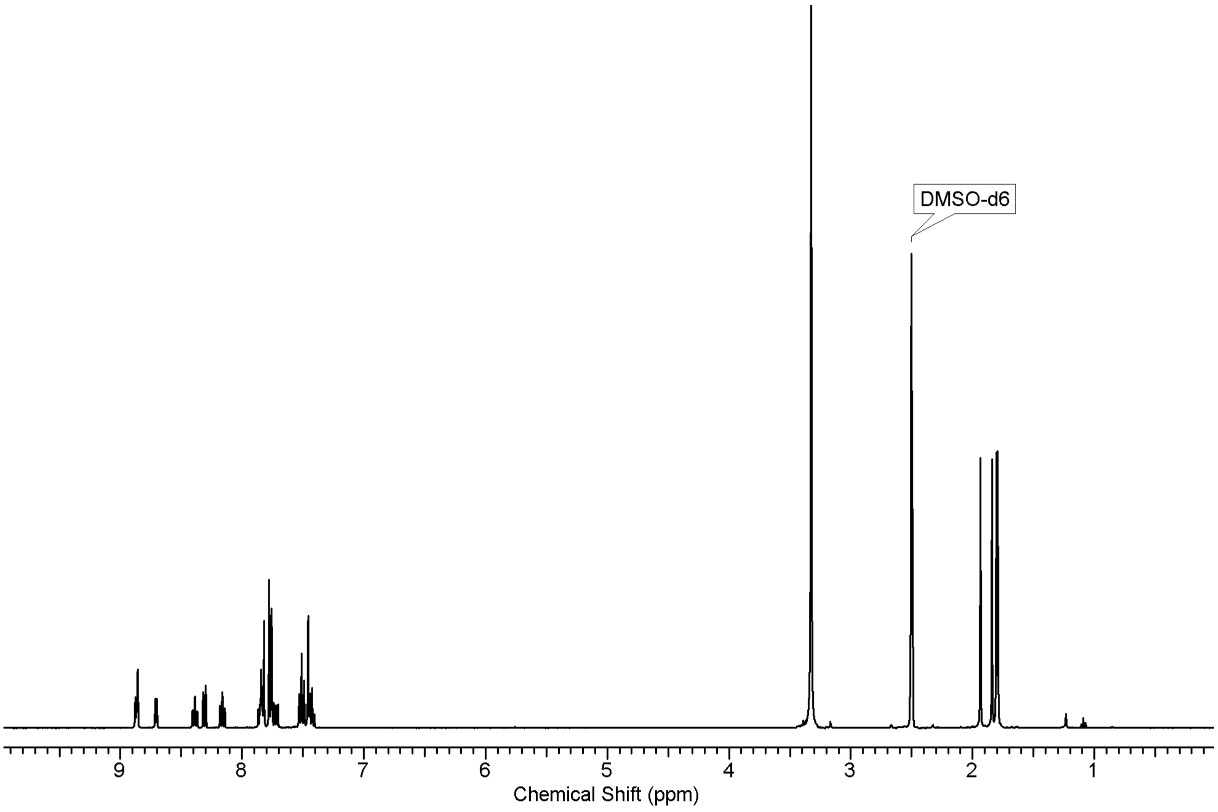

Figure S4. ^{1}H NMR spectra of complex **3** (dissolved in DMSO-d_6).

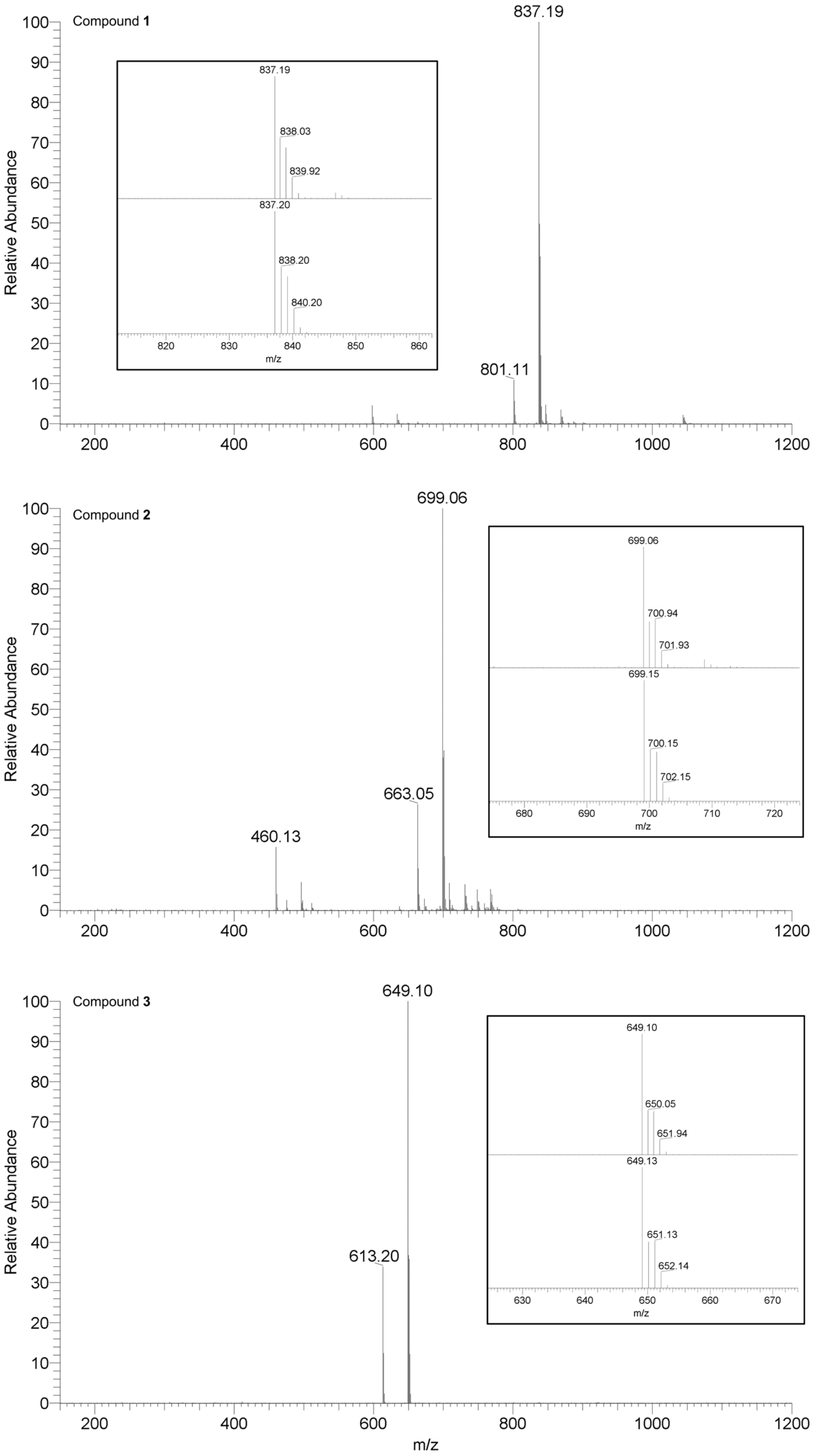

Figure S5. ESI-MS spectra of complexes **1**–**3** (dissolved in ACN), given with details of both the experimental (*inset top*) and simulated (*inset bottom*) isotopic pattern of the $[RhCl(Cp^x)(L)]^+$ species; ESI+ = positive electrospray ionization mode.

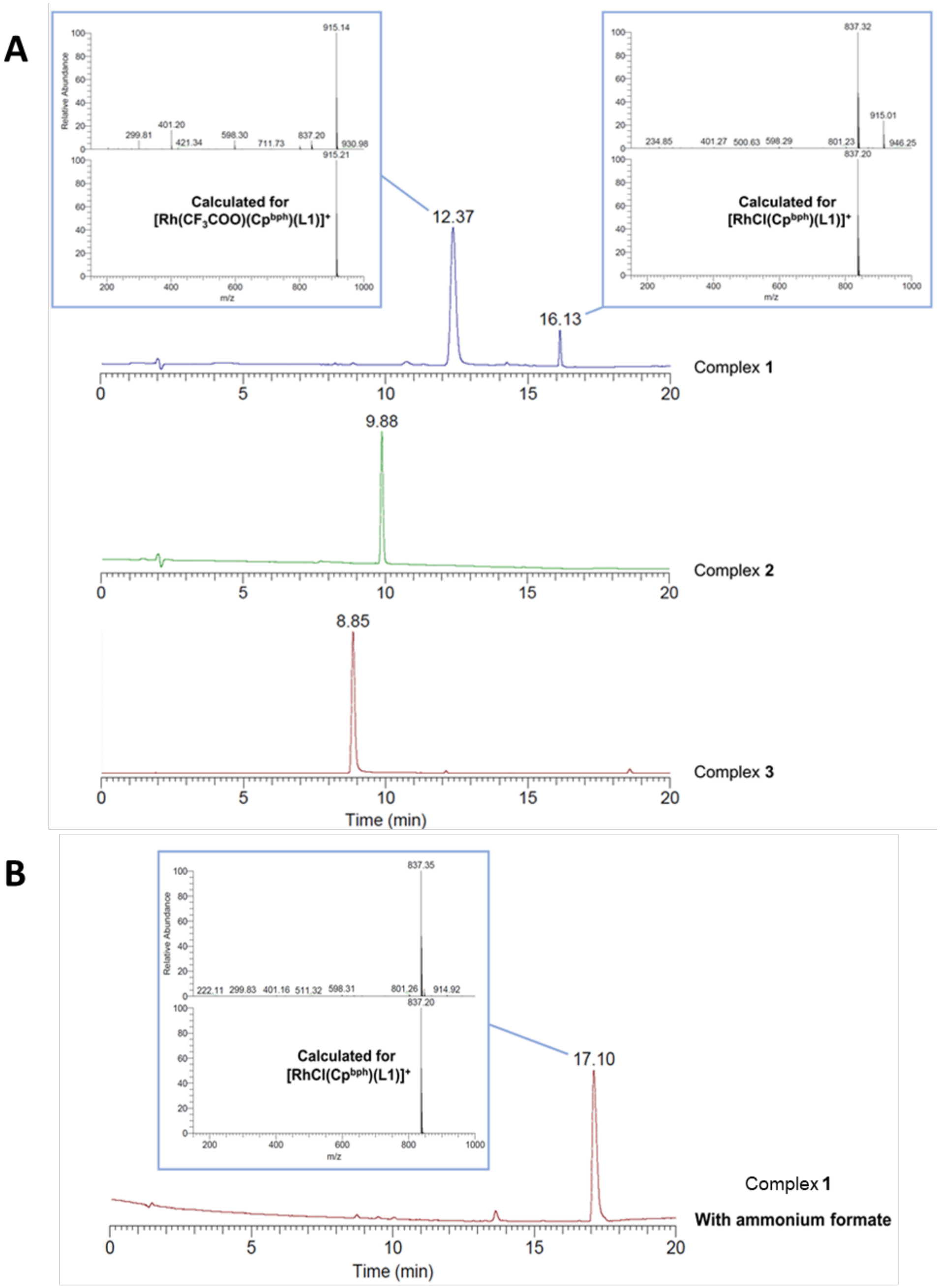

Figure S6. A. RP-HPLC traces of complexes **1**–**3** (0.1% trifluoroacetic acid in water:ACN). *Insets*: the results of coupled ESI+ mass spectrometry for **1**. B. RP-HPLC trace of complex **1** (0.1% ammonium formate in water:ACN). Inset: the results of coupled ESI+ mass spectrometry.

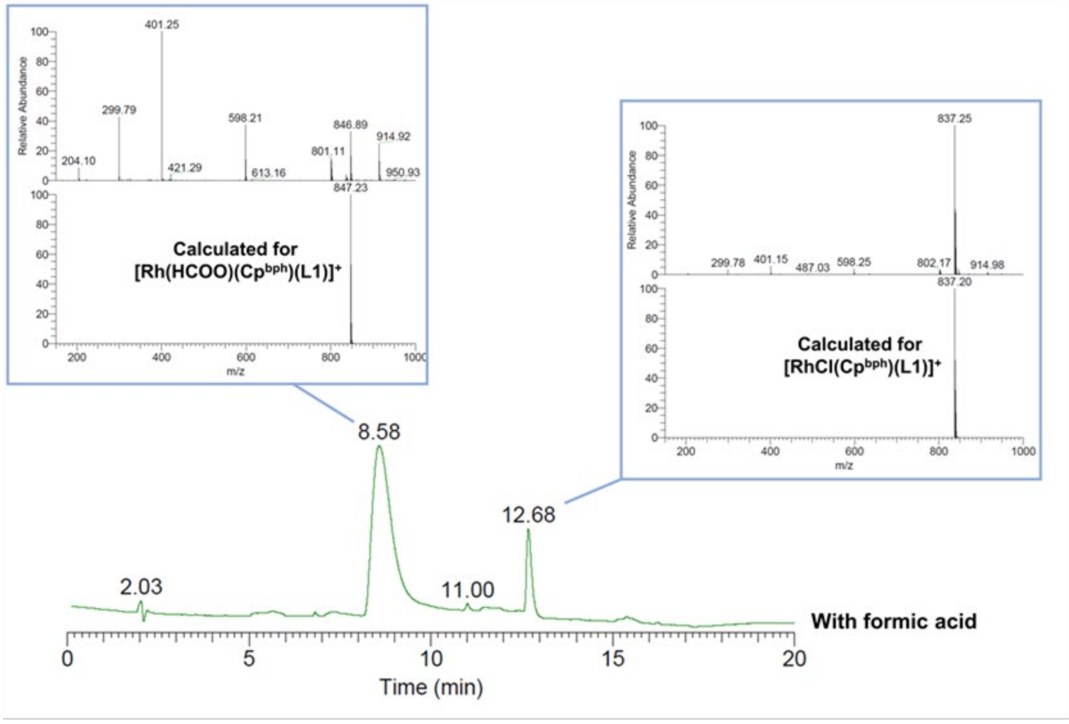

Figure S7. RP-HPLC trace of complex **1** (0.1% formic acid in water:ACN (top). Insets: the results of coupled ESI+ mass spectrometry.

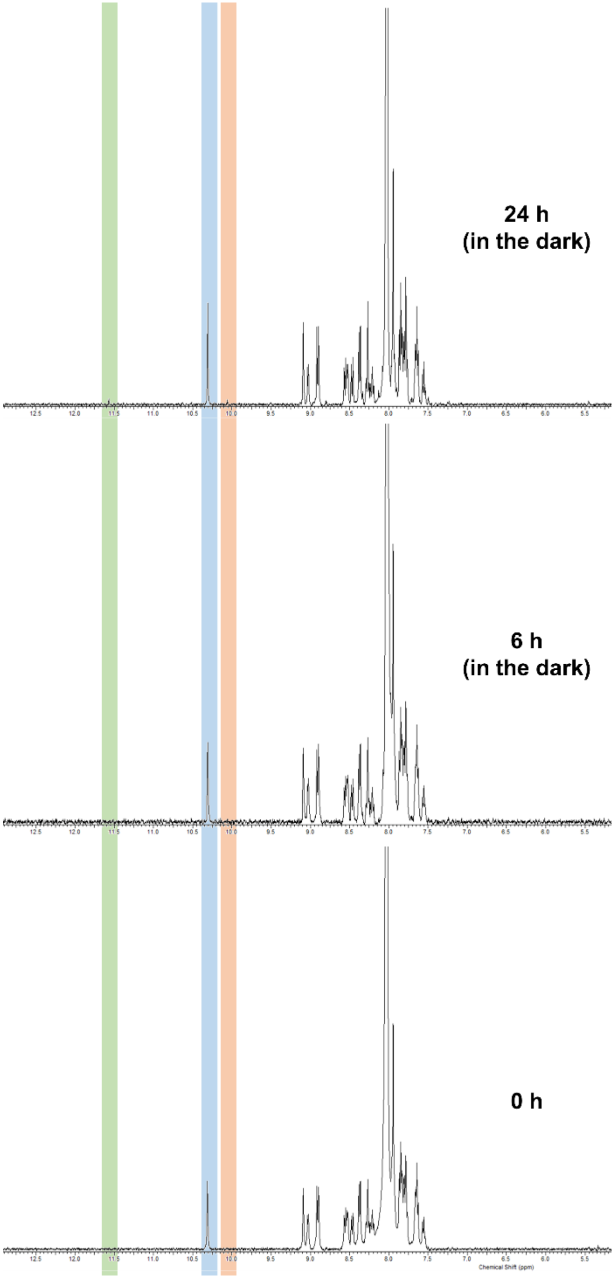

Figure S8. ^{1}H NMR studies of complex **1** in 45% DMF-d_7/55% PBS in D_2O (pH 7.4) in the dark, as observed at different time points (0 h, 6 h, and 24 h).

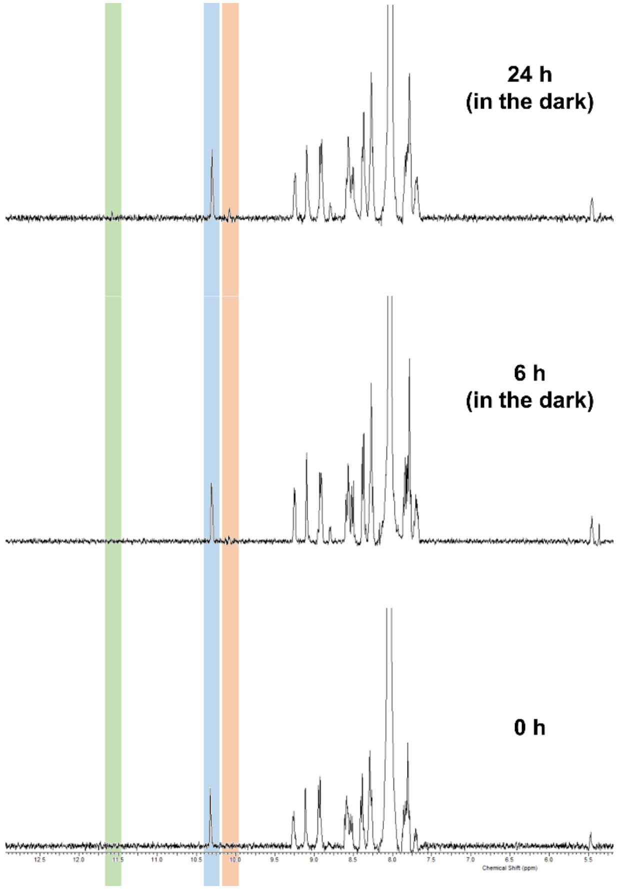

Figure S9. ^{1}H NMR studies of complex **2** in 45% DMF-d_7/55% PBS in D_2O (pH 7.4) in the dark, as observed at different time points (0 h, 6 h, and 24 h).

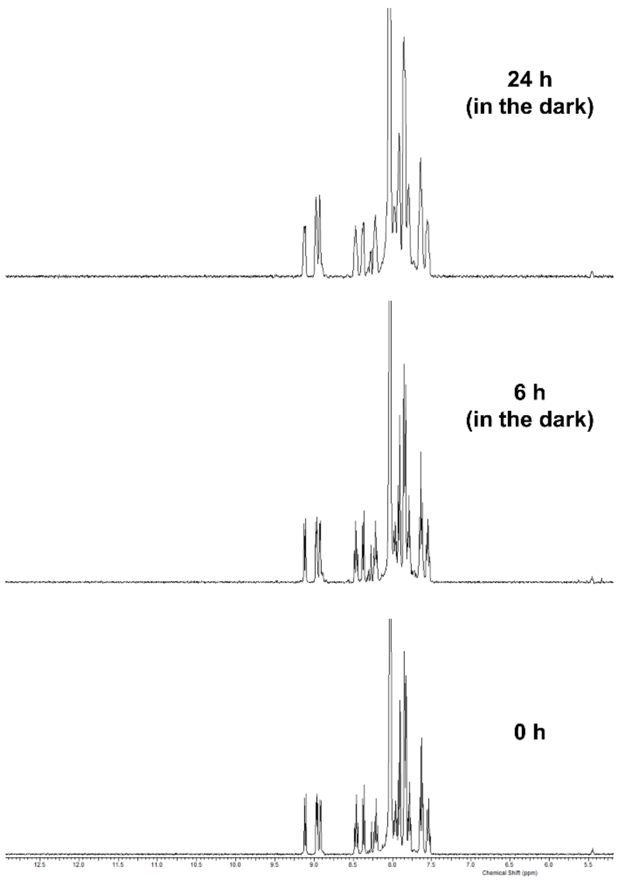

Figure S10. ^{1}H NMR studies of complex **3** in 45% DMF-d_7/55% PBS in D_2O (pH 7.4) in the dark, as observed at different time points (0 h, 6 h, and 24 h).

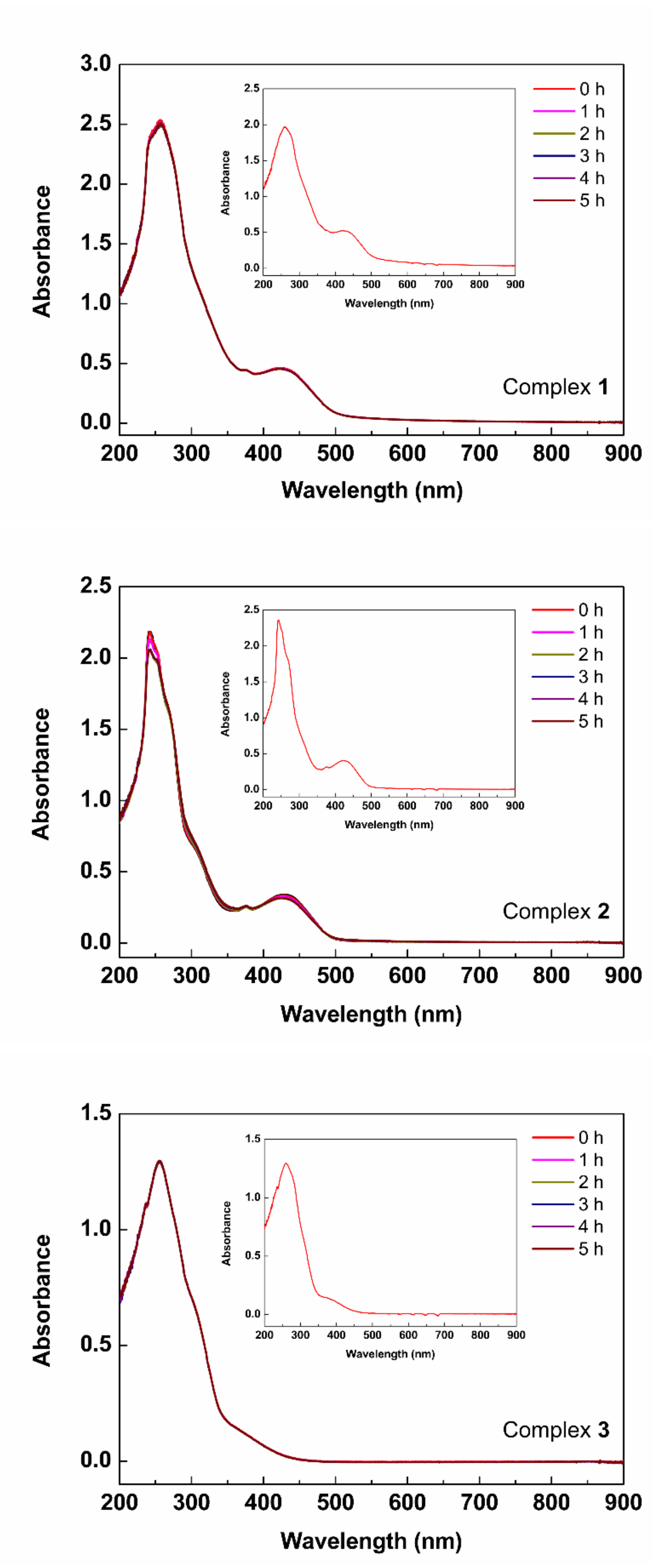

Figure S11. UV-Vis absorption spectra of complexes **1–3** dissolved in 1% DMF in water (without PBS) as observed at different time points (*inset*: control experiments performed with PBS).

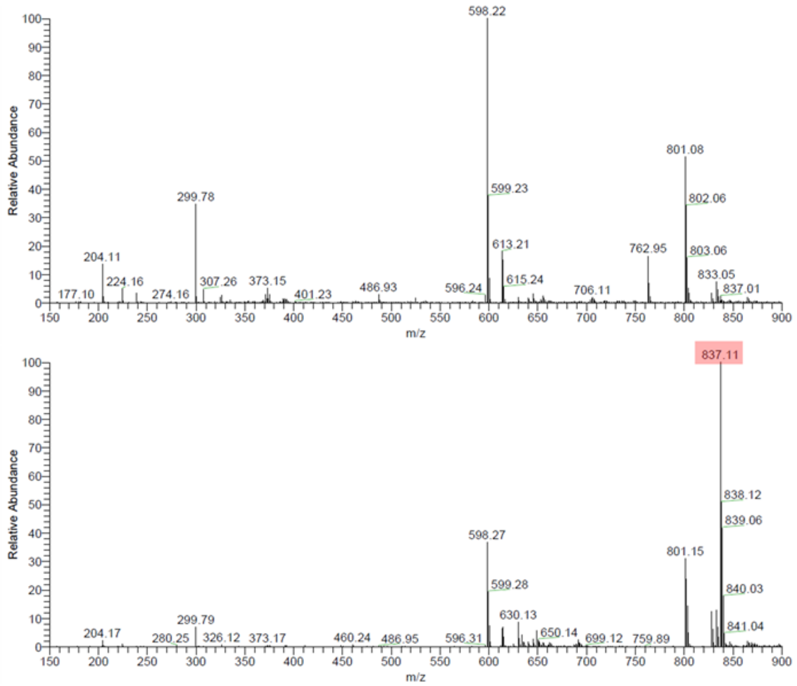

Figure S12. ESI+ mass spectra given for complex **1** (50 µM final concentration) incubated for 5 h in 1% DMF in H_2O (*bottom*) and for synthetically dehalogenated complex **1** (*top*). The red-labelled peak detected at 837.1 *m/z* belongs to the $[RhCl(Cp^{bph})(L1)]^+$ complex cation of **1**. ESI+ = positive electrospray ionization mode.

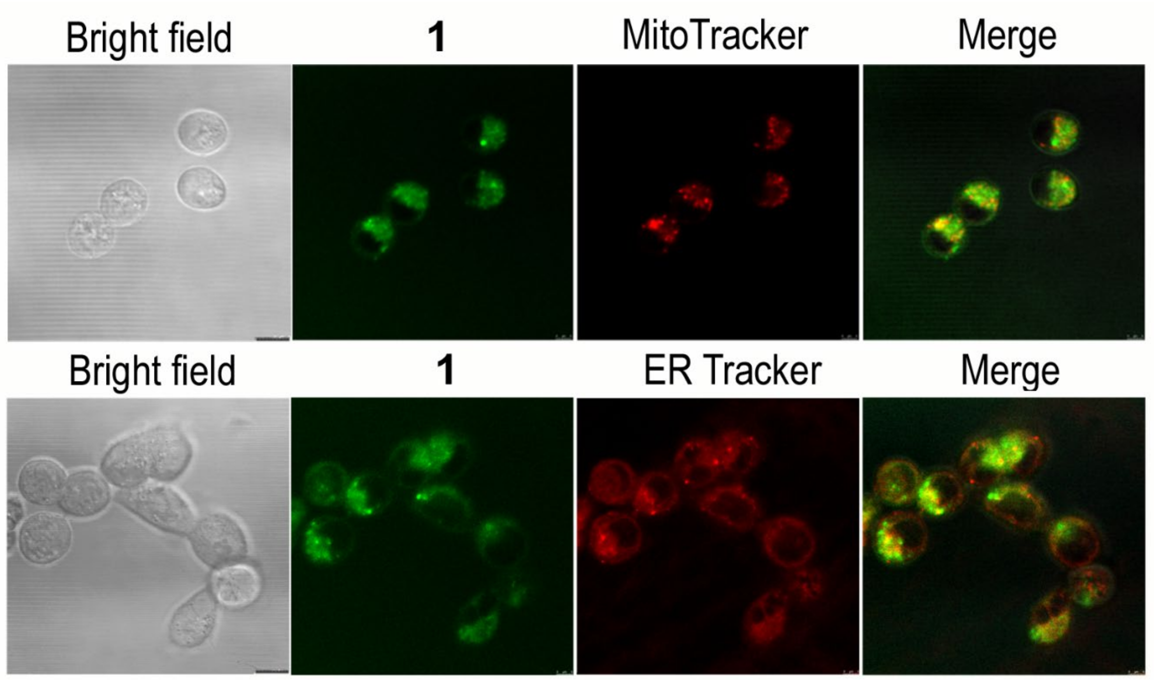

Figure S13. Determination of the localization of **1** within cellular organelles. Cellular colocalization assay of 1 with MitoTracker Red (top panels) and LysoTracker Red (bottom panels) in HCT116 cells. Scale bars indicate 10 µm. For experimental details, see the Experimental section.

A

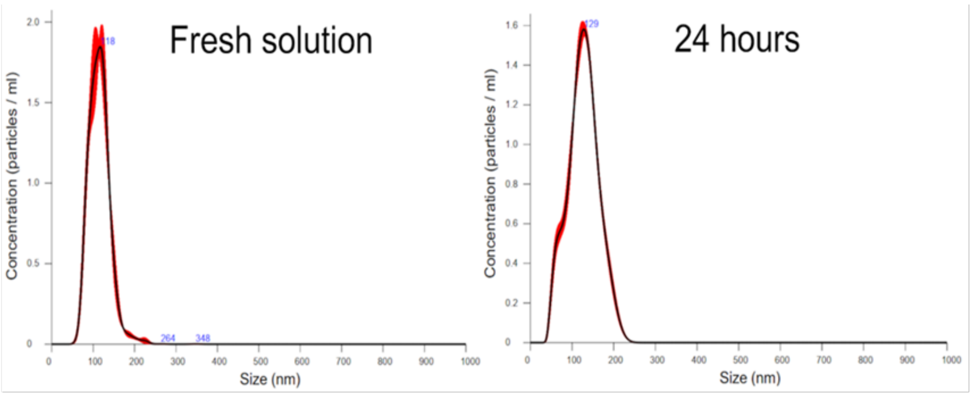

B

	Mean diameter ± SD (nm)	Concentration (10^9 particles/mL)
**1** (Fresh solution)	115.0 ± 26.0	1.15 ± 0.03
**1** (24 hours)	127.2 ± 37.1	1.40 ± 0.004
Solvent (0.5% DMF in H_2O)	101.3 ± 34.2	0.0013± 0.0002

Figure S14. Nanoparticle tracking analysis (NTA) of **1** in water/0.5% DMF. Stock solution of **1** was diluted with MQ water to a final concentration of 27 µM. A. The histograms display the particle concentration (particles/mL) versus the hydrodynamic diameter (nm), measured at both time 0 (immediately after dilution) and 24 h to assess for potential further aggregation. The raw data were subjected to the averaged finite track length adjustment (FTLA) analysis. B. Quantitative analysis of NTA results.

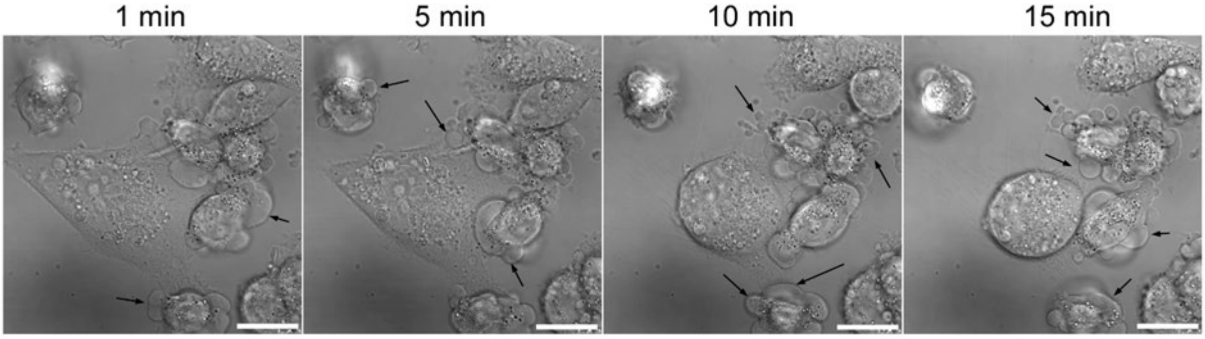

Figure S15. Morphology of A375 cells treated with complex **1** for 3 h in the dark and subsequently irradiated, as observed on confocal microscopy. Black arrows show membrane pertrusions and blebs. Scale bars indicate 20 µm.

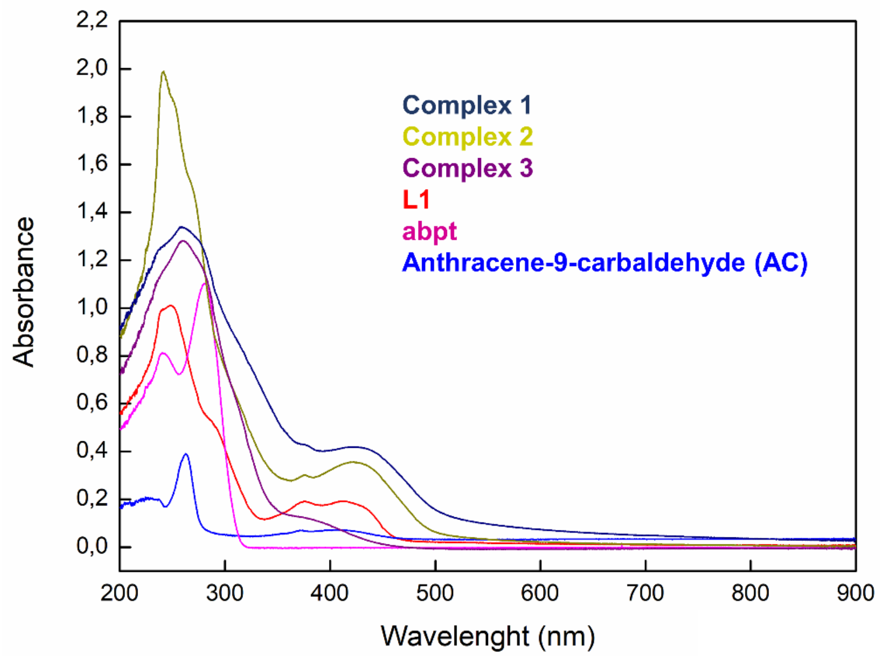

Figure S16. UV-Vis absorption spectra of complexes **1–3**, compound L1 (*N*-[1-(anthracene-9-yl)-methylidene]-3,5-di(pyridin-2-yl)-4*H*-1,2,4-triazol-4-amine), abpt (3,5-di(pyridin-2-yl)-4*H*-1,2,4-triazol-4-amine) and AC (anthracene-9-carbaldehyde); 50 µM, 1% DMF/99% H_2O.

Table S1. Examples of DFT-calculated transitions involved in the UV-Vis peak centered at approximately 425 nm, which have the most significant contributions to the given transitions (top), are shown with NTOs (*bottom*). DFT Calculations were performed in ORCA 5.0 software, using B3lYP functionals for final molecular geometry optimization and TDDFT (basis sets: QZVP base for Rh; SVp for H; TZVP other atoms). Avogadro software was used for visualization. Donor = occupied molecular orbital; acceptor = unoccupied molecular orbital. Transition types: ILCT = intra-ligand charge transfer; LLCT = ligand-to-ligand charge transfer; MLCT = metal-to-ligand charge transfer; LMCT = ligand-to-metal charge transfer.

**Donor → Acceptor**	**Energy (cm^{-1})**	**Transition type**
D1 → A1	20490	ILCT
D3 → A1	22252	LLCT
D1 → A3	24138	ILCT
D2 → A1	24735	MLCT
D1 → A2	25475	LMCT

Acceptor		
**1**	**2**	**3**
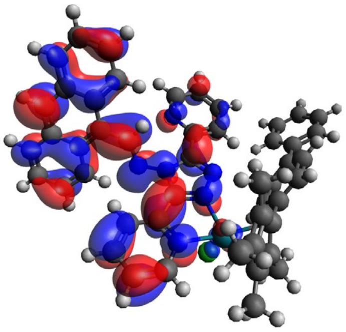	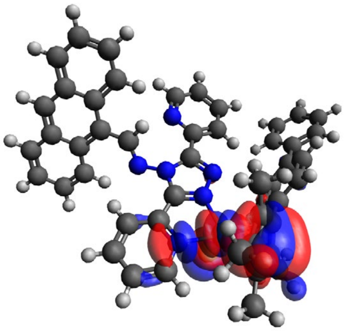	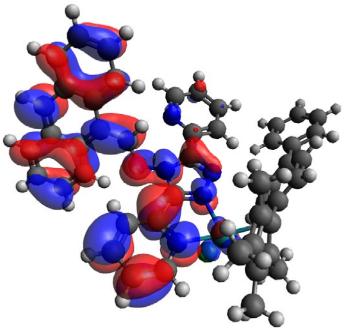

Donor		
**1**	**2**	**3**
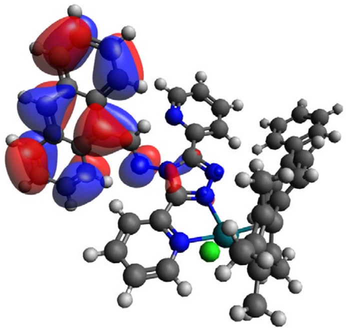	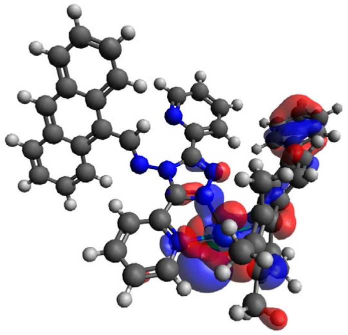	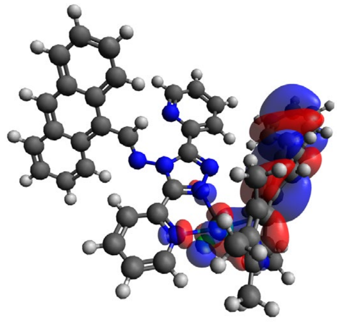

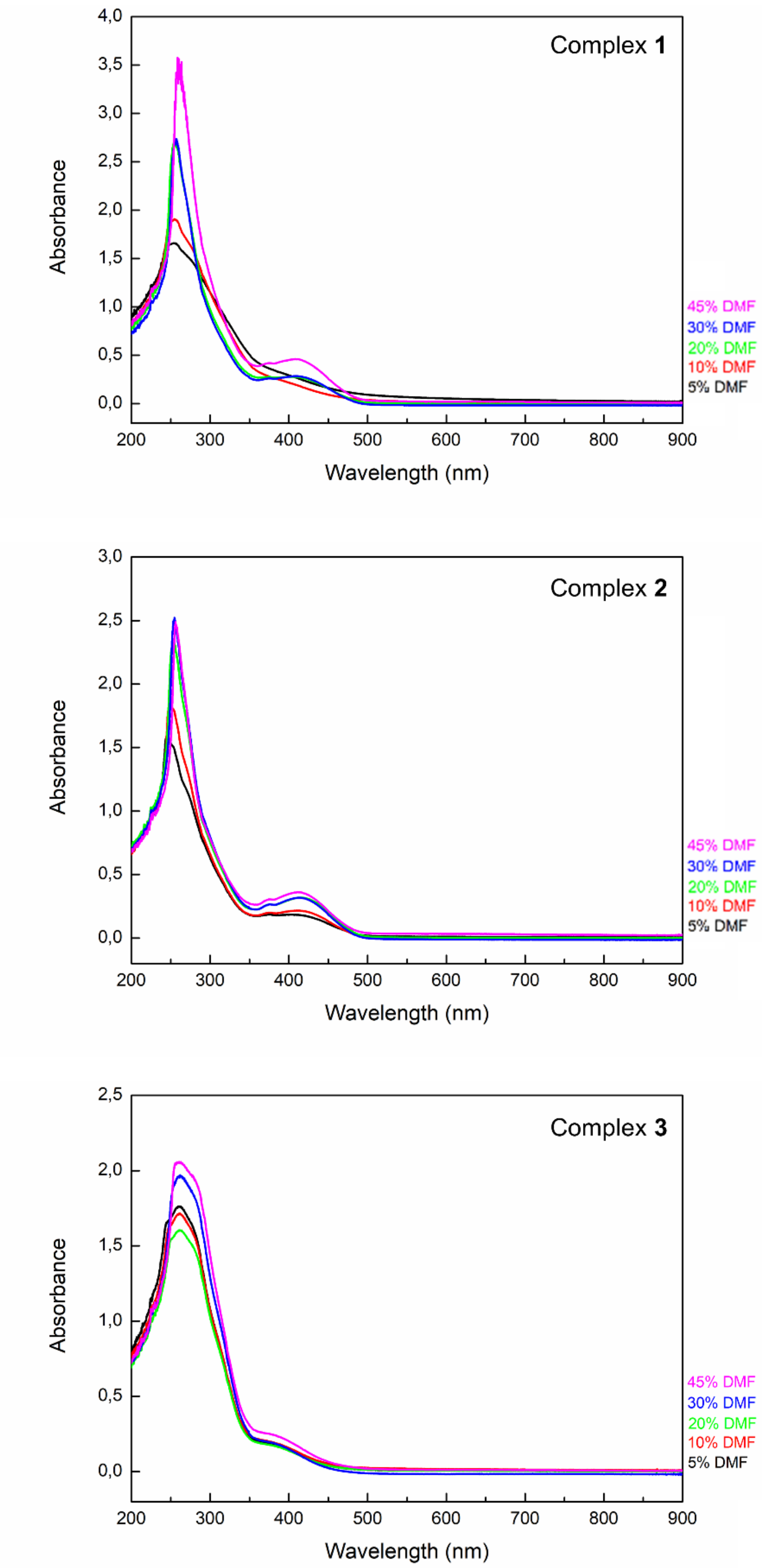

Figure S17. UV-Vis absorption spectra of complexes **1**–**3** dissolved in various mixtures of water (with PBS) and DMF, recorded after 10 min irradiation (λ = 425 nm).

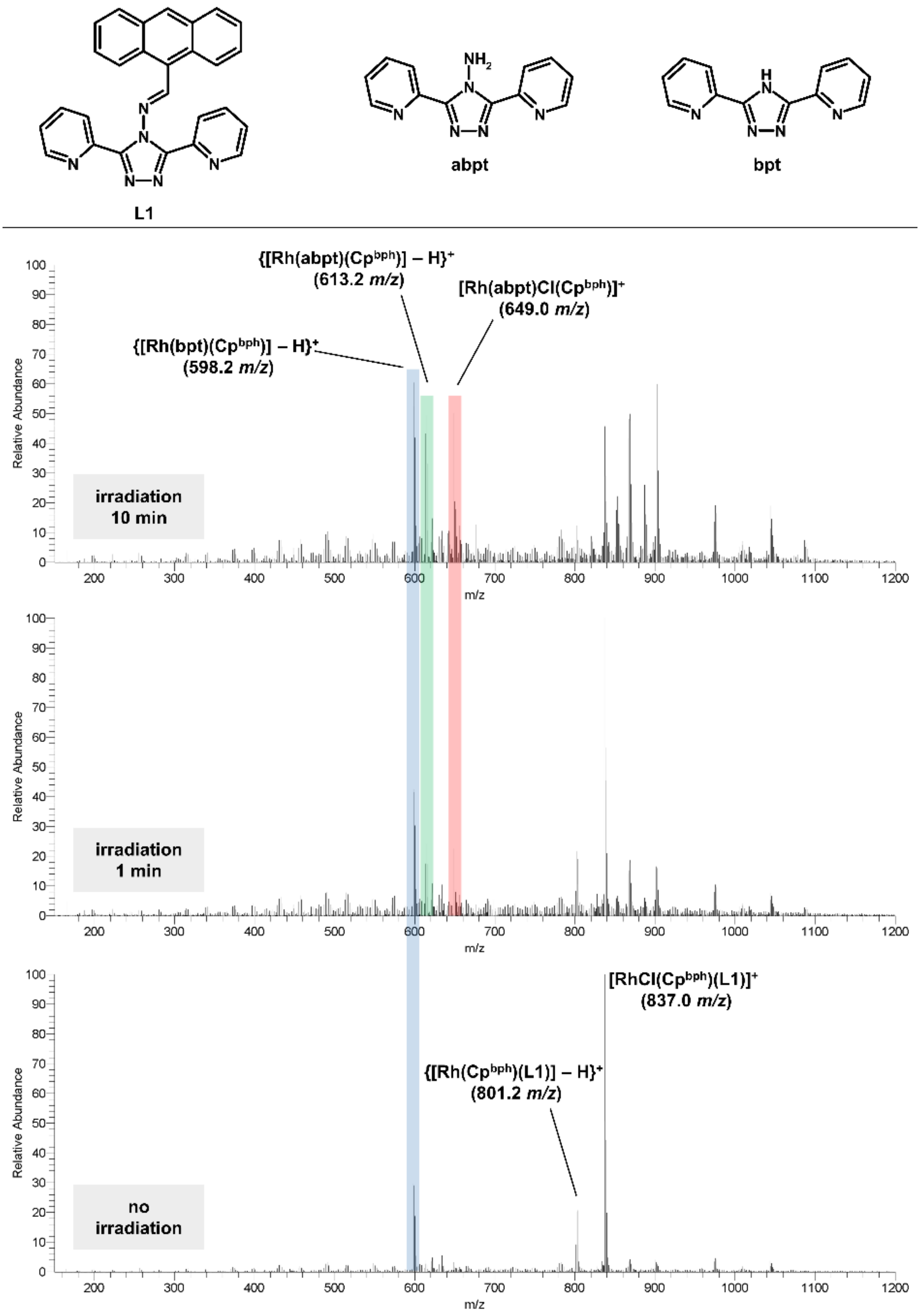

Figure S18. ESI+ mass spectra of complex **1** (50 µM final concentration in 1% DMF in H_2O with PBS) without irradiation (*bottom*) and with irradiation (λ = 425 nm) for 1 min (*middle*) or 10 min (*top*), along with the structural formulas of L1 and its fragments (abpt, bpt). ESI+ = positive electrospray ionization mode.

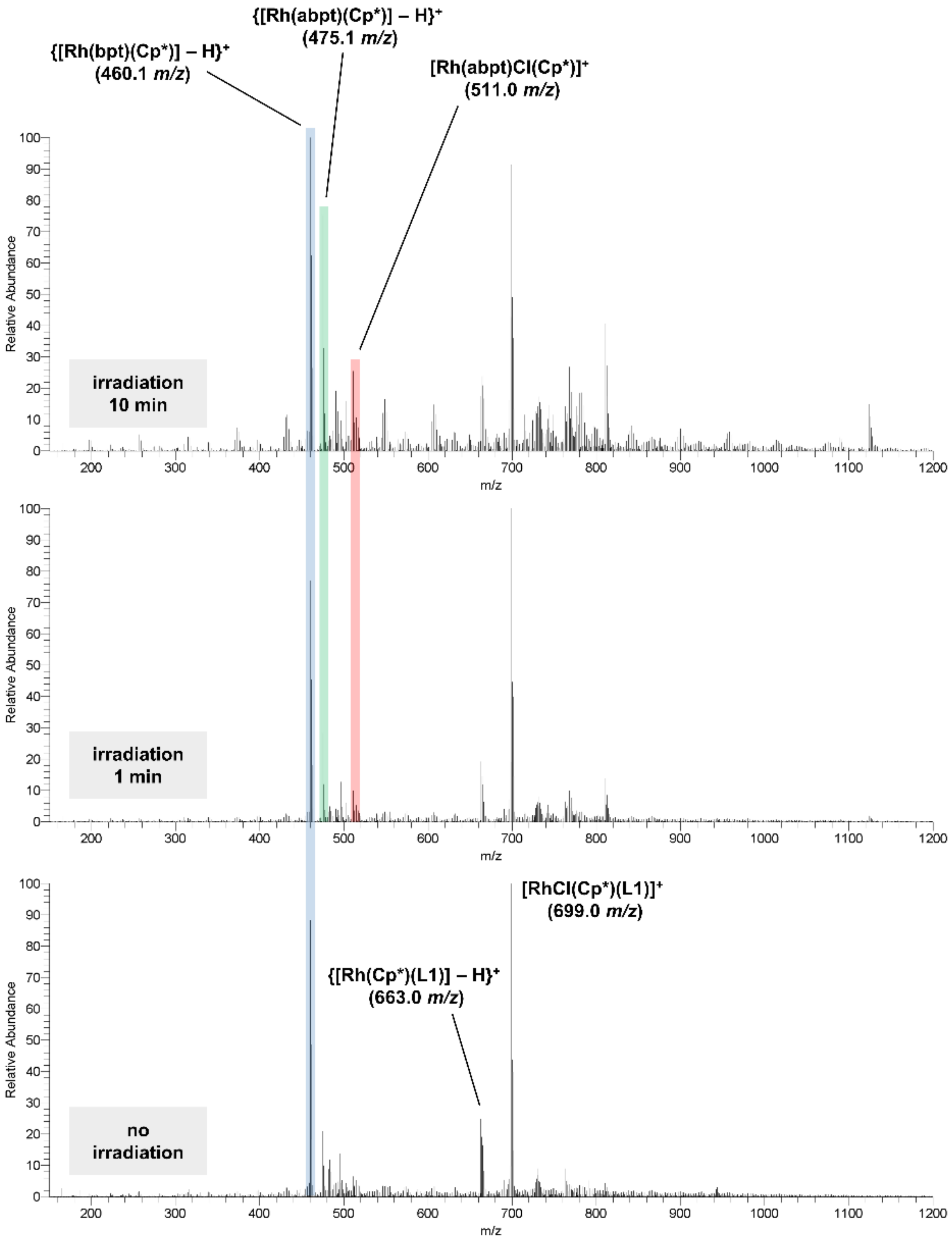

Figure S19. ESI+ mass spectra of complex **2** (50 µM final concentration in 1% DMF in H_2O with PBS) without irradiation (*bottom*) and with irradiation (λ = 425 nm) for 1 min (*middle*) or 10 min (*top*); ESI+ = positive electrospray ionization mode.

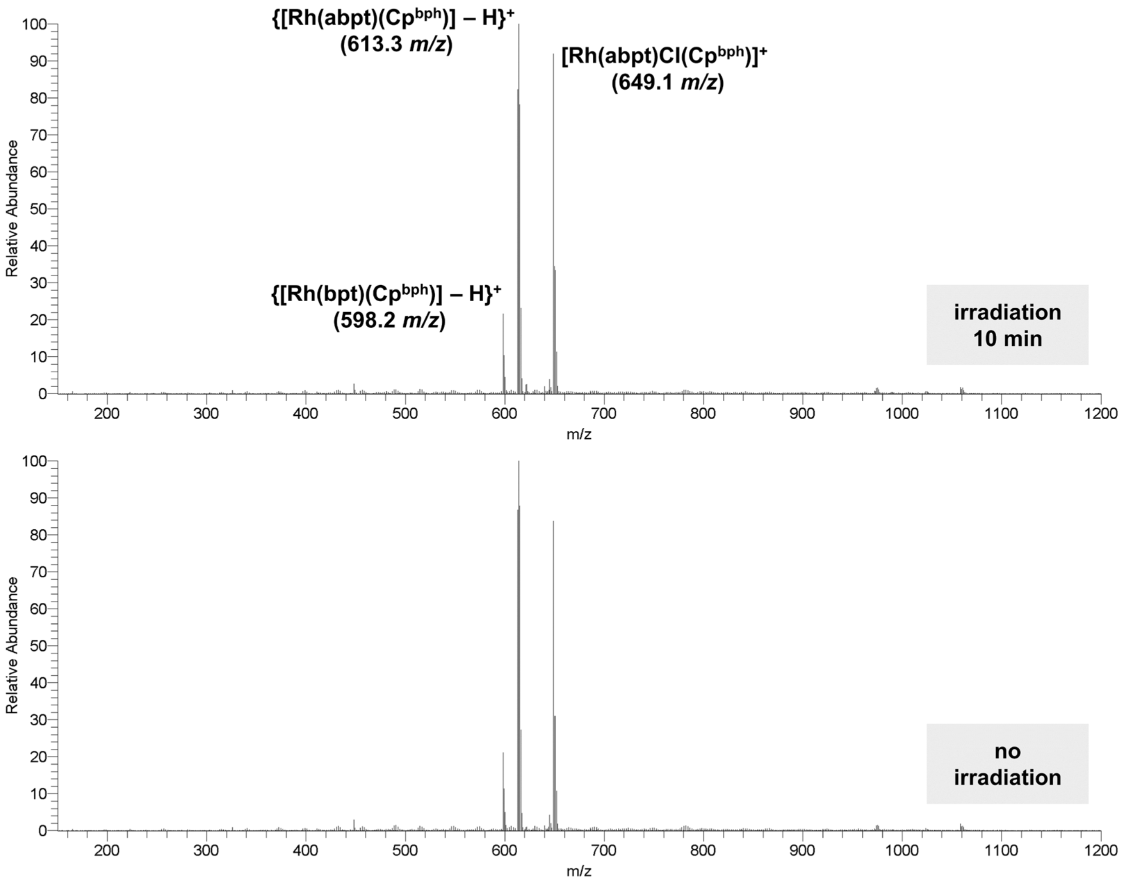

Figure S20. ESI+ mass spectra of complex **3** (50 µM final concentration in 1% DMF in H_2O with PBS) without irradiation (*bottom*) and with irradiation (λ = 425 nm) for 10 min (*top*); ESI+ = positive electrospray ionization mode.

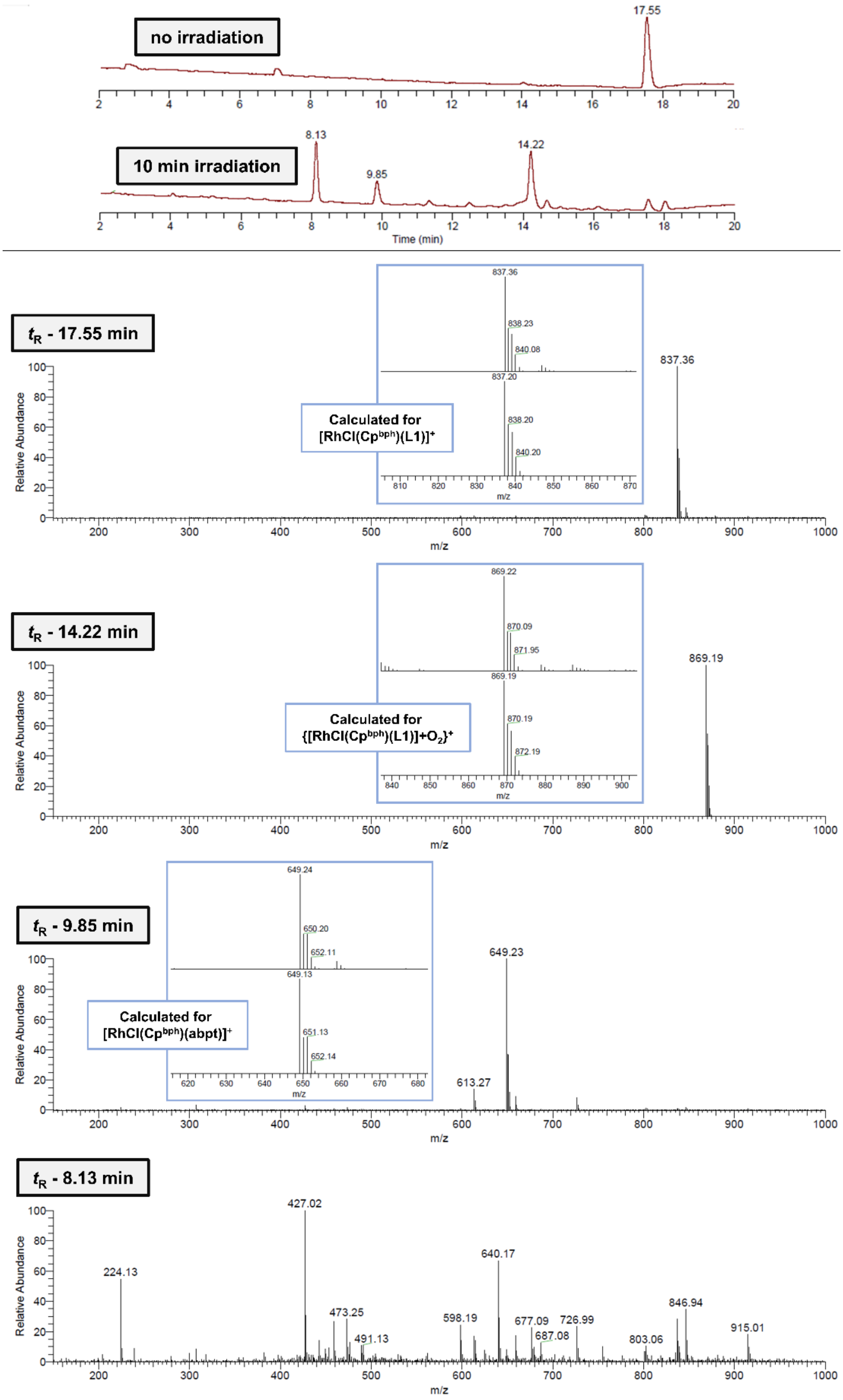

Figure S21. RP-HPLC traces of non-irradiated and irradiated (10 min, blue light) complex **1** in 1% DMF in 99% PBS in water (0.1% ammonium formate in water:ACN), given with mass spectra of the dominant HPLC peaks. Insets: a comparison of experimental (top) and calculated (bottom) isotopic patterns of dominant MS peaks.

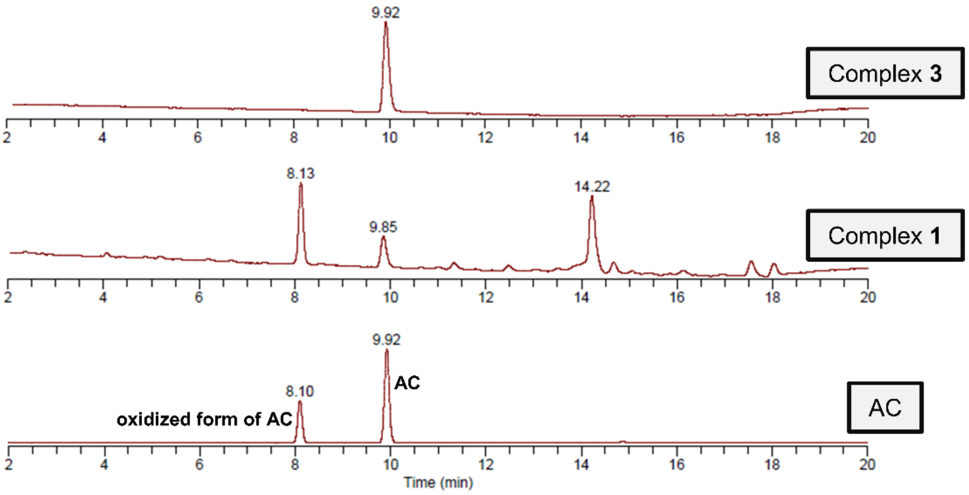

Figure S22. RP-HPLC traces of complexes **1** and **3**, and anthracene-9-carbaldehyde (AC) irradiated for 10 min (blue light) in 1% DMF in 99% PBS in water (mobile phase - 0.1% ammonium formate in water:ACN).

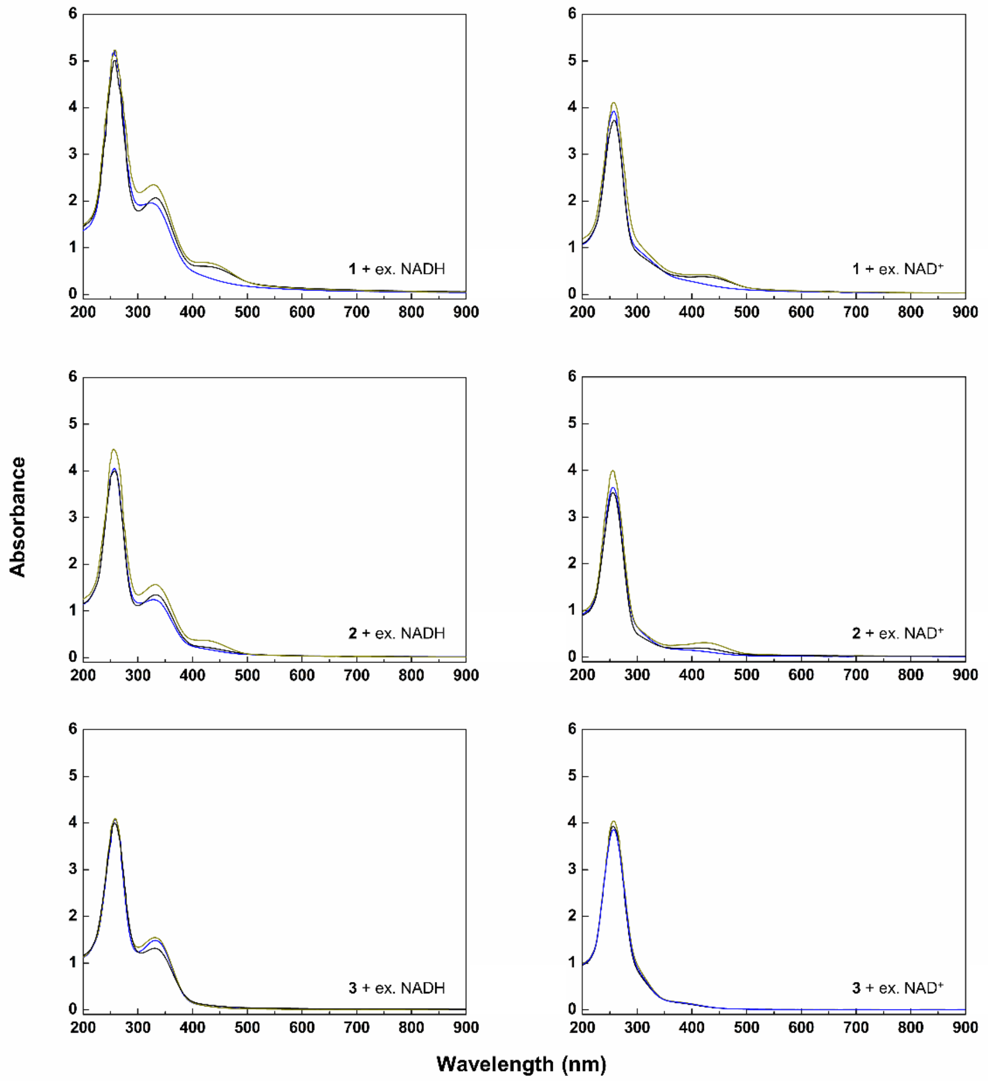

Figure S23. UV-Vis absorption spectra of the mixtures of complexes **1–3** (50 μM final concentration; 1% DMF/99% H_2O with PBS) with NADH (5 molar equivalents) or NAD^+ and sodium formate (5 and 25 molar equivalents, respectively) as observed on fresh samples and after 10 min irradiation (blue light). Color code: dark yellow - fresh mixtures; blue - mixtures after 10 min irradiation; black - mixtures after 24 h in the dark.

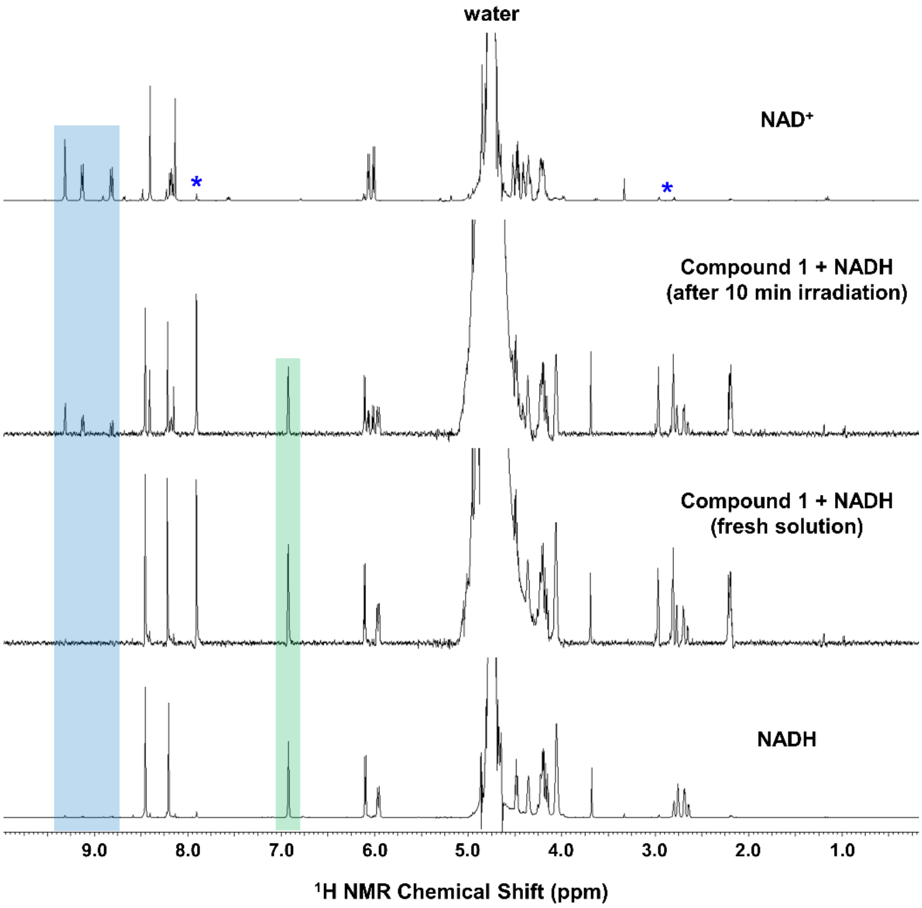

Figure S24. ^{1}H NMR studies of complex **1** mixed with NADH (1:5) in 1% DMF-d_7/99% PBS in D_2O (pH 7.4), as observed on a fresh sample and after 10 min irradiation (blue light). ^{1}H NMR spectra of both NAD(H) compounds are given for comparative purposes (measured in the same medium). Color code: blue - characteristic NAD^+ resonances; green - characteristic NADH resonance; blue asterisks – DMF.

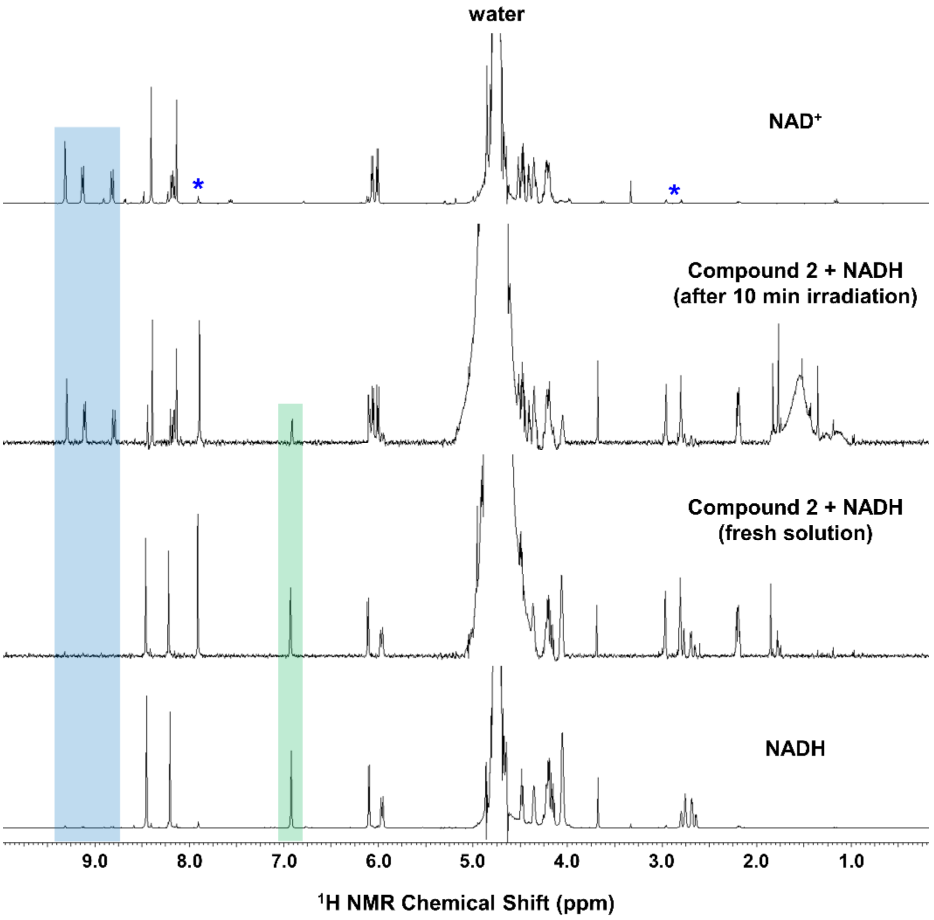

Figure S25. ^{1}H NMR studies of complex **2** mixed with NADH (1:5) in 1% DMF-d_7/99% PBS in D_2O (pH 7.4), as observed on a fresh sample and after 10 min irradiation (blue light). ^{1}H NMR spectra of both NAD(H) compounds are given for comparative purposes (measured in the same medium). Color code: blue - characteristic NAD^{+} resonances; green - characteristic NADH resonance; blue asterisks - DMF.

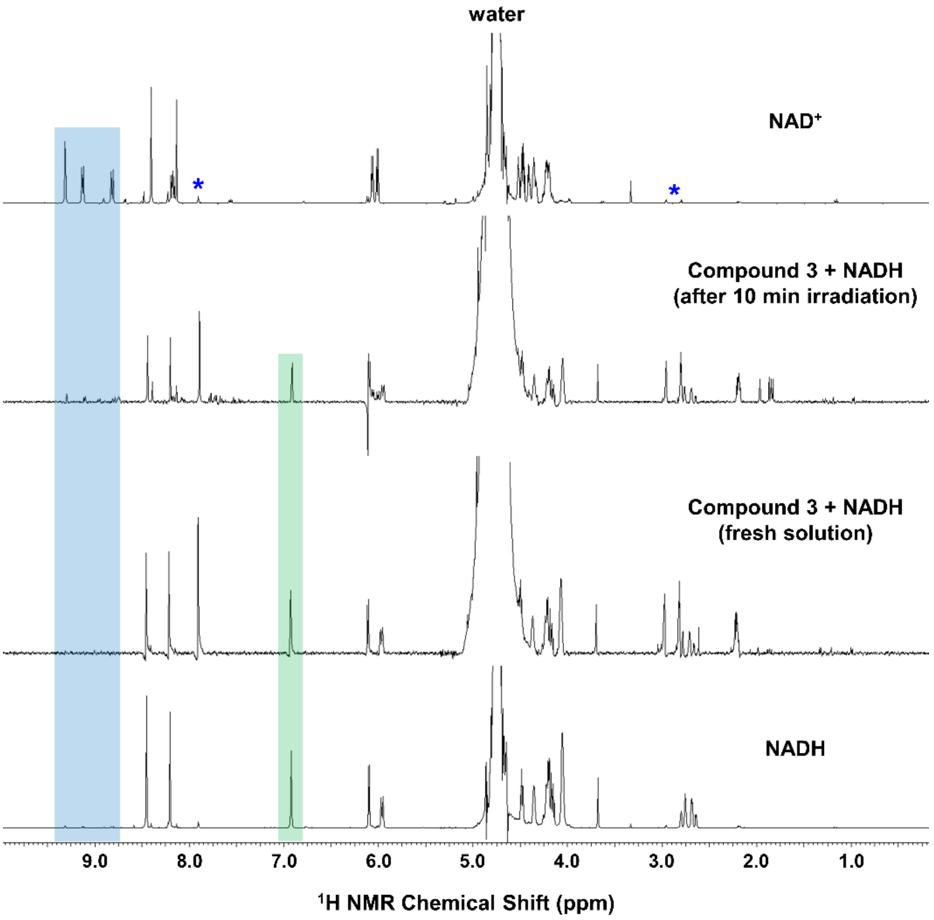

Figure S26. ^{1}H NMR studies of complex **3** mixed with NADH (1:5) in 1% DMF-d_7/99% PBS in D_2O (pH 7.4), as observed on a fresh sample and after 10 min irradiation (blue light). ^{1}H NMR spectra of both NAD(H) compounds are given for comparative purposes (measured in the same medium). Color code: blue - characteristic NAD^+ resonances; green - characteristic NADH resonance; blue asterisks- DMF.

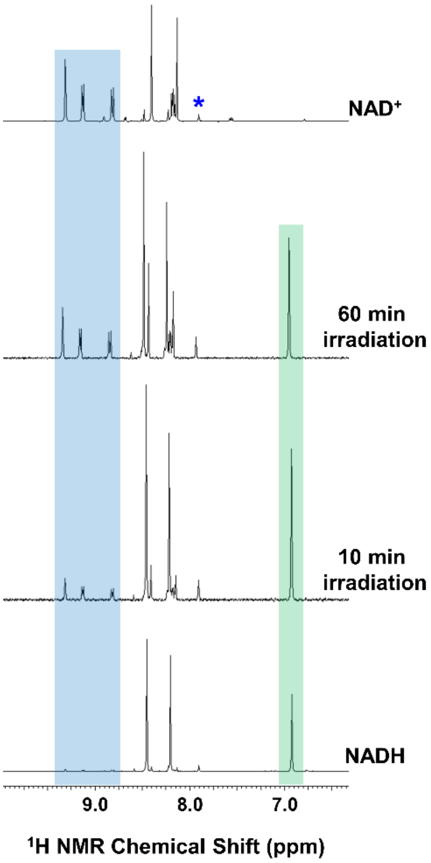

Figure S27. ^{1}H NMR studies of complex **1** mixed with NADH (1:50) in 1% DMF-d_7/99% PBS in D_2O (pH 7.4), as observed after 10 and 60 min of irradiation (blue light). ^{1}H NMR spectra of both NAD(H) compounds are given for comparative purposes (measured in the same medium). Color code: blue - characteristic NAD^+ resonances; green - characteristic NADH resonance; blue asterisks - DMF.

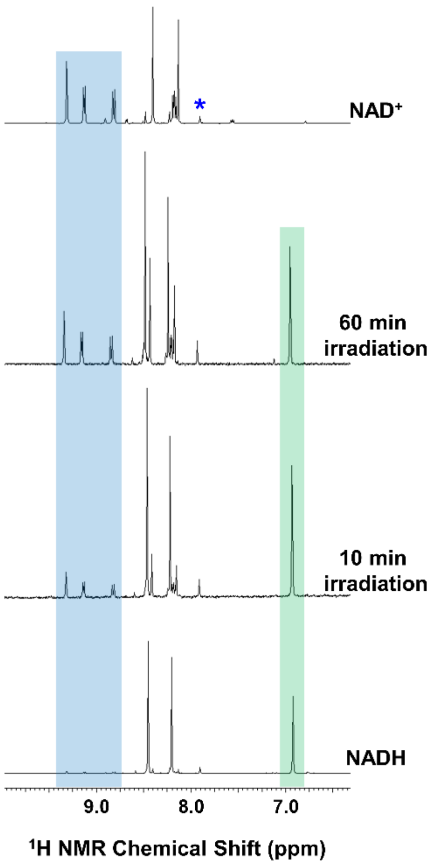

Figure S28. ^{1}H NMR studies of complex **2** mixed with NADH (1:50) in 1% DMF-d_7/99% PBS in D_2O (pH 7.4), as observed after 10 and 60 min of irradiation (blue light). ^{1}H NMR spectra of both NAD(H) compounds are given for comparative purposes (measured in the same medium). Color code: blue - characteristic NAD^+ resonances; green - characteristic NADH resonance; blue asterisks - DMF.

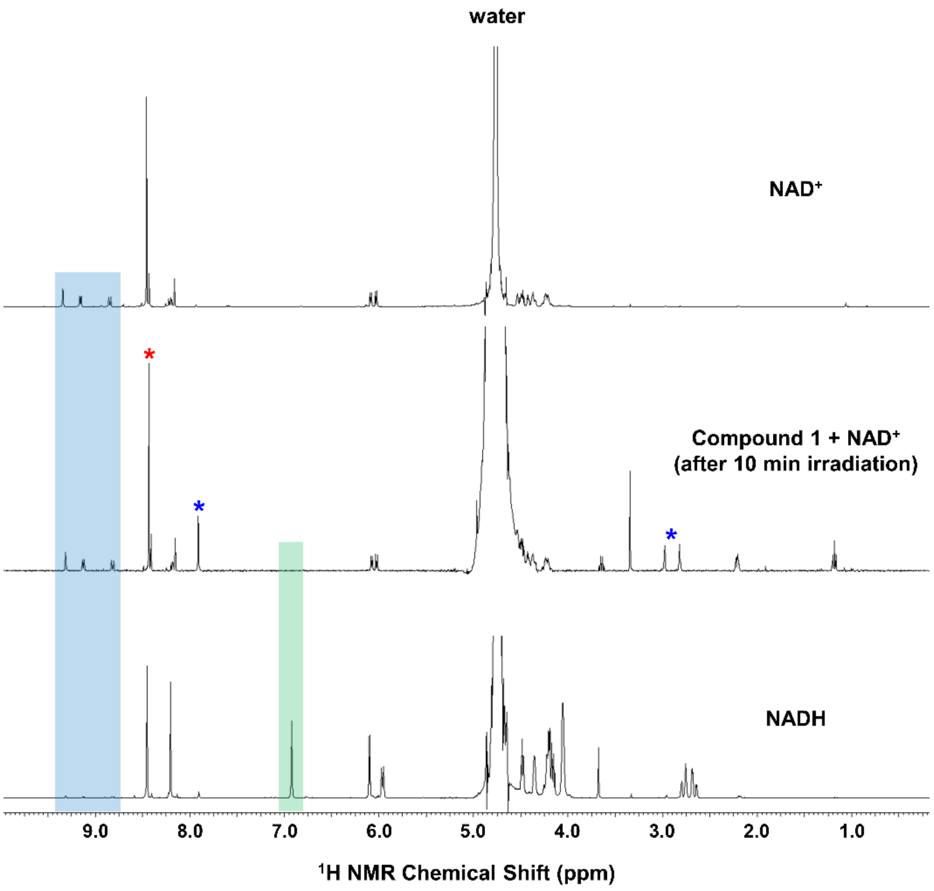

Figure S29. ^{1}H NMR studies of complex **1** mixed with NAD^{+} and sodium formate (1:5:25) in 1% DMF-d_7/99% PBS in D_2O (pH 7.4) in the dark, as observed after 10 min irradiation by the blue light (*middle*). ^{1}H NMR spectra of NAD^{+}/sodium formate (1:5; *top*) and NADH (*bottom*) are given for comparative purposes (measured in the same medium). Color code: blue - characteristic NAD^{+} resonances; green - characteristic NADH resonance; red asterisks - sodium formate; blue asterisks - DMF.

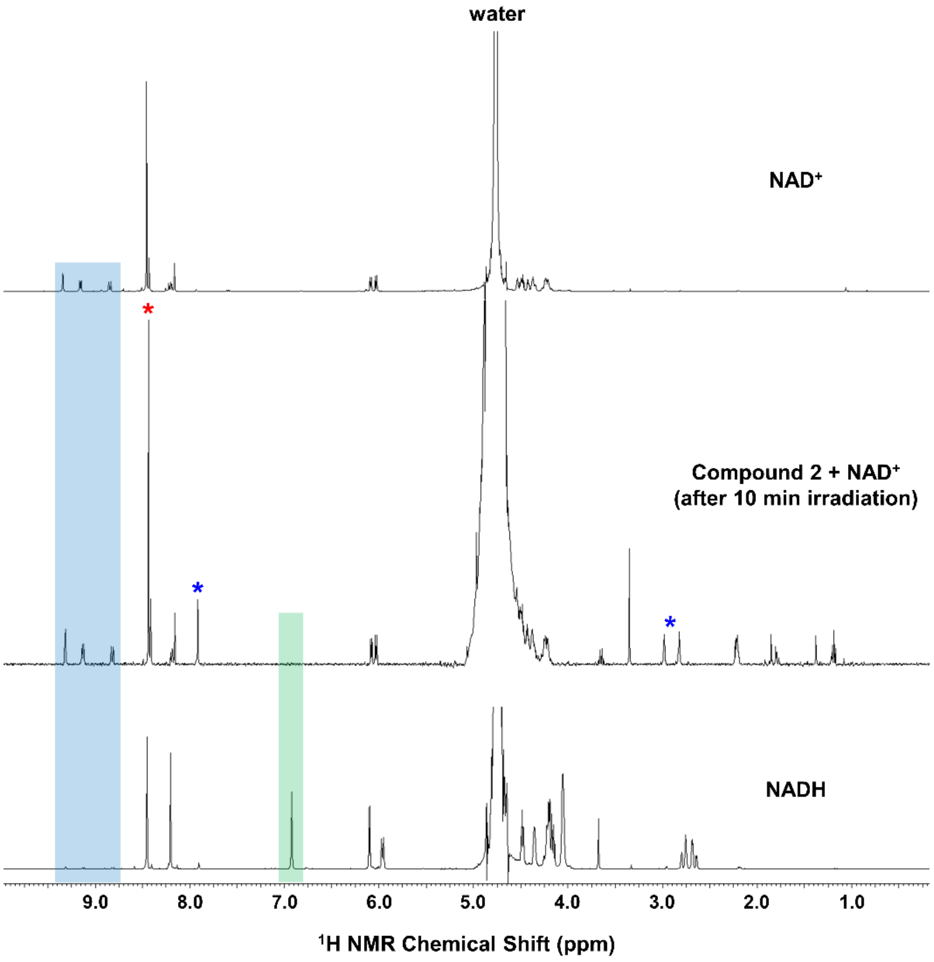

Figure S30. ^{1}H NMR studies of complex **2** mixed with NAD$^+$ and sodium formate (1:5:25) in 1% DMF-*d*$_7$/99% PBS in D_2O (pH 7.4) in the dark, as observed after 10 min irradiation by the blue light (*middle*). ^{1}H NMR spectra of NAD$^+$/sodium formate (1:5; *top*) and NADH (*bottom*) are given for comparative purposes (measured in the same medium). Color code: blue - characteristic NAD$^+$ resonances; green - characteristic NADH resonance; red asterisks - sodium formate; blue asterisks -DMF.

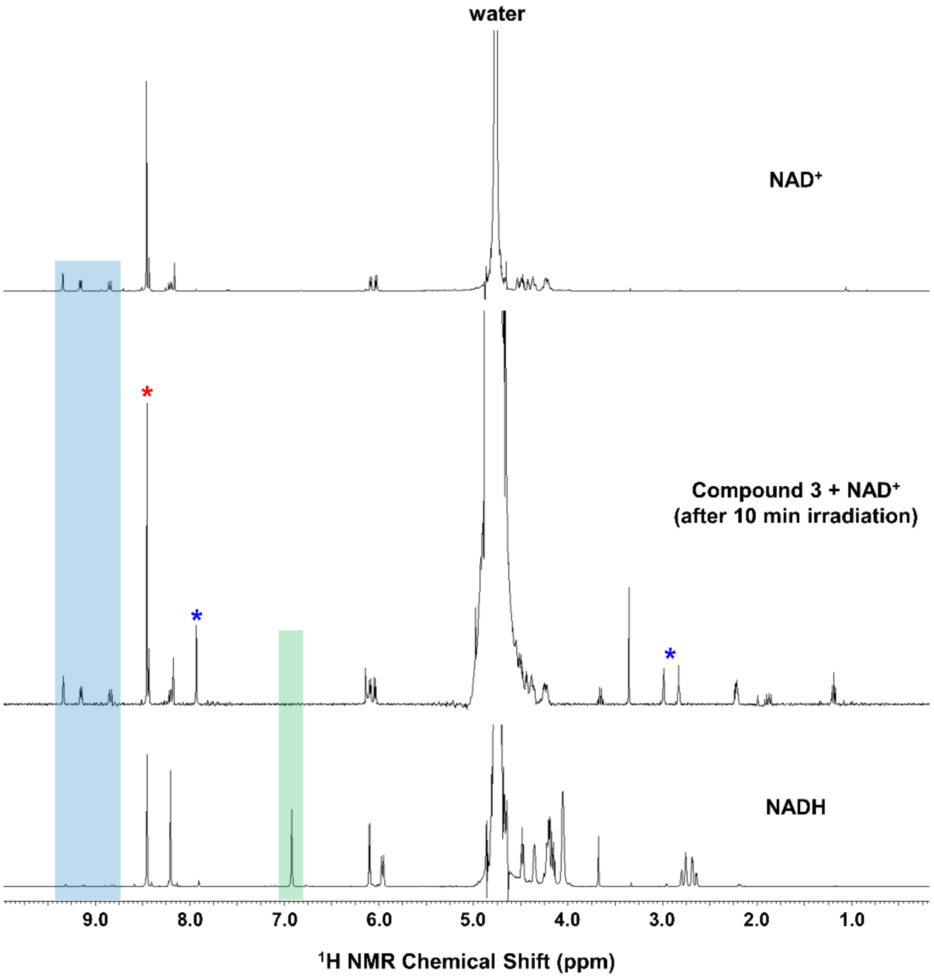

Figure S31. ^{1}H NMR studies of complex **3** mixed with NAD^{+} and sodium formate (1:5:25) in 1% DMF-*d*$_7$/99% PBS in D$_2$O (pH 7.4) in the dark, as observed after 10 min irradiation by the blue light (*middle*). ^{1}H NMR spectra of NAD^{+}/sodium formate (1:5; *top*) and NADH (*bottom*) are given for comparative purposes (measured in the same medium). Color code: blue - characteristic NAD^{+} resonances; green - characteristic NADH resonance; red asterisks - sodium formate; blue asterisk-DMF.

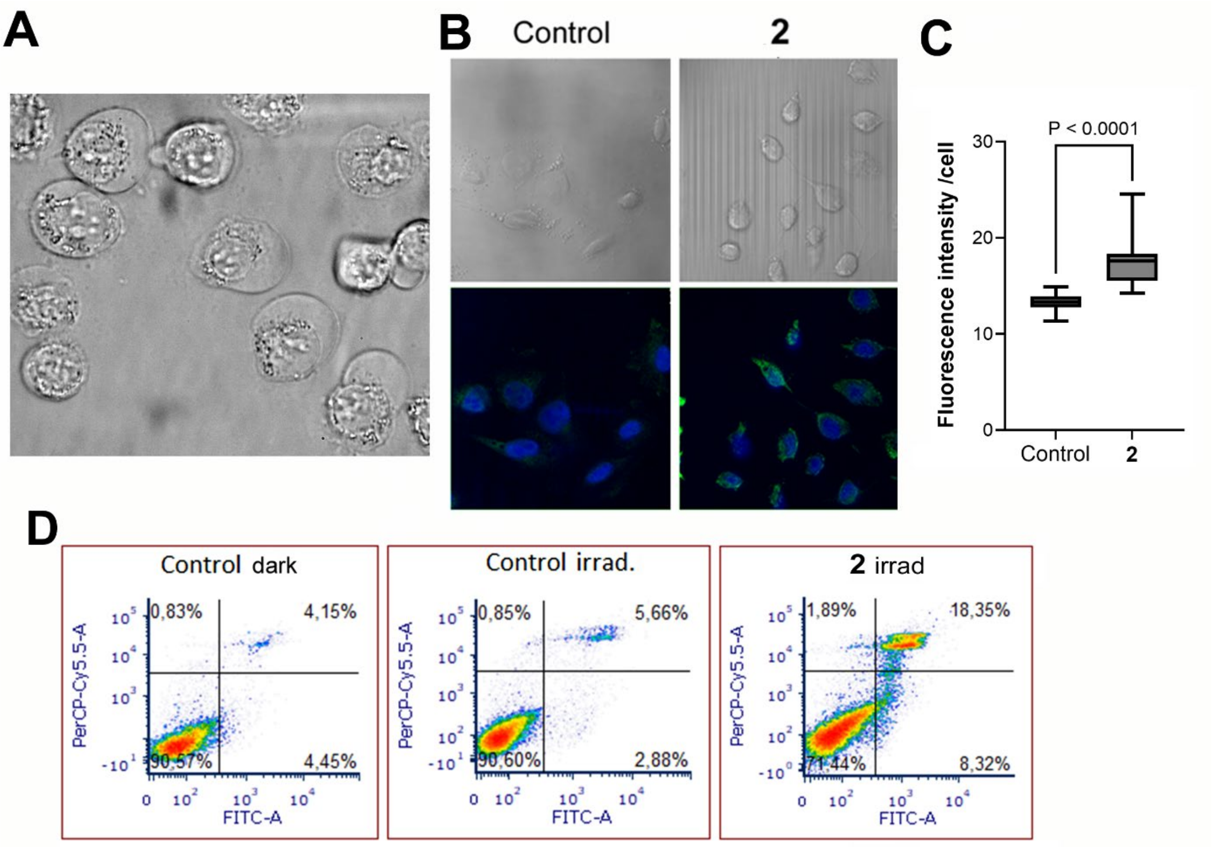

Figure S32. A. Microscopic images of A375 cell morphology, showing vacuolization of cytoplasm and cell swelling, as observed under an inverted optical microscope. Cells were treated with **2** (0.5 µM) or left untreated for 1 h, irradiated, and then incubated in compound-free medium for 30 min. B. Immunofluorescence staining of porimin in A375 cells, untreated or treated with complex **2** (0.5 µM), irradiated with blue light, and incubated with Rh-free media for 4 h. Top panels, bright field; bottom panels, merged signals from blue and green fluorescence channels. Porimin appears as a green fluorescence signal; the nuclei of the cells are stained with DAPI (blue). C. Quantitative evaluation of fluorescence intensity in cells immunolabeled for porimin. At least 25 cells in four different fields were included in the evaluation. Statistical significance was determined using the Mann-Whitney test. D. Representative density plots of cells after their PI/annexin V FITC staining. Before staining, cells were incubated with complex **2** (0.4 µM) and irradiated with blue light.

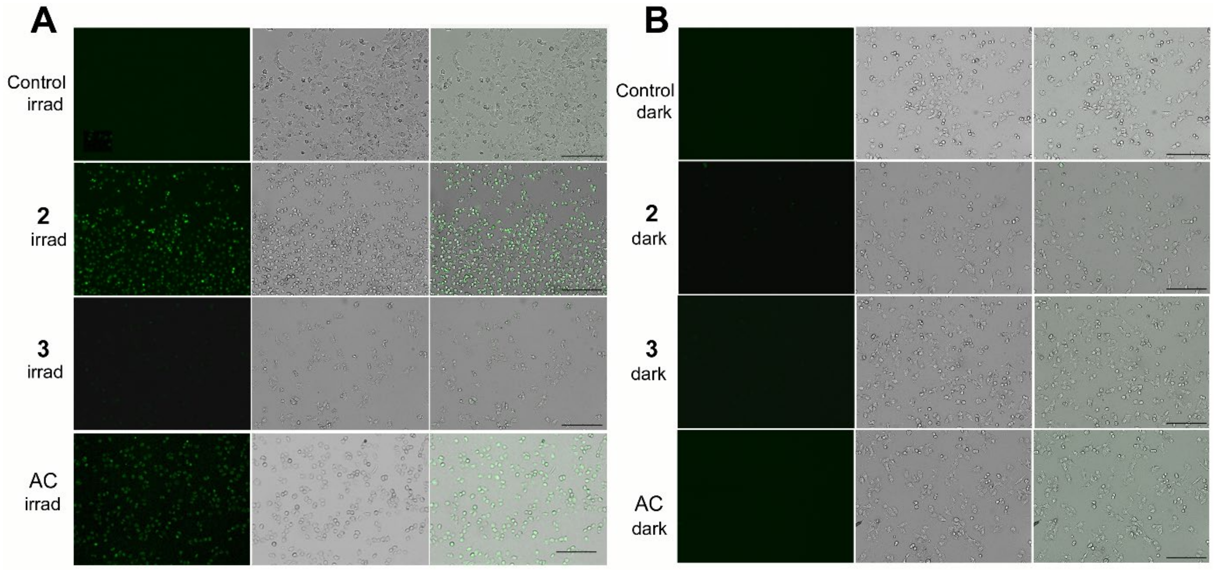

Figure S33. Detection of ROS generation in A375 cells, untreated (control) or treated with **2, 3,** or AC (0.5 µM). A. Irradiated samples. B. Samples kept in the dark. Left panels in Figures A and B: green fluorescence channel; central panels: bright field; right panels: overlay of the bright field and fluorescence channels. Scale bars = 200 µm.

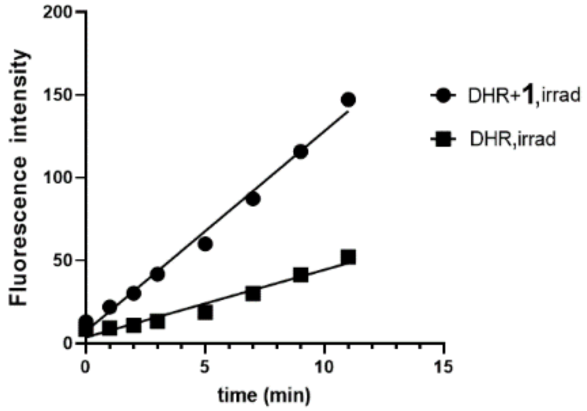

Figure S34. Intensity of fluorescence emission (λ_{ex} = 488 nm, λ_{em} = 530 nm) of DHR 123 alone or in a mixture with complex **1** (10 µM) when irradiated with blue light, as a function of irradiation time.

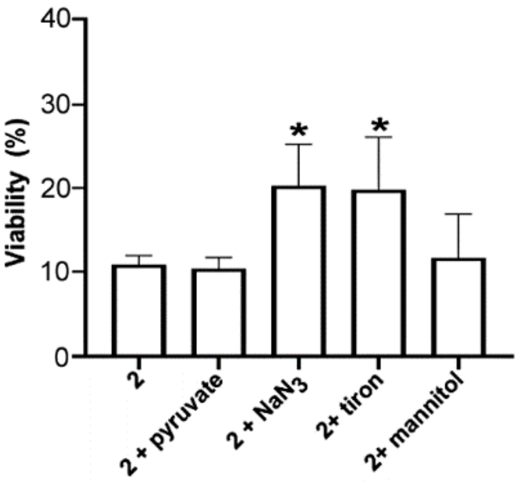

Figure S35. Viability of A375 cells treated and irradiated with complex **2** (0.5 µM) in the presence of ROS scavengers, sodium pyruvate (10 mM), sodium azide (5 mM), tiron (5 mM, and D-mannitol (50 mM). Viabilities of cells irradiated in the medium with the respective scavenger but without complex **2** were taken as 100%. * = significantly different ($p < 0.05$) from viability of cells treated and irradiated in the absence of any scavenger, as determined by the Mann-Whitney nonparametric test, n = 4-6.

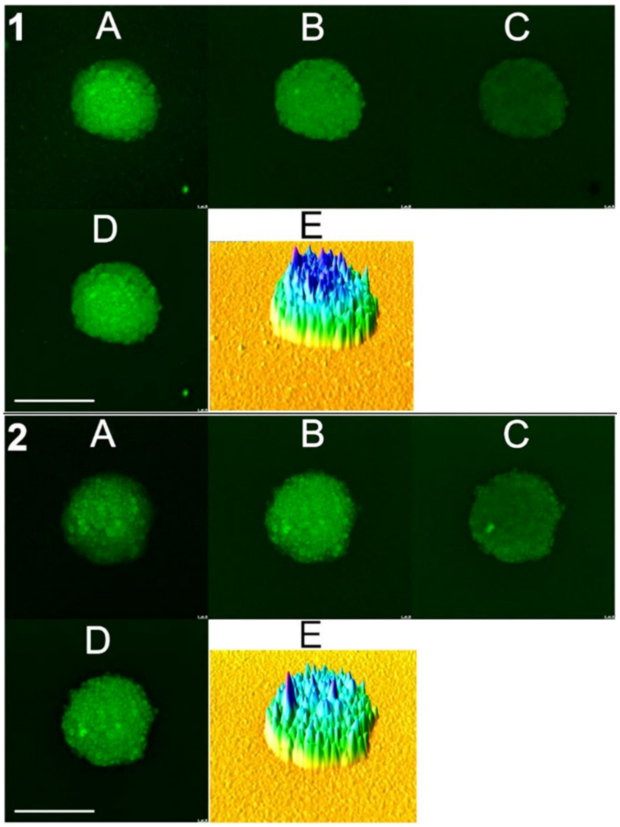

Figure S36. Distribution of complexes **1** and **2** in HCT116 spheroids visualized by confocal microscopy. Spheroids were treated with 15 µM of complex **2** (Panel 1) and **1** (Panel 2) for 3 h. Panels A, B, and C show representative z-slices from the up, upper-mid, and mid regions of the spheroid. Panel D displays z-stack fluorescence intensity projections. Panel E presents a 3D surface plot illustrating the maximal fluorescence intensity projection from the z-stack. Scale bars: 200 µm.

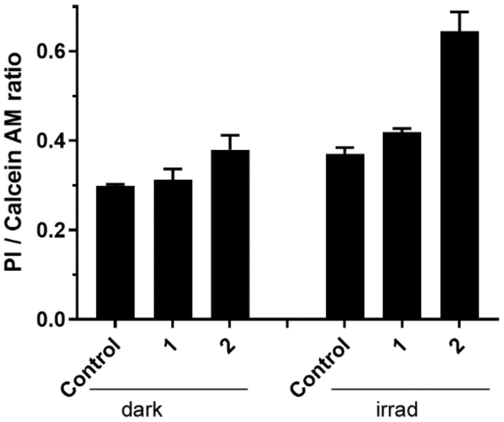

Figure S37. Analysis of the ratio of mean fluorescence intensity of PI and Calcein AM associated with HCT116 spheroids treated with **1**, **2**, or untreated control (see Figure 9). Fluorescence intensity was analyzed in the maximal projection of the obtained z-stacks on confocal microscopy. Data represent mean and SEM from two independent experiments.

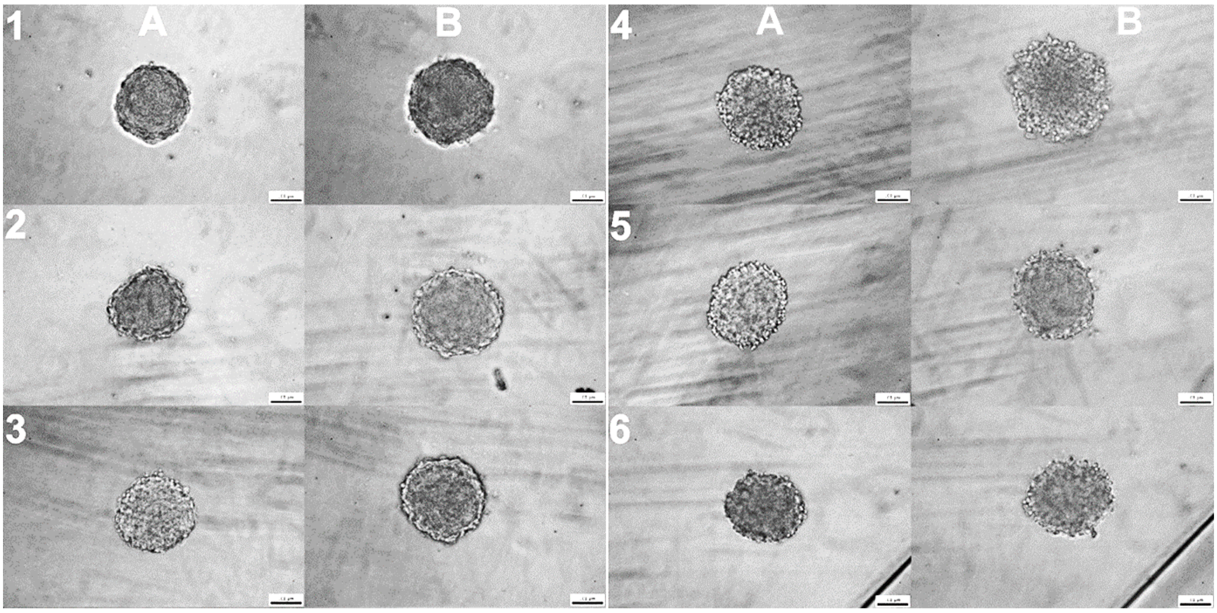

Figure S38. Phase contrast microphotographs of HCT116 spheroids treated with the tested complexes and blue light irradiation. HCT116 spheroids were formed over 48 h under ultra-low attachment conditions. Spheroids were then treated with 30 µM of the indicated complex (**1** or **2**) or left untreated (control) for 5 h. During the final 5 min of the 5-h treatment period, samples designated as "Irradiated" were exposed to 405 nm blue laser light (1 mW, 5 min), while samples designated as "Dark" were kept in the dark. The figure shows phase contrast images of the following samples: Panels: 1) Control (untreated) – Dark, 2) Control (untreated) – Irradiated, 3) **2** – Dark, 4) **2** – Irradiated, 5) **1** – Dark, 6) **1** – Irradiated. Column A shows representative images of the spheroids immediately after the 5-h treatment/irradiation period. Column B shows representative images 19 h post-treatment/irradiation period. Scale bar: 100 µm.
